# Influence of water temperature on feed intake, appetite control, and energy allocation in Atlantic salmon (*Salmo salar*) post-smolt

**DOI:** 10.3389/fphys.2025.1646208

**Published:** 2025-08-13

**Authors:** F. Lai, S. Budaev, I. K. Hundven, P. Balseiro, S. O. Handeland, I. Rønnestad

**Affiliations:** Department of Biological Sciences, University of Bergen, Bergen, Norway

**Keywords:** Atlantic salmon, temperature, appetite and energy expenditure, ghrelin, feed intake and FCR

## Abstract

For ectothermal animals, such as the teleost Atlantic salmon (*Salmo salar)*, temperature is a key environmental factor that influences metabolism, energy allocation and growth. However, the complex interactions among feed intake, appetite-regulating signalling pathways, gastrointestinal transit rates, and energy partitioning toward metabolism and growth across varying water temperatures remain poorly understood. In this study, feed intake, feed efficiency, somatic indices and growth were examined in Atlantic salmon post-smolts (ca. 200 g) acclimated to either 8°C, 12°C or 15°C for 8 weeks. Following the trial, a 24-h postprandial assessment was conducted to evaluate gastrointestinal (GI) transit, plasma metabolite dynamics and responses of appetite regulatory mechanisms. Feed intake (*FI*) and feeding rate (*FIR*) increased with temperature. A bell-shaped growth response was observed, with condition factor (*K*), specific growth rate (SGR), and relative growth rate (RGR) peaking at 12°C. Post prandial analysis revealed greater gastrointestinal content and faster GI-tract transit and feed processing rates at the highest temperatures. Notably, the most rapid and pronounced gallbladder refilling response was observed at 15°C. Elevated temperatures also enhanced postprandial metabolic responses of glucose, lactate, cholesterol, and triglycerides following digestion and processing of the ingested meal. Plasma ghrelin (Ghrl) levels decreased significantly at elevated temperature. Temperature negatively affected the *ghrl1* expression in the stomach, and the expression dynamics of the orexigenic neuropeptide *npya* and anorexigenic *pomca* paralogues in the hypothalamus, which were lower at 15°C. Male maturing fish were observed with the increase of temperature, which associated with reduced feed intake and metabolic acclimations for reproductive development. These temperature-dependent physiological responses highlight the complex interplay between environmental factors and physiological acclimations in Atlantic salmon. A comprehensive understanding of these mechanisms is essential for optimizing growth performance and adaptive capacity in changing thermal environments.

## Introduction

Atlantic salmon is an ectotherm vertebrate, and temperature is one of the predominant environmental factors shaping the fish physiological performance. Thermal tolerance is typically represented as bell-shaped curves, where metabolism-related activities peak within an optimal temperature range and diminish outside of it, ultimately reaching zero at the upper and lower critical temperatures (Kinne, 1960; Miller and Stillman, 2012). Acclimation to moderate temperatures is generally attainable through modifications and regulation of fish behavioural and physiological processes. Typically, voluntary feed intake, digestion and nutrient absorption rise with elevated water temperatures and declines when temperatures drop beyond the species-specific optimum range (Kinne, 1960; Huey and Stevenson, 1979; Volkoff and Rønnestad, 2020; Jutfelt et al., 2021). Such adjustments in feed consumption and processing enables allocation of energy resources to sustain the individuals fundamental biological activities (e.g., locomotion, growth, reproduction), while also ensuring sufficient energy reserves to periods marked by variations in metabolic demand (Volkoff and Rønnestad, 2020; Jutfelt et al., 2021).

Hunger and feed intake in fish are controlled by the synergistic action of endocrine signalling from the brain and peripheral organs (e.g., intestine, liver, pancreas), with species-specific response mechanisms varying among teleosts depending on their feeding biology strategies (Volkoff, 2016; Rønnestad et al., 2017). When it comes to the integration of peripheral information related to the nutritional status and homeostatic regulation of energy balance in fish, the hypothalamus, in the brain, serves as the major feeding centre (Rønnestad et al., 2017; Soengas et al., 2018; 2024). Similarly, the gut-brain axis, which involves bidirectional communication between the gastrointestinal tract and the central nervous system, is essential for regulating feed intake and energy homeostasis (Blanco et al., 2021). Although the data is less clear than in mammals, it is believed that stomach secrets ghrelin into circulation, in part in correlation to stomach filling, and after reaching the brain directly stimulates the action of agouti-related protein (Agrp) and neuropeptide Y (Npy) expressing neurons, thereby enhancing appetite (Rønnestad et al., 2017). Conversely, the hormone leptin, secreted into the bloodstream by the liver, may stimulate the activity of neurones that express pro-opiomelanocortin (Pomc) and cocaine- and amphetamine-regulated transcripts (Cart) peptides, thereby decreasing appetite (Rønnestad et al., 2017). These signalling appetite pathways and their regulation in Atlantic salmon have been commonly correlated with nutrients availability (Kalananthan et al., 2020b; 2020a; 2021; 2023; Del Vecchio et al., 2021; Tolås et al., 2021; Lai et al., 2025). The correlation between feed intake and environmental temperature has been described from an energy allocation perspective, where fish adjust their feeding uptake to optimize the energy metabolism under carrying thermal conditions (Jutfelt et al., 2021). Nevertheless, the complex interaction between the hypothalamus and the gastrointestinal tract in regulating feed intake and energy homeostasis at varying temperatures in Atlantic salmon is unexplored.

**FIGURE 1:**
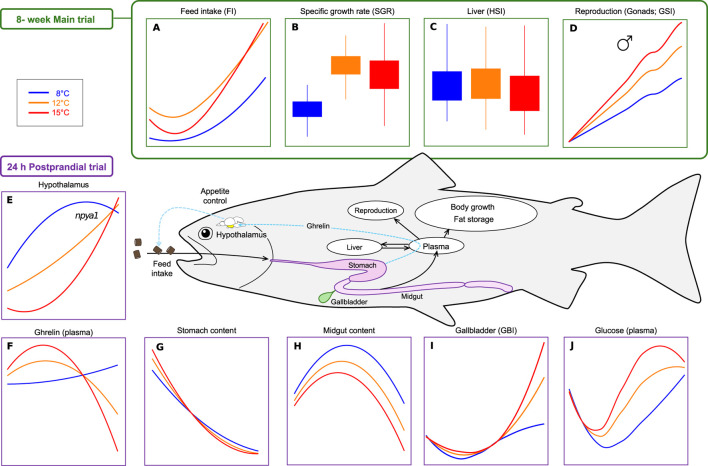
Summary of key findings from the 8-week main trial **(A–D)** and 24-h postprandial trial **(E–J)** in Atlantic salmon held at three temperatures (8°C, 12°C, 15°C). The schematic in the center illustrates the physiological compartments and pathways involved in feed intake regulation, digestion, energy allocation, and growth, highlighting key parameters measured in the study. **(A)** Feed intake (FI; feed eaten (g)/day); **(B)** Specific growth rate (SGR; %), **(C)** Liver-Hepatosomatic index (HSI; %); **(D)** Reproduction-Gonadosomatic index (GSI; %); **(E)** Hypothalamic *npya1* expression (mRNA copy number/ng Tot. RNA); **(F)** Plasma ghrelin levels (ng/ul); **(G)** Stomach content (%stomach dry content/fish body weight); **(H)** Midgut content (% midgut dry content/fish body weight), **(I)** Gallbladder index (GBI; %); **(J)** Plasma glucose levels (mmol/L). Details and specifics of all parameters are provided in the Supplementary Material.

This study aimed to gain a comprehensive understanding of how different acclimation temperatures impact Atlantic salmon appetite regulatory mechanisms and growth. Over an 8-week period, feed intake, feed efficiency, somatic indices and growth were examined in post-smolts (average body weight 200 g) exposed to three temperature regimes: 8°C, 12°C and 15°C. At the end of the trial, a 24 h postprandial assessment was conducted following a single meal fed to satiety, focusing on the temperature effects on the short-term appetite regulation, plasma metabolites dynamics, and gastrointestinal transit. The principal findings are synthesised in Figure 1 to facilitate interpretation and guide the ensuing discussion. The details and specifics of all parameters are provided in the results section and in Supplementary Material.

## Materials and methods

### Ethics statement

The study complied with the guidelines of the Norwegian Regulation on Animal Experimentation and was approved by the National Animal Research Authority in Norway (FOTS ID 28416).

### Fish and experimental design

The experiment was carried out at the Department of Biological Sciences, University of Bergen, Norway, between January and April 2022. Atlantic salmon post-smolts of approximately 200 g were PIT tagged and randomly distributed into nine tanks (volume ca 600 L) of 62 fish per tank. The tanks were supplied with flow-through seawater maintained at a constant temperature of 10°C ± 0.00°C, 28‰ salinity, oxygen saturation above 90% and LD12:12 light regime (from 07:00 to 19:00). After a 3-week acclimation period, the water temperature was gradually adjusted over 72 h to establish three experimental temperature groups (three tanks/treatment): 8°C, 12°C and 15°C. Water temperature, salinity, and oxygen saturation were monitored daily and maintained at constant levels for 8 weeks (Supplementary Table S1). Fish were fed a 3 mm commercial pelleted diet (NOFIMA, Norway) once daily using automatic conveyor-belt feeders, operating for 2 hours, from 8:00 to 10:00. The LD12:12 light schedule was used to signal the upcoming feeding event, with lights switched 1 hour before feeding. Feed was offered in excess, and the daily ration administrated to each experimental group was calculated based on estimated fish biomass, assuming a daily growth rate of 1.2% and a feed conversion ratio (FCR) of 1.2 during the first 4 weeks and 1.4 during the final 4 weeks. The daily amount of feed was weighed with a two-digit scale and placed on the conveyor belt feed the day before to minimise any disturbance before feeding. Uneaten feed was collected and weighed at the end of each meal to quantify the actual feed intake per tank.

### Sampling procedure

Multiple samplings were conducted, with five primary time points to assess fish size and growth: one prior to temperature exposure (week 0), and 2, 4, 6 weeks thereafter. Each sampling was started precisely at the conclusion of the two hour-feeding time, and five fish per tank were randomly collected and euthanized with an overdose of tricaine (200 mg/L NaCO3-buffered Finquel®vet). Fork length and weight were recorded, and liver, and gonads were weighed using a precision electronic balance (VWR International AS, Norway) with an accuracy of three significant digits.

On week 8, an additional 10 fish per tank were sampled at 2, 4, 8, 12, 16, 20, 24 h post-feeding (hpf) to assess the short-term impact of temperature on physiological mechanisms involved in regulation of appetite. To minimize the handling stress associated with frequent sampling, fish from each of the three triplicate experimental tanks were lightly anaesthetised (60–80 mg/L NaCO3-buffered Finquel ®vet Argent Chemical Laboratories, Redmond, United States), combined in a 200L holding tank, and randomly redistributed within their original replicate tanks 2 weeks prior the samplings. On the day of sampling, each tank replicate was designated for specific time points: replicate one for 2, 12, and 24 hpf; replicate two for 4 and 16 hpf; and replicate three for 8 and 20 hpf. Each tank thus served as the representative unit for its assigned sampling intervals. Fish were euthanized with an overdose of tricaine (200 mg/L^1^ NaCO3-buffered Finquel®vet). Blood was collected from the caudal vein using 2 mL syringes (BD Plastipak, VWR, Norway) with 23G heparinized needles (Microlance TM3, VWR, Norway) and centrifugated at 5000 rpm for 3 min. The plasma was transferred into 0.6 mL PCR tubes (Axygen, Glendale, Arizona), kept on dry ice, and stored at −80°C until analysis. Fish fork length and weight were measured, and liver, gonads and gall bladder weighed using a precision electronic balance (VWR International AS, Norway) with an accuracy of three significant digits. The gastrointestinal tract was collected and divided into stomach (ST), midgut (MG), and hindgut (HG) by using surgical clamps to ensure that there was no loss or transfer of feed and digesta between these compartments (Kalananthan et al., 2020b). The content from each section was meticulously transferred into pre-weighed plastic bags. The wet content was weighed immediately after each sampling, whereas the dry weight was determined after incubating the samples at 74°C for 24 h to ensure full dehydration. A portion of stomach tissue was collected from each fish, cleaned in 1X phosphate buffered saline (VWR, Oslo, Norway) and stored in tubes with RNA later (Invitrogen, Carlsbad, CA, United States). Whole brains were collected from each fish and immediately placed into tubes containing RNA later. Both stomach and brain samples were refrigerated for 24 h and then transferred to −80°C until further analysis.

After the completion of the multiple samplings, the left-over fish from each experimental group were collected, and their body weight and fork length were recorded as additional data for the final growth and biomass assessment of the experimental groups.

### Calculations

#### Feed utilisation and growth

Absolute feed intake (*FI*) was calculated for each tank for the duration of the 8-week trial as:

$$FI=Feedgiven\;\left(g\right)-Feed\;waste\left(g\right)$$

Further, relative feed intake (*FIR*) was calculated as *FI* relative to the fish body weight (g) (*W*):

$$FIR=\frac{FI\;}{W\;}$$

Because the feed intake was measured on the daily basis while the fish body mass was sampled only every 2 weeks, direct calculation of the relative feed intake *FIR* was not possible for all time points. Therefore, the body weight at the missing time points was approximated using linear interpolation for each tank based on the previous and the next body weight sampling data.

The feed conversion rate (*FCR*) at the conclusion of the trial was calculated using the subsequent equation:

$$FCR=\frac{Feedeaten}{Wf-Wi}$$

Where feed eaten is the overall *FI* at conclusion of the 8-week trial and *W*_*f*_ *-W*_*i*_ is the fish body weight (g) increment from week 0 to week 8.

Fish growth was calculated as Relative Growth Rate (RGR) and Specific Growth Rate (SGR) as follow:

$$RGR\left(\%\right)=\frac{\left(Wf-Wi\right)}{Wi}*100$$

$$SGR\;\left(\%\right)=\left(e^{\frac{\log\left(\text{Wf}\right)‐\log\left(\text{Wi}\right)}{t}}‐1\right)100$$

where *Wf* is the final weight (g), *Wi* is the initial weight in grams of individually PIT-tagged fish, and *t* is time in days.

The fitness of the fish as condition factor (*K*) was calculated using the following equation:

$$K=\frac{W}{l^{3}}$$

where *W* is the weight of the whole fish (g) and l is the fish body length (cm).

The hepatosomatic index (*HSI*) was determined to give an indication of the fish’s energy condition and metabolic activity:

$$HSI\;\left(\%\right)=\frac{Wl}{W}\cdot 100$$

in which *Wl* is the liver weight (g), and *W* is the weight of the whole fish (g).

Assessment of fish sexual maturation was calculated for both sexes using the gonadosomatic index (*GSI*):

$$GSI\left(\%\right)=\frac{Wg}{W}*100$$

where *W*g is the weight of the gonad (g) and *W* is the fish body weight (g). The GSI threshold used to define the onset of maturation were set at >0.06% for males and >0.20% for females. The male GSI threshold was established based on the criteria described by Pino Martinez et al. (2021), Pino Martinez et al. (2023), while the threshold for females was determined from unpublished data from the same authors (Pino Martinez, pers. comm).

### GIT fullness and gall bladder index

The dry content from each GIT section was quantified to assess the level of *GIT* fullness in relation to the experimental condition, and to evaluate the GIT evacuation dynamics after a meal:

$$GITsectioncontent\;\left(\%\right)=\frac{STdw\text{or}MGdw\text{or}HGdw\;}{W-\left(STww+MG\;ww+HGww\right)}*100$$

where *STdw*, *MGdw* and *HGdw* is the dry weight (g) of the stomach, midgut, and hindgut content respectively. While, *W* is the fish body weight (g), and *STww*, *MGww* and *HGww* is the wet content (g) of the stomach, midgut, and hindgut.

The gall bladder fulness index (*GBI*) was calculated as follow:

$$GBI\left(\%\right)=\frac{Wgb\;}{W\;}*100$$

where W*gb* is the gall bladder weight (g) and W is the fish body weight (g).

### Metabolites plasma levels

Plasma metabolite levels were analysed using a Pentra C400 clinical chemistry analyzer (HORIBA, Japan). The parameters of interest were quantified using spectrophotometry and the following reagents: ABX Pentra Glucose HK CP kit for glucose, the ABX Pentra Lactic Acid reagent for lactic acid, the ABX Pentra Triglycerides CP kit for triglycerides, and the ABX Pentra Cholesterol CP kit for cholesterol. A minimum of 150 µL of plasma per sample was required in the device to enable simultaneous analysis of all parameters. All assays were calibrated and quality-controlled according to instructions provided by the manufacturer.

### Ghrelin plasma levels

Plasma level of ghrelin was quantified by a sandwich Enzyme Linked Immunosorbent Assay (ELISA) (MyBiosource*,* San Diego*,* United States) according to the manufacturer’s instructions. Samples were run in duplicates, and the optical density of each well was determined immediately by using a microplate reader set to 450 nm (Tecan, Männedorf, Switzerland). The optical density of each plate was read three consecutive times, and each sample ghrelin concentration was extrapolated from the standard curve by using Akima curve fitting.

### Gene expression

#### RNA extraction and cDNA synthesis

Total RNA from stomach and hypothalamus (for brain dissection, see Kalananthan et al., 2020a) was extracted using TRI Reagent (Sigma-Aldrich, Missouri, United States). A NanoDrop ND-1000 spectrophotometer (Thermo Fisher Scientific, MA, United States) and a 2,100 Bioanalyzer with RNA 6000 Nano Kit (Agilent Technologies, CA, United States) were used to determine the quantity, quality, and integrity of the extracted total RNA, respectively. To remove any remaining genomic DNA, 10 μg of total RNA was processed with the TURBO DNase-free Kit (Ambion Applied Biosystem, CA, United States). cDNA was synthesised from 1.2 to 1.6 μg of total RNA for stomach and hypothalamus, respectively, using SuperScript III Reverse Transcriptase (Invitrogen, CA, United States) and Oligo(dT)20 (50 μM) primers in a 20 μL reaction volume and then combined. The protocols were carried out according to the manufacturer’s instructions.

#### Real time PCR (qPCR)

Atlantic salmon specific primers for *ghrelin 1* (*ghrl1*), *ghrelin 2* (*ghrl2*) and *membrane-bound O-acyltransferase 4* (*mboat*) (Del Vecchio et al., 2021) were used in the stomach, while specific primers for *npya1* and *npya2* (Tolås et al., 2021)*, agrp1, pomca1 and pomca2* (Kalananthan et al., 2020b)*,* and *cart2b* (Kalananthan et al., 2021) were used for the mRNA expression analysis in the hypothalamus (Supplementary Table S2)*.* The primers were checked for quantification cycle (Cq), primer efficiency (E), and melting peaks to identify any possible nonspecific products or primer dimers. The efficiency of the primers was determined by making a 10-fold dilution standard curve ranging from 1.00E+07 to 1.00E+02 copies amplicon/μL cloned into a pCR4-TOPO vector (Thermo Fisher Scientific). qPCR was performed using 10 μL of SYBR Green I Master Mix (Roche Diagnostic, Basel, Switzerland), 0.6 μL of each forward and reverse primers (10 mM), 6.8 μL Ultra-Pure Water (Biochrom, Berlin, Germany) and 2 μL cDNA template (12 ng DNA/reaction for *ghrl1, ghrl2, mboat, npya1*, and *npya2;* 80 ng cDNA/reaction *pomca1*, *pomca2*, *agrp1* and *cart2b*). Samples reactions were run in duplicate into 96-qPCR plates (Bio-Rad Laboratories, CA, United States). All plates included two negative controls, no-template and no-reverse transcriptase, and one positive control (between plate control), and the following qPCR protocol was performed: 1) 95°C for 30 s, 2) 95°C for 5 s, 3) 60°C for 25 s, 4) repeating step two to three for 39 more times. Melting curve analysis over a range of 65°C–95°C (increment of 0.5°C for 2 s) allowed the detection of nonspecific products and/or primer dimers. Subsequently, the copy number for each target gene was determined based on the respective standard curve slope and intercept using the following equation:

$$Copynumber=10^{\left(\frac{Cq-intercept}{slope}\right)}$$

The copy number was normalized to the total ng of RNA used for each target gene.

### Statistical analysis and modelling

Statistical analyses and graphics were performed in R (R Core Team, 2024) using a multi-model inference approach (Burnham and Anderson, 2022). A series of models were fitted including a range of predictor variables and then calculated the second order Akaike information criterion (AIC_c_) assuming lower AIC_c_ indicates better fit. Based on ranked AIC_c_, the Akaike evidence weights (*w*) was calculated for individual ranked models. Finally, the model with the highest evidence weight (i.e., the lowest AIC_c_) was considered the “best” within the family. Whenever the best fitting model was determined, individual beta coefficients and effect size values were analysed to understand the major effects. Additionally, the hypotheses that the model parameters significantly differ from zero was also tested. The linear models were fitted using standard or generalized least squares depending on the residual error structure. When significant serial correlation of residuals was detected (violating the assumption of independence for errors) a generalized least squares based on restricted maximum likelihood was used, adding an *n*-order autoregressive ARMA(*n*) process (Faraway, 2009; Fox, 2016). The optimal *n* was determined by fitting models with a range of *n*s and selecting the model characterized by lowest AIC_c_ as well as by comparing incremental models using analysis of variance (Chambers and Hastie, 1997; Fox, 2016). This was computed using the R ‘gls’ function from the ‘nlme’ package (Pinheiro et al., 2025). For nonlinear estimation, a standard ‘nls’ function was used (Ritz and Streibig, 2008). Model comparison based on Akaike evidence weights was done using the ‘MuMIn’ R package (Barton, 2023).

For all tests, p < 0.05 was considered significant *(*∗*p < 0.05;* ∗∗*p < 0.01;* ∗∗∗*p < 0.001*). All data are presented as mean ± SEM; otherwise, it is stated. More details of the data analysis and notation can be found in the Supplementary Material.

## Results

### Eight-week monitoring trial

#### Fish intake, growth and somatic indices

##### Feed intake

Water temperature significantly affected feed intake across the three experimental groups, exhibiting higher and a greater temporal increase at higher temperatures (*T*, p < 0.0000; *D:T*, p = 0.0004; Supplementary Table S4; Supplementary Figure S2). However, polynomial and linear interaction terms were also significant, making the resulting pattern complicated. This said, the 8°C group always tended to display the lowest FI compared to the 12°C and 15°C (Supplementary Table S4; Supplementary Figure S2). To control for the possible effect of stress on fish caused by the several samplings over the 8 weeks, we introduced a “post stress” dummy factor (*St*: 1 = next day after sampling, 0 = all other days) on the model analysis. A negative stress effect on *FI* right after each sampling event at weeks 2, 4, and six was observed (*St*, p = 0.0001; Supplementary Table S4). Stress-related disruption of feed intake appeared significantly stronger at lowest temperature (*T*:*St*, p = 0.0063; Supplementary Table S4; Supplementary Figure S3).

All experimental groups showed also a significant time effect on the *FIR* (Supplementary Table S7; Supplementary Figure S5). A notable decline in *FIR* during the initial weeks of exposure to the experimental temperature was observed in all three temperature groups, followed by an overall smaller increase towards the end of the trial (quadratic effect *D* ^2^, p < 0.0001; Supplementary Table S7; Supplementary Figure S5). Temperature had a significant positive effect on *FIR,* while sampling stress significantly reduced it (*T*, p = 0.0225; *St* < 0.0001; Supplementary Table S7).

##### Growth and somatic indices

Higher temperature was linked with higher fish body weight (*T*, p = 0.0287; Supplementary Table S10), although the size effect was small (0.08). The best fitting model for body length did not include temperature effect (Supplementary Table S11) indicating that temperature had no effect upon it. A notable temperature effect was recorded on *K* factor (*T*, p = 0.00115; Supplementary Table S14; Supplementary Figure S8). The quadratic interaction between week *E* and temperature *T* (Supplementary Table S14) pointed to an increase in the *K* factor from the 8°C–12°C group, with subsequent decline in the 15°C group (effect plot Supplementary Figure S9). Similarly, a quadratic temperature effect was observed for both *SGR* and *RGR* (*T* ^*2*^, p = 0.013; Supplementary Table S16; and *T* ^*2*^, p = 0.0044; Supplementary Table S18 respectively), with the highest growth occurring at 12°C (Supplementary Figures S10, S11). The effect of temperature on *FI*, *FIR* and growth was confirmed by the temperature effect on *FCR*; which diminished as temperature increased (Supplementary Table S19). The somatic indices analysis revealed that the *HSI* decreased during the experimental weeks to then increase towards the end of the trial (quadratic effect *E* ^*2*^; p = 0.0001; Supplementary Table S21; Supplementary Figure S12). In addition, the water temperature had a negative link with *HSI* (*T*, p < 0.0001; Supplementary Table S21; Supplementary Figure S12). *GSI* was characterized by a highly skewed distribution due to instances of male maturing fish (Supplementary Table S23). The male *GSI* score increased over the weeks of the trial (Supplementary Figure S13) as evidenced by the interaction *S*_*m*_ *:E:T*; p < 0.0001; Supplementary Table S23).

#### 24 h postprandial monitoring trial

##### GIT transit and gall bladder index

##### Stomach

A quadratic and exponential decay models in model comparison (Supplementary Table S24) were included and were fitted using nonlinear estimation. Overall, quadratic model showed the best fit, which points that the assumption of constant passage rate (as in the exponential decay models) does not hold. Despite this, the results were analysed from the best exponential model. Exponential decay model of the form $y_{f}+\left(y_{0}-y_{f}\right)e^{-\alpha x}$ can be very informative because it includes easily mechanistically interpretable parameters: the start *y*_*0*_, the final *y*_*f*_ values and the fixed transit rate α that can be parametrised by the predictor variables. Both models showed convergent results: faster stomach emptying at higher temperature. The quadratic model (see Supplementary Table S25) pointed to a negative link between temperature and relative stomach fullness (parameter *b*, p < 0.01), significant time effect (parameter *c*, p < 0.0033) and significant interaction (parameter *d*, p < 0.0151). The exponential decay model (Supplementary Table S26) revealed significant effects of time (parameter α , p < 0.0014), positive effect of temperature (parameter β , p < 0.0063) and the interaction term (parameter γ , p < 0.0362) on the rate of digesta decay. The final value of relative fullness estimate *y*_*f*_ in the exponential model is negative (Supplementary Table S26), which indicates that many fish should have zero amount of residual material in the stomach at 24h post-meal.

##### Midgut

 The negative effect of temperature on gastric emptying speed was also evident in the midgut passage analysis (Supplementary Tables S27, S28; Supplementary Figure S15). Here the lowest temperature group demonstrated consistently higher midgut fullness, implicating slower absorption dynamics. In the best fitting model (Supplementary Table S28) this effect is obvious due to the significant interaction between temperature and time post-meal (*T:H*, p < 0.0000; Supplementary Table S28), pointing that the temperature effect spreads unequally over the time post-meal. This resulted in a greater amount of content remaining in the midgut during a later stage of digestion in the 8°C group then 12°C and 15°C: the former group still presented more than twice the amount of content compared with 12°C and 15°C 24 h post-meal (Supplementary Figure S15).

##### Hindgut

A strong quadratic interaction of time and temperature was observed for the hindgut content (*T:H* ^*2*^; p < 0.00001) where the absence of main temperature effect and linear interaction (Supplementary Table S30) points to divergent patterns characteristic at different temperatures (Supplementary Figure S16).

##### GBI

The *GBI* exhibited significantly differences among the experimental groups, demonstrating a positive effect of temperature on the gall bladder content (*T*; p < 0.00001; Supplementary Table S32; Supplementary Figure S17). Furthermore, significant temperature and time interactions were identified with strongest quadratic effects. Rapid the gallbladder refilling was observed 16 h post-meal in the high temperature groups (see Supplementary Table S32; Supplementary Figure S17).

#### Metabolites and ghrelin plasma levels

Temperature had a positive effect on glucose, cholesterol and lactate levels (Supplementary Figures S18, 19, 20), even though the effect size was not large (<0.4). Significant polynomial interaction terms indicated that temperature affected the temporal pattern and its curvature (Supplementary Tables S34, 36, 38; Supplementary Figures S18-S20). It appears that glucose, cholesterol and lactate had elevated plasma levels at higher temperatures and slower dynamics at the lowest temperature. Similarly, a significant polynomial interaction between temperature and time was also observed for the triglyceride’s plasma levels (*T:H* ^*2*^, p < 0.00001; Supplementary Table S40), where higher and faster dynamics were visible with increased temperature (Supplementary Figure S21).

Different temporal trends in Ghrl plasma levels were observed for the different temperature groups (*T:H*, p < 0.01; Supplementary Table S42; Supplementary Figure S22). Lower temperature was associated with flatter temporal response, while fish at 15°C showed the most pronounced convex temporal dynamics. Ghrl plasma levels declined significantly from 12 to 24 hpf in the 12°C and 15°C groups, while the 8°C group showing the minimal change.

#### Stomach mRNA expression levels

The mRNA level of *ghrl1* was influenced by the time after meal (*H* and *H* ^*2*^, p < 0.05; Supplementary Table S44; Supplementary Figure S23), as well as by the interactions between temperature and timing (*T:H*, p < 0.0060; Supplementary Table S44; Supplementary Figure S23). The levels of *ghrl1* showed a gradual temperature-dependent decrease in expression after feeding. By the conclusion of the trial, the highest *ghrl1* level was recorded in fish at 8°C and the lowest at 15°C. Similar trends were noted in the mRNA levels of *ghrl2* and *mboat* throughout the post-feeding time (*H* ^*2*^, p < 0.05; Supplementary Tables S46, S48; Supplementary Figures S24 and S25), however no temperature effects or interactions were seen.

#### Hypothalamic mRNA expression levels

Statistically changes in mRNA expression of hypothalamic genes were observed in response to the different experimental temperatures and time after feeding (p < 0.05). A significantly increase in mRNA levels of *npya1* post-feeding was observed in all three experimental groups (*H* ^*2*^, p = 0.01; Supplementary Table S50), but the dynamic curvature over time (interaction between time and temperature) had opposite pattern in the fish at 8°C (convex) compared to the 15°C group (concave) (*T:H* ^*2*^, p = 0.0050; Supplementary Table S50; Supplementary Figure S26). In addition, *npya1* response was lower at higher temperature (*T*, p < 0.00001; Supplementary Table S50; Supplementary Figure S26). The *npya2* mRNA level response following feeding exhibited a linear increase over time (*H*, p < 0.00001; Supplementary Table S52), while an inverse mRNA levels response to temperature was observed (highest at 8°C, lowest at 15°C) (*T*, p < 0.00001; Supplementary Table S52; Supplementary Figure S27). Similarly, the *pomca1* and *pomca2* mRNA levels showed quadratic patterns over time (*H* ^*2*^, p < 0.05) with significant negative effects of the temperature (higher levels at 8°C; *T*, p = 0.01; Supplementary Tables S54 and S56; Supplementary Figures S28, S29). The mRNA levels of *agrp1* and *cart2b* only increased over time without significant effect of the temperature (H, p = 0.05; Supplementary Tables S58, S60; Supplementary Figures S30, S31).

#### Maturation

Maturing males with *GSI* > 0.06% (Supplementary Table S61) were registered along the trial and their incidence appeared more frequently than by chance at 15°C (Fisher’s exact test: p = 0.027). Maturation was associated with reduced body weight (*M*, p < 0.0001; Supplementary Tables S62, S63) and length (*M*, p < 0.0001; Supplementary Tables S64, S65) but higher *K* factor (*M*, p = 0.029; Supplementary Tables S66, S67). No effect on the HSI was observed (Supplementary Table S68). A maturation effect was noted in the plasma metabolite levels, with reduced glucose (*M*, p = 0.0142; Supplementary Tables S78, S79), higher cholesterol levels (*M*, p = 0028; Supplementary Tables S80, S81) and higher triglycerides (*M*, p ≤ 0.0027; Supplementary Tables S83, S84) compared to the non-maturing fish.

Additionally, maturing males also had lower content in the stomach ( μ p < 0.0001; Supplementary Tables S70, S72) and mid- and hindgut (*M*, p < 0.05; Supplementary Tables S74 and S76). In the exponential decay model (Supplementary Table S72), maturing fish demonstrated significantly reduced stomach emptying rate $\left(\mu \right.$ , p < 0.001). No effect of maturation was observed on stomach gene expression (*ghrl1*, *ghrl2*, *mboat*) (Supplementary Tables S86-S88). However, maturing males showed significantly lower mRNA levels of *npya1*, *pomca1*, *pomca2*, and *agrp1* (negative beta weights for *M*, p < 0.05; Supplementary Tables S90, 93, 95).

## Discussion

In the present study, the complex and non-linear relationships between environmental temperature and several physiological processes were examined in Atlantic salmon, including feed intake, gastrointestinal transit rates, appetite-regulatory pathways, and growth performance. The main findings are summarized in Figure 1. An increase in temperature was found to promote *FI*, *FIR*, gastrointestinal transit rates, and postprandial metabolic dynamics of glucose, cholesterol, lactate, and triglycerides likely in response to increased feed processing, digestion and absorption rates. Temperature-dependent responses were observed in the appetite regulatory signaling pathways, including plasma levels of Ghrl, expression levels of *ghrl1* in the stomach, and of *npy* (*a1* and *a2*) and *pomc* (*a1* and *a2*) in the hypothalamus. A bell-shaped growth response was observed, with *K, SGR* and *RGR* peaking at 12°C, and a significant temperature incidence of maturing males was observed.

### Fish intake, growth and somatic indices

Temperature significantly affected feed intake across all three experimental groups during the 8-week trial, as previously reported in other studies on Atlantic salmon (Handeland et al., 2008; Elliott and Elliott, 2010; Kullgren et al., 2013). An initial decrease in *FI* and *FIR* was observed in response to exposure to the experimental temperatures. Nevertheless, the 12°C and 15°C groups appeared to adapt more rapidly than the 8°C group to the new temperature condition, as evidenced by the earlier and steeper increase in *FI* and *FIR* the following weeks. A transient decrease in feed intake was also observed in the days that followed the monitoring sampling, indicative of stress from handling (Madaro et al., 2015; Lai et al., 2023). The impact of stress appeared to be temperature-dependent, with fish in the 8°C group being more affected and exhibiting lower *FI* and *FIR* than those in the 12°C and 15°C groups. The differences in *FI* and *FIR* among the experimental groups were also reflected in feeding efficiency, as indicated by a *FCR* of 0.96 for the 8°C group, compared to *FCR*s of 0.77 and 0.79 for the 12°C and 15°C groups, respectively.

The differences in feed intake observed across the experimental temperatures were reflected in the growth performance of the fish during the 8-week trial. No significant differences in body length increase were detected between groups; nevertheless, body weight, *K*, *SGR* and *RGR* increased with increasing temperatures, with the highest peak registered in the 12°C group. A similar bell-shaped growth response to temperature has been reported in previous studies on Atlantic salmon post-smolts, with the optimal growth temperature varying depending on fish body size (Handeland, 2000; Handeland et al., 2003; 2008; Kullgren et al., 2013; Fraser et al., 2025). For instance, a reduction in growth and feed intake was observed at 14°C for 150–300 g post-smolts and above 12.8°C for 70–150 g post smolt under natural light conditions over a 12-week trial (Handeland et al., 2008; Kullgren et al., 2013). Conversely, a temperature threshold adversely affecting growth was identified above 10.5°C in 1.2 kg post-smolts under continuous light, although feed intake data were not collected in that study (Fraser et al., 2025).

A negative correlation was observed between *HSI* and temperature. The *HSI* is commonly used as an indicator of energy status in fish (Sheridan, 1994; Chellappa et al., 1995), and its decline with increasing temperature may reflect adjustments in energy mobilisation to support the increased metabolic demands associated with elevated temperatures. In addition, the *GSI* analysis revealed the presence of maturing males throughout the trial, with a higher incidence in the 15°C group. Maturation is a biological process that alters fish metabolism and redirects energy allocation toward the reproductive system, and temperature has been identified as one of the triggering keys in Atlantic salmon (Fjelldal et al., 2011, Crouse et al., 2022; Pino-Martinez et al., 2023; Fraser et al., 2025).

The fact that the fish in this study were fed in excess suggests that the observed reduction of feed consumption was voluntary. The direct correlation between increased feed intake and elevated temperature can be attributed to the greater energy demands required to support the higher metabolic rate associated with elevated temperatures (Volkoff and Rønnestad, 2020; Jutfelt et al., 2021). Conversely, the declined feed intake and growth observed above the optimal temperature in ectotherms is attributed to a reallocation of energy priorities, a phenomenon known as aerobic protection. In this process, ectothermic organisms reduce their postprandial residual aerobic scope by decreasing meal sizes, thereby modulating the peak specific dynamic action (SDA) response during periods of elevated temperatures (Jutfelt et al., 2021). This adaptive response ultimately results in reduced growth. This phenomenon is supported by several studies, including the tropical clownfish *Amphiprion ocellaris* (Pham et al., 2021; 2022).

It is noteworthy that the findings of this study are based on feeding regimen involving a single daily meal provided to satiety. Consequently, it cannot be excluded the possibility that a different response to the increased energy demand may have occurred if multiple meals have been offered throughout the day. However, in the study conducted by Handeland et al. (2008), a reduction in feed intake and growth in fish between 170–300 g was observed at temperatures exceeding 14°C, despite feeding twice daily for 2 hours per session. Similarly, in the study by Fraser et al. (2025), fish were fed continuously throughout the day, yet a decline in growth was recorded at temperatures exceeding 10.5°C.

### GIT transit and gall bladder filling dynamics

Temperature significantly affected the feed transit rate in the *GIT*, and the emptying and refilling dynamics of the gall bladder during the 24 h post feeding. The identified positive link between feed intake and temperature was evidenced by higher stomach content and evacuation rate at higher temperatures, as also shown by previous studies on Atlantic salmon (Sweka et al., 2004; Handeland et al., 2008). In addition, although the fish at 8°C had the lowest stomach content, they also exhibited the slowest gastric evacuation rate, likely attributable to reduced rates of feed processing and stomach motility at lower temperatures. This is consistent with previous literature, emphasising that water temperature exerts a greater influence than stomach content on the gastric evacuation rate of ectothermic animals (Kawaguchi et al., 2007; Handeland et al., 2008; Jobling, 2011). At 24 hpf all three groups had no residual stomach content, consistent with the findings of Valen et al. (2011) who reported full gastric evacuation within the same time frame in 45 g Atlantic salmon at 8°C . In contrast, Handeland et al. (2008) observed stomach evacuation time of 48–72 h in fish weighing between 70 and 300 g at water temperatures ranging from 6°C to 18°C The disparities in gastric dynamics among the experimental groups were also evident in the delayed evacuation rates in both the midgut and hindgut at 8°C, in contrast to the groups at 12°C and 15°C. The lowest temperature group demonstrated consistently higher midgut fullness, reflecting the slower stomach emptying and the slower digestion, absorption and motility dynamics in the midgut and hindgut. Consistent with this tendency, the analysis of the GBI revealed both a higher bile content and faster refilling of the gallbladder at higher temperatures, with 15°C exhibiting the most pronounced and rapid response through the 24 hpf.

### Metabolites and ghrelin plasma levels

Elevated temperatures were associated with an increased and accelerated post-prandial metabolic response, as evidenced by higher plasma levels of glucose, lactate, cholesterol, and triglycerides, likely reflecting enhanced feed processing and digestion activity. A delayed homeostatic pattern of glucose relative to triglycerides was observed, underscoring the preference of using lipids over carbohydrates as energy source in salmonids (Polakof et al., 2008; 2011; 2012; Librán-Pérez et al., 2012). Lactate showed a similar, albeit delayed, response pattern to glucose, indicating a possible metabolic connection. The rise of glucose levels in carnivores fish such as rainbow trout (*Oncorhynchus mykiss*) is hypothesised to be mediated and buffered by the immediate conversion of glucose into lactate followed by stimulation of pyruvate metabolism through the Krebs cycle (Polakof and Soengas, 2008; Polakof et al., 2010; Otero-Rodiño et al., 2015). However, the available published data on this mechanism remain limited. Overall, a temperature-dependent effect was distinctly contingent upon the levels of all metabolites and the development of time. At 8°C, the changes in plasma levels were slower and more delayed compared to those observed at 12°C and 15°C.

Temperature effects were also observed for Ghrl levels in plasma. The postprandial response was flat at low temperature; while fish at 12°C and 15°C had a notable reduction in Ghrl plasma levels from two hpf, with the most pronounced decrease in Ghrl levels observed at 24 hpf in the 15°C group. These results do not align with the usual elevation in plasma Ghrl levels prior to the initiation of next meal, as frequently reported in mammals and some teleost species (Higgins et al., 2007; Abdalla, 2015; Volkoff, 2016). Conversely, our data correlate with studies on the species-specific action of ghrelin in salmonids, where ghrelin has been suggested to function as an anorexigenic peptide and inhibit feed intake (Jönsson, 2013). Indeed, our results demonstrated a postprandial downregulation of Ghrl plasma levels in the fish groups at 12°C and 15°C, which displayed higher feed intake and gastrointestinal content relative to the fish maintained at 8°C. However, the Ghrl response to temperature and feed intake on Atlantic salmon are inconsistent. Similarly, Ghrl levels were found to be lower in Atlantic salmon at 19°C that showed lower feed intake, growth, and feed utilization compared a similar group at 14°C (Hevrøy et al., 2012). No changes in plasma Ghrl levels were observed in Atlantic salmon kept at either 8°C, 12°C or 18°C, despite a negative correlation between temperature and feed intake, FCR and growth (Kullgren et al., 2013). Vikeså et al. (2015) reported no changes in Ghrl plasma levels in fish with comparable feed intake and FCR at 13°C and 19°C. These discrepancies in Ghrl responses might be attributed to differences in feeding regimes, experimental condition, fish developmental stage and sampling time. In addition, ghrelin’s role in hunger regulation in Atlantic salmon may not strictly coincide with meal onset, but its action may be more closely associated with the fish energy balance, highlighting a complex interaction between hormonal regulation and energy homeostasis. Indeed, in a recent study, Ghrl action was linked to stimulation of lipid accumulation as energy storage, where a downregulation of plasma Ghrl levels was observed in Atlantic salmon exposed to an intermittent fasting regime for over 4 weeks (Lai et al., 2025).

### Stomach mRNA expression levels

A general concave-shaped response was observed in the mRNA levels of *ghrl1*, *ghrl2*, and *mboat,* the enzyme responsible for ghrelin activation. Transcript abundance decreased immediately after feeding and gradually returned towards initial levels by 24 hpf. An interaction between temperature and postprandial time was observed for *ghrl1*, with the lowest levels registered in the group exposed to 15°C. A temperature effect on ghrelin mRNA levels was observed in a previous study on Atlantic salmon, where lower levels of *ghrl1* were registered in fish kept at 19°C compared to a similar group at 14°C (Hevrøy et al., 2012). However, ghrelin mRNA levels were measured only four hpf, making direct comparison with our postprandial dynamics difficult. Most studies on ghrelin in Atlantic salmon have primarily focused on its response over days and weeks after feeding. For instance, *ghrl1* mRNA levels increased (Murashita et al., 2009), decreased (Hevrøy et al., 2011; Kalananthan et al., 2023) or showed no response (Del Vecchio et al., 2021) following fasting periods. Additional studies showed that postprandial expression of ghrelin in the stomach can vary significantly depending on the teleost species. A decrease in mRNA ghrelin was observed two hpf in Atlantic cod (*Gadus morhua*) (Xu and Volkoff, 2009), and 3h after feeding in goldfish (*Carassius auratus*) (Unniappan et al., 2004) and gibel carp (*C. auratus gibelio*) (Zhou et al., 2016). While, up and down postprandial dynamics were observed over 72hpf in the spotted sea bass (*Lateolabrax maculatus*) (Yu et al., 2020). In contrast, no postprandial changes were observed over 24 hpf for tilapia (*Oreochromis mossambicus*) (Fox et al., 2009).

Notably, our data showed a convex postprandial pattern of ghrelin mRNA expression levels, in contrast to the concave temporal dynamics observed for Ghrl in plasma. For instance, when plasma Ghrl is at its peak, ghrelin mRNA levels are at their lowest point, suggesting post-translational or post-transcriptional regulations, or regulatory mechanism wherein the expression of ghrelin is inhibited at elevated plasma ghrelin levels. Conversely, a decline in plasma ghrelin levels likely stimulates a rise in ghrelin mRNA expression to restore hormone levels. Similarly, Hevrøy and colleagues (2011) demonstrated an opposite response of ghrelin mRNA and Ghrl plasma levels post-feeding in Atlantic salmon. Contrary to these findings, a postprandial decrease in serum Ghrl was concomitant with a decrease in intestinal ghrelin mRNA levels in goldfish (Unniappan et al., 2004). While no changes were observed in both ghrelin mRNA and protein levels in tilapia (Fox et al., 2009). Taken together, the available data on ghrelin show that our understanding of ghrelin physiology in fish remains incomplete, highlighting the need for further functional studies. The many discrepancies in ghrelin responses (both mRNA and protein levels) to nutritional status, time, and environmental conditions highlight the complex regulatory mechanisms governing appetite and energy balance in teleosts. In particular, strong attention needs to be paid when comparing different studies, as variations in experimental conditions and fish status can lead to differing results. Further functional studies are required to elucidate the temporal and spatial dynamics of ghrelin expression and its interaction with other hormonal and metabolic pathways.

### Hypothalamic mRNA expression levels

While postprandial changes were evident in expression of all hypothalamic genes analysed, temperature effects was exclusively observed in the mRNA levels of *npya1*, *npya2*, *pomca1* and *pomca2*. Temperature had a negative effect on the expression dynamics of *npya* and *pomca* paralogues, with higher levels in fish at 8°C. Both *npya1* and *npya2* showed a significant increase post-feeding in all experimental groups, with peak levels registered at 24hpf, consistent with their orexigenic role related to meal anticipation in mammals and teleosts (Volkoff et al., 2005; Rønnestad et al., 2017). An exception was observed in fish at 8°C, where *npya1* showed a marginal decline at 24 hpf, but levels remained higher than at 0 hpf, still indicating a meal anticipation response but at diminished levels relative to the 12°C and 15°C groups and aligning with lower feed intake in this group. In contrast, *pomca1* and *pomca2* exhibited an increase in mRNA levels up to 12hpf, followed by a general drop down to 24 hpf in all groups, aligning with its anorexigenic role in the regulation of appetite (Volkoff et al., 2005; Rønnestad et al., 2017). Temperature was also found to affect the absolute abundance of the *npya* and *pomca* paralogues, where their expression higher at the lowest temperature at 8°C. These results also correspond with the absolute expression and postprandial dynamics of *ghrl1* in the stomach, where the lowest levels were registered in the group exposed to 15°C. The higher gene expression response observed at 8°C may indicate a metabolic adaptation in fish, where a higher mRNA response possibly is needed to enable reduced food intake in a voracious fish as salmonids, and sustain a lower metabolic rate and energy expenditure for food processing at low temperatures. No temperature effects were observed for *agrp1* and *cart2b,* but a post-feeding increase was noted in all experimental groups. However, the mRNA temporal changes for *agrp1* and *cart2b* were less pronounced compared to those for the *npya* and *pomca* paralogues, underscoring the major role of *npya* and *pomca* in regulating post-feeding satiety in this study.

### Maturation

The incidence of maturing fish was positively correlated with warmer temperatures. Early sexual development has become an increasing concern in Norwegian salmon farming, with elevated temperatures identified as one of the key triggering factors (Crouse et al., 2022; Pino Martinez et al., 2023; Pino-Martinez et al., 2024; Fraser et al., 2025). Although sexual maturation was not the main objective of this study, the data were utilized to explore possible correlations with hunger and appetite controlling mechanisms. The findings are consistent with existing literature, indicating that maturing fish tend to be smaller and shorter than non-maturing counterparts, but exhibit a higher condition factor (Pino Martinez et al., 2023; Fraser et al., 2025). Maturing fish had lower content in the stomach, midgut and hindgut, indicating that they consumed less feed compared to non-maturing fish maintained at the same water temperature. The results are consistent with the physiological changes that occur during salmon maturation, which are characterized by reduced feed intake. This reduction is thought to be linked with allocation of endogenous factors such as fat reserves, gonad development, and overall condition factor (Kadri et al., 1995; Taranger et al., 2010; Hvas et al., 2024). Data on blood chemistry highlighted the metabolic adjustments typical of reproductive development, with lower levels of glucose and higher levels of triglycerides and cholesterol in maturing fish. Notably, lipids are the primary energy source in teleost, and triglycerides plays a key role in regulating the initiation of reproductive maturation (Jenkins et al., 2019; Fraser et al., 2025).

The data from this study showed no significant differences in the expression of *ghrl* and *mboat* genes between maturing and non-maturing fish. However, a downregulation of hypothalamic genes in the maturing fish was observed, which highlights adjustments in the hypothalamic appetite key factors during the maturing phase. Further studies with larger sample sizes are needed to validate these findings and provide a more comprehensive understanding of how the onset of maturation—a key life-history decision in the iteroparous salmon—is modulated through a complex interplay between environmental factors and physiological regulatory mechanisms.

## Conclusion

The observed temperature-dependent physiological responses highlight the intricate balance between environmental factors and physiological adaptations. Findings from the 8-weeks trial revealed that water rearing temperature significantly modulates fish feed intake, feed efficiency ratio, hunger regulatory mechanisms and allocation of energy resources for growth. This heightened metabolic activity at elevated temperatures underscores the adaptive physiological mechanisms that aid the Atlantic salmon in optimizing nutrient assimilation and energy expenditure in response to their thermal environment.

## Data Availability

The original contributions presented in the study are publicly available. This data can be found here: https://dataverse.harvard.edu/dataset.xhtml?persistentId=doi:10.7910/DVN/VBYSHX.

## Abbreviations

*agrp1, agouti-related protein 1*; cart2b, cocaine- and amphetamine-regulated transcript 2b; FCR, feed conversion rate; FI, feed intake; FIR, feed intake relative; GBI, gallbladder index; ghrl1, ghrelin 1; ghrl2, ghrelin 2; GIT, gastrointestinal tract; GSI, gonadosomatic index; HSI, hepatosomatic index; K, condition factor; mboat, membrane-bound O-acyltransferase 4; npya1, neuropeptide y a1; npya2, neuropeptide y a2; pomca1, proopiomelanocortin a1; pomca2, proopiomelanocortin a2; RGR, relative growth rate; SGR, specific growth rate.
