# Supplementary material for: Influence of water temperature on feed intake, appetite control, and energy allocation in Atlantic salmon (Salmo salar) post-smolt
Source: Front Physiol. 2025 Aug 13;16:1646208. doi: 10.3389/fphys.2025.1646208 (PMC12380893; doi:10.3389/fphys.2025.1646208)
Supplement: Supplementary file 1 [file DataSheet1.pdf]

Supplementary materials to

Influence of water temperature on feed intake, appetite control, and energy allocation in Atlantic salmon (*Salmo salar*) post-smolt

F. Lai, S. Budaev, I. Hundven, P. Balseiro, S. Handeland, I. Rønnestad

TABLE OF CONTENTS

Introduction....1
Statistical modelling....1
Notation and abbreviations....2
Environmental condition of the three experimental groups....3
qPCR primers....3
Eight weeks monitor study....4
Feed intake....4
Growth....9
Somatic indices....16
24-Hours monitor study....20
Gastrointestinal transit and gall bladder index. .20
Metabolites and ghrelin plasma levels....25
Stomach mRNA expression levels....30
Hypothalamic mRNA expression levels....33
Maturation....39
General information....39
Growth and somatic indices....40
Gastrointestinal transit and gall bladder index. .42
Metabolites and ghrelin plasma levels....44
Stomach mRNA expression levels....46
Hypothalamic mRNA expression levels....46
References....48

INTRODUCTION

Statistical modelling

Model selection. For statistical analyses and graphics, we used R (R Core Team, 2025). A multi-model inference approach was used for the analysis of the data (Burnham and Anderson, 2002; Claeskens and Hjort, 2008). First, we fitted a series of models including a range of predictor variables. Second, we calculated the second order Akaike information criterion (AIC_c) assuming that the best inference model minimizes AIC_c. Third, based on ranked AIC_c, we calculated the Akaike evidence weights (w) for each individual ranked model. Finally, the model with the highest evidence weight (i.e. the lowest AIC_c) was considered the “best” within the family. When the best fitting model was determined, individual model parameter coefficients and effect size (Cohen’s *d*) values were analysed to understand the major effects. We also tested the hypotheses that the model parameters significantly differ from zero using t test.

Maturation effect analysis. A similar model comparison approach was used for the maturation analysis. To determine if maturation affect a response variable, we considered the best fitting model the baseline. Then, an additive maturation parameter was added to the model. Finally, we calculated the AICc and the evidence weights for the baseline and the maturation model. Additionally, we used ANOVA to determine if addition of the maturation indicator significantly improved fit.

Model fitting. The linear models were fitted using standard or generalized least squares depending on the residual error structure. When significant serial correlation of residuals was detected

(violating the assumption of independence for errors) a generalized least squares based on restricted maximum likelihood was used, adding an n-order autoregressive ARMA(n) process (Faraway, 2009; Fox, 2016). The optimal n was determined by fitting models with a range of ns and selecting the model characterized by lowest AICc as well as by comparing incremental models using analysis of variance (Chambers and Hastie, 1997; Fox, 2016). This was computed using the R 'gls' function from the 'nlme' package. For nonlinear estimation, we used standard 'nls' function. Model comparison based on Akaike evidence weights was done using the 'MuMIn'.

Data transformation. When the variables showed high skewness or significantly deviated from normality and the residual analysis revealed poor behaviour, we used square root or *log* transformation. In the case of GSI (where skewness was too high) we had to use the Box-Cox transformation $(x^{\lambda}-1)/\lambda$ (Faraway, 2009).

Notation and abbreviations

For linear models, we use the brief R like notation for formulas (Chambers and Hastie, 1997): "+" sign denoting additive effects and "×", both additive and interaction effects, ":" interaction and "/" nested effect. To denote a polynomial effect, we use an exponential notation where the exponent points to the polynom degree, e.g. ($\boldsymbol{D}^2$) refers to the second order polynomial model $\beta_1 D+\beta_2 D^2$. To distinguish the R notation from regular notation for equations, we use bold typeface variables in the former. For example, the R-like formula ***Y***=***A***+***B*** expands to the following equation $Y=\beta_0+\beta_1 A+\beta_2 B$, whereas ***Y***=***A***×***B*** expands to $Y=\beta_0+\beta_1 A+\beta_2 B+\beta_3 A B$, where β denote the fitted model coefficients as numbered by the respective index, Y denotes the independent variables, A and B are the predictors. Nested effects were denoted like ***T***/***t***, meaning that t is nested in T.

Example: the following equation ***FI*** = ***D*** × ***T*,** feed intake model in our brief R-like notation, translates to the following model $FI=\beta_0+\beta_1 D+\beta_2 T+\beta_3 D T$ that is fit using the following R basic linear model code: "`FI ~ lm(FI = D * T, data.frame)`".

Abbreviations used for independent variables

Variable	Description
T	Temperature, groups 8°, 12°, 15°C
D	Time, day
E	Time, week
H	Time post-meal, hours
St	Post-stress, defined as next day after sampling
S	Sex: 0 female, 1 male
t	Fish tank, used in some models as a nested factor: tank within temperature group
M	Maturation status 0=immature, 1= maturing male (GSI>0.06%, see Pino Martinez et al., 2023, 2021).

Environmental condition of the three experimental groups.

Table S1. Tank number and water conditions of the experimental groups reared at either 8˚C, 12˚C or 15˚C for 8 weeks.

	Environmental condition		
	**Group 8˚C**	**Group 12˚C**	**Group 15˚C**
**Tanks**	4, 6, 7	8, 9, 10	1, 2, 3
**Temperature**	8.40 ± 0.00	12.33 ± 0.00	14.84 ± 0.01
**O_2 %**	93.12 ± 0.05	94.94 ± 0.08	93.46 ± 0.12
**Salinity ‰**	28.50 ± 0.29	28.64 ± 0.22	28.26 ± 0.22

qPCR primers

Table S2. Primers sequences used for qPCR mRNA expression in Atlantic salmon. Amplicon sizes, qPCR efficiency and R2 are listed for each primer pair. F=forward; R=reverse.

Gene	GenBank ID	Primer Sequence (5' → 3')	Amplicon (bp)	Efficiency (%)	R^2	Reference
ghrl-I	NM_001142709.1	F: CCAGAAACCACAGGTAAGACAGGGTA<br>R: GAGCCTTGATTGTATTGTGTTTGTCT	128	89 %	0.99	Del Vecchio et al., 2021
ghrl-II	NM_001139585.1	F: TCCCAGAAACCACAGGGTAAA<br>R: GAGCCTTGATTGTATTGTGTTTGTCT	121	95 %	0.99	Del Vecchio et al., 2021
mboat4	XM_045703012.1	F: GGGTTGGCAAACATTCTGGC<br>R: ACACTGATAGGAGAAGCCTGG	89	97 %	1.00	Kalananthan et al., 2022
npya1	NM_001146681.1	F:GAACGCACAGCAGCAGAAAG<br>R:AGGATGCATATTGACTTGAAGGTT	80	103 %	1.00	Tolås et al., 2021
npya2	XM_014178359.1	F:CAGTCCAGGTATGATGAACCGT<br>R: GGCACAGGAGTAACCTCTGG	195	90 %	0.98	Tolås et al., 2021
agrp1	NM_001146677.1	F: ATGGTCATCTCAGTATTCCCAT<br>R: AGAGAGCCTTTACCGATATCTG	152	96 %	1.00	Kalananthan et al., 2020
pomca1	NM_001198575.1	F: ATACTTTTGAAACAGCGTGACGA<br>R: CAACGAGGATTCTCCCAGCA	108	94 %	1.00	Kalananthan et al., 2020
pomca2	NM_001198576.1	F: TTTGGCGACAGGCGAAGATG<br>R: TCCCAGCACTGACCTTTCAC	91	93 %	1.00	Kalananthan et al., 2020
cart2b	XM_014183838.2<br>NM_001146680.1	F: TGAGAGACTTCTACCCCAAAGA<br>R: CGTAGGGACTTGGCCGAATT	134	90 %	0.98	Kalananthan et al., 2021

EIGHT WEEKS MONITOR STUDY

Feed intake

Absolute Feed intake: FI

Table S3. Analysis of feed intake (*FI*) was based on selecting the best inference model among the following family of models. To account for serial correlations, each model was fitted using generalized least squares with autoregressive component with lag = 4

No	Model	AIC_c	w
12 10T	$FI = (D^3) \times T \times St + T/t$	**3449.7**	**0.98**
8 10	$FI = (D^3) \times T \times St$	3457.9	0.02
9 13	$FI = (D^3) + T + St + (D^3) : T + T : St + (D^3) : St$	3495.9	0.00
3 1	$FI = (D^2) \times T \times St$	3511.4	0.00
10 14	$FI = (D^3) + T + St + (D^2) : T + T : St + (D^2) : St$	3516.1	0.00
11 18T	$FI = T + St + (D^3) : T + T : St + (D^3) : St + T/t$	3518.2	0.00
10T2	$FI = (D^2) \times (T^2) \times St + T/t$	3518.4	0.00
5 3	$FI = (D^2) + T + T : St + (D^2) : St$	3525.6	0.00
X 18	$FI = T + St + (D^3) : T + T : St + (D^3) : St$	3528.2	0.00
6 6	$FI = (D^2) + T + St + (D^2) : T + T : St$	3543.5	0.00
7 7	$FI = (D^2) + (D^2) : T + T : St$	3558.9	0.00
11 15	$FI = (D^3) \times T$	3561.7	0.00
4 2	$FI = (D^2) + T + St + D : T + T : St$	3562.5	0.00
2 0	$FI = D \times T \times St$	3618.9	0.00
1 00	$FI = D \times T$	3645.9	0.00

Table S4. Beta coefficients for the best model

Effect		Beta±SE	t	p
Intercept		87.5±15.8	5.52	0.0000
D		20.3±236.4	0.08	0.9316
D^2		7.1±182.0	0.04	0.9688
D^3		120.1±146.9	0.82	0.4143
T		6.5±1.2	5.50	0.0000
St		1834.9±685.3	2.68	0.0077
$D : T$		70.7±19.7	3.59	0.0004
$D^2 : T$		27.0±15.3	1.77	0.0776
$D^3 : T$		-16.0±12.3	-1.30	0.1948
$D : St$		98187.8±36627.5	2.68	0.0076
$D^2 : St$		48802.5±18006.7	2.71	0.0070
$D^3 : St$		77991.6±29079.9	2.69	0.0076
$T : St$		-150.4±54.8	-2.80	0.0063
$T : t$		0.4±0.1	4.59	0.0000
$D : T : St$		-7708.4±2830.0	-2.73	0.0067
$D^2 : T : St$		-3951.6±1433.9	-2.76	0.0061
$D^3 : T : St$		-6072.1±2229.4	-2.73	0.0067

ANOVA effects for the best model. Overall ANOVA is presented here because the effect sizes cannot be determined form GLS model with autoregressive component.

Effect	df	F	p
$(\boldsymbol{D}^3)$	3	99.63	<0.0001
T	1	34.94	<0.0001
St	1	34.32	<0.0001
$(\boldsymbol{D}^3)$:***T***	3	5.56	0.0010
$(\boldsymbol{D}^3)$:***St***	3	0.28	0.8428
T:***St***	1	0.03	0.8528
T:***t***	1	21.65	<0.0001
T:$(\boldsymbol{D}^3)$:***St***	3	3.01	0.0301
Error	406		

Table S5. Identification of the best lag for autoregressive component in the best model (Table S4). To identify the lag, models were fitted with lags from 2 to 6. Then, the fit of the consecutive models was compared using AIC_c and ANOVA

Lags	df	AIC_c	Likelihood ratio	p
2 vs 3	20, 21	3456.76 vs 3450.98	7.99	0.0047
3 vs 4	21, 22	3450.98 vs **3449.70**	3.50	0.0612
4 vs 5	22, 23	**3449.70** vs 3451.59	0.34	0.5584
5 vs 6	23, 24	3451.59 vs 3453.34	0.51	0.4772

Note: Autoregressive lag = 4 was selected based on minimum AIC_c.

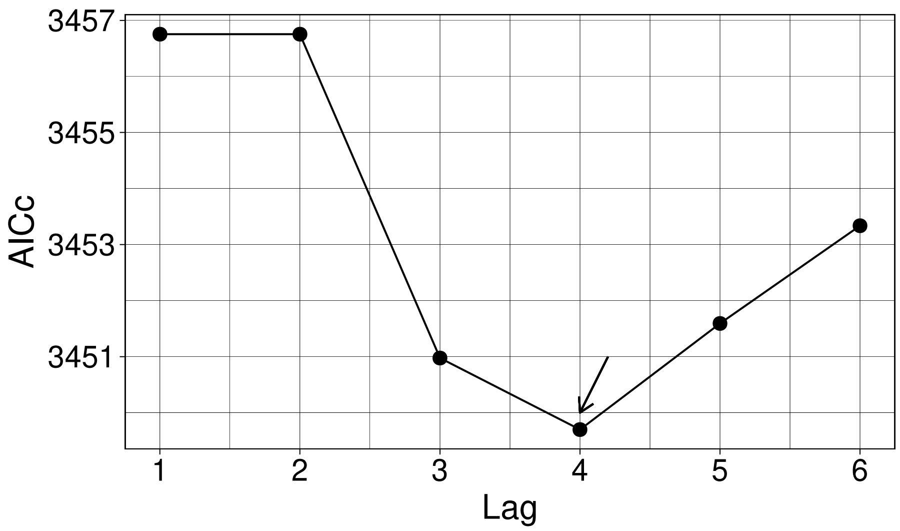

Figure S1. *AICc for the different ARMA lags in Table S5. Arrow points to the best lag*

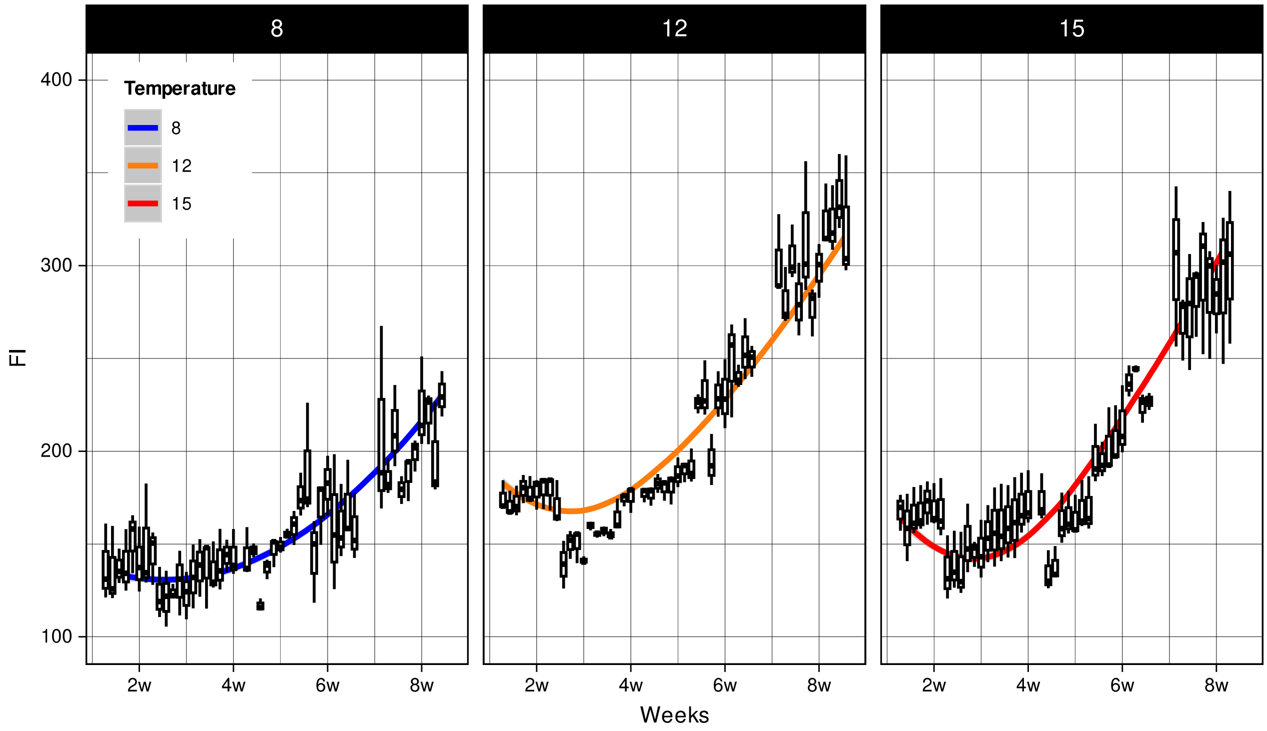

Figure S2. *Feed intake FI over the days of the trial*

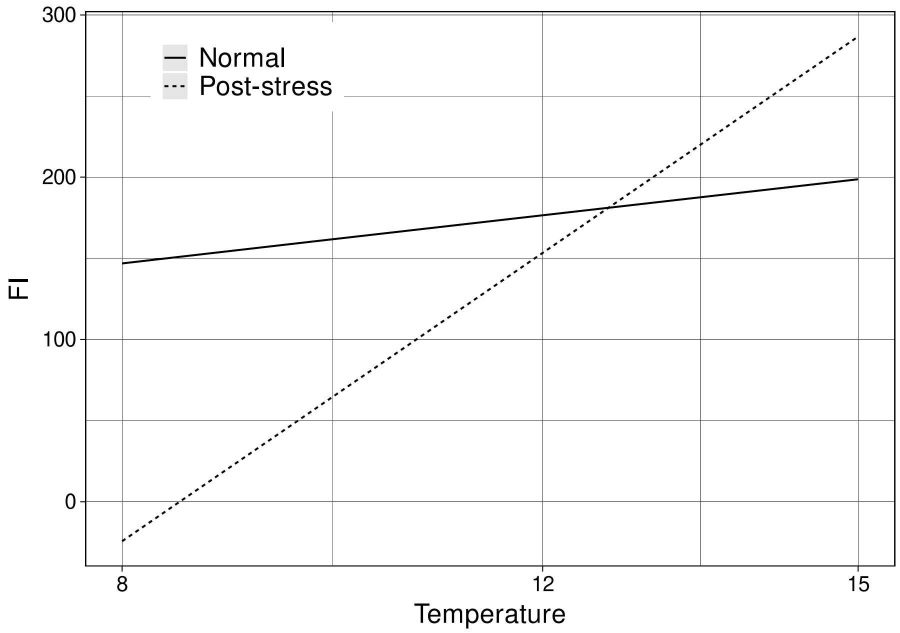

Figure S3. *Predicted effect plot for the* ***T*** × ***St*** *interaction, see Table S4*

Relative Feed intake: FIR

Table S6. Analysis of relative feed intake (*FIR*) was based on selecting the best inference model among the following family of models. To account for serial correlations, each model was fitted using generalized least squares with autoregressive component with lag = 3

No	Model	AIC_c	w
21	$FIR = (D^2) + T + St$	-1239.8	**0.970**
14 19	$FIR = (D^2) + T : St$	-1232.5	0.025
8 7	$FIR = (D^2) + (D^2) : T + T : St$	-1228.9	0.004
13 18	$FIR = (D^3) + (D^2) : T + T : St$	-1224.9	0.001
7 6	$FIR = (D^2) + T + St + (D^2) : T + T : St$	-1218.8	0.000
9 10	$FIR = (D^3) \times T \times St$	-1217.8	0.000
5 3	$FIR = (D^2) + T + St + (D^2) : T + T : St + (D^2) : St$	-1216.6	0.000
12 17	$FIR = (D^3) + T + St + (D^2) : T + T : St$	-1214.7	0.000
10 13	$FIR = (D^3) + T + St + (D^2) : T + T : St + (D^3) : St$	-1214.5	0.000
11 16	$FIR = (D^3) + T + St + (D^3) : T + T : St$	-1212.9	0.000
4 2	$FIR = (D^2) + T + St + D : T + T : St$	-1212.5	0.000
6 5	$FIR = (D^2) + T + St + D : T + T : St$	-1212.5	0.000
16 20	$FIR = (D^2)$	-1211.0	0.000
3 1	$FIR = (D^2) \times T \times St$	-1208.4	0.000
15 19T	$FIR = (D^2) + (D^2) : T + T/t$	-1181.1	0.000
1 00	$FIR = D \times T$	-1165.8	0.000
2 0	$FIR = D \times T \times St$	-1162.5	0.000

Table S7. Beta coefficients for the best model

Effect		Beta±SE	t	p
Intercept		0.43 ± 0.07	6.23	0.0000
D		-0.78 ± 0.25	-3.17	0.0017
D^2		0.80 ± 0.19	4.25	0.0000
T		0.01 ± 0.01	2.27	0.0237
St		-0.06 ± 0.01	-6.84	0.0000

ANOVA effects for the best model. Overall ANOVA is presented here because the effect sizes cannot be determined form GLS model with autoregressive component.

Effect	df	F	p
(D^2)	2	14.85	<0.0001
T	1	5.24	0.0225
St	1	46.84	<0.0001
Error	418		

Table S8. Identification of the best lag for autoregressive component in the best model (Table S7). To identify the lag, models of the form 12 were fitted with lags from 2 to 5. Then, the fit of the consecutive models was compared using AIC_c and ANOVA

**Lags**	**df**	**AIC_c**	**Likelihood ratio**	**p**
2 vs 3	8, 9	-1232.12 vs **-1239.84**	9.80	0.0017
3 vs 4	9, 10	**-1239.84** vs -1240.24	2.50	0.1139
4 vs 5	10, 11	-1240.24 vs -1238.23	0.09	0.7551

Note: Autoregressive lag = 3 was selected, based on the major breakpoint of AIC_c (see Figure S4).

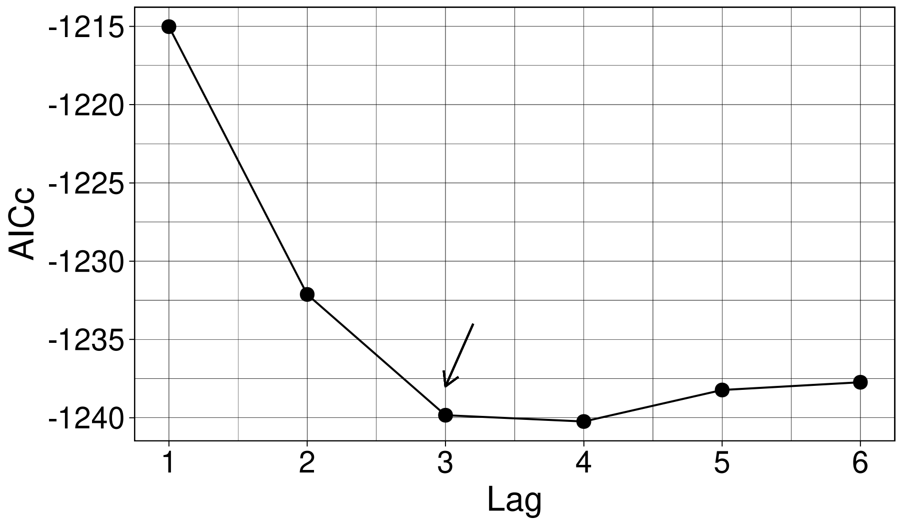

Figure S4. *AICc for the different ARMA lags in Table S8. Arrow points to the best lag*

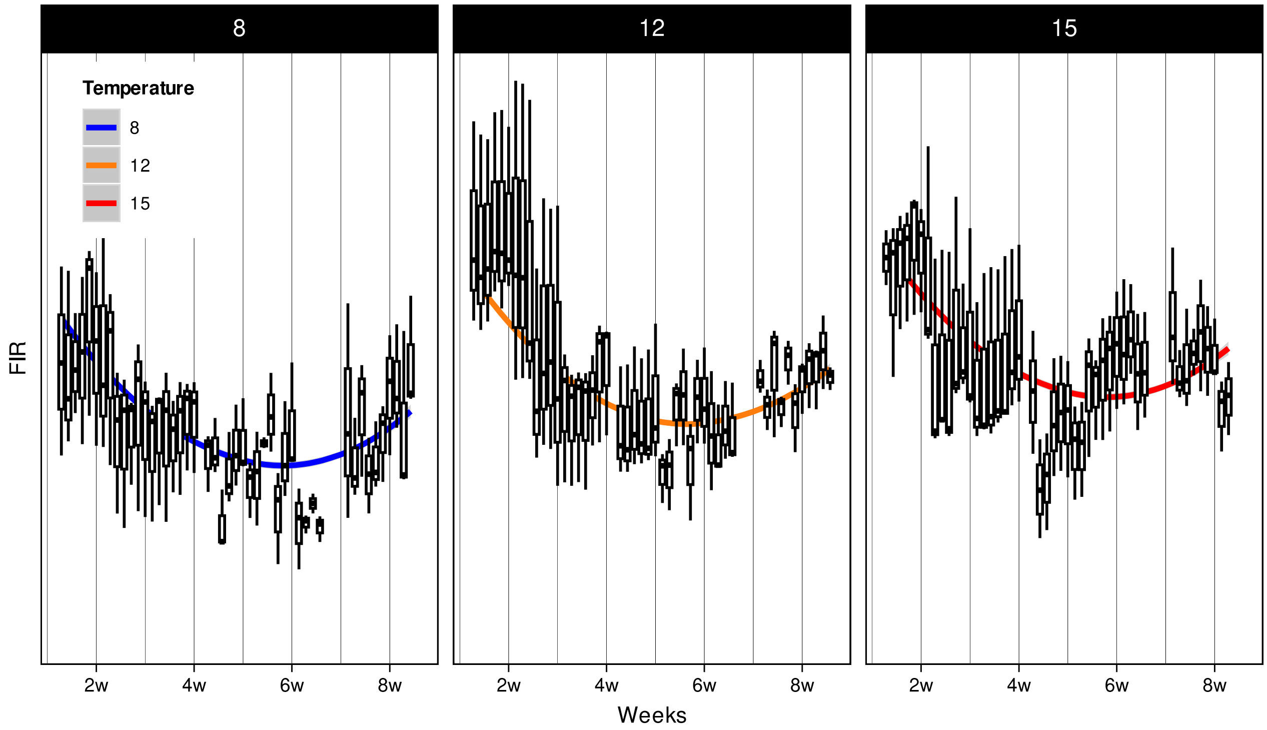

Figure S5. *Relative feed intake FIR over the days of the trial*

Growth

Body weight

Table S9. Analysis of body weight (*W*) was based on selecting the best inference model among the following family of models. No serial correlation was observed (p<0.05)

No	Model	AIC_c	w
4 4	$\sqrt{W} = (E^3) + T$	2031.5	**0.280**
3 3	$\sqrt{W} = E + T$	2032.0	0.214
2 2	$\sqrt{W} = E \times T$	2032.1	0.202
7 8	$\sqrt{W} = (E^4) + T$	2033.3	0.109
6 7	$\sqrt{W} = (E^2) + T$	2033.5	0.099
1 0	$\sqrt{W} = E$	2034.7	0.054
5 5	$\sqrt{W} = (E^3) \times T$	2035.2	0.043

Table S10. Beta coefficients for the best model

Effect	Effect size	Beta±SE	t	p
Intercept		17.77 ± 0.69	25.833	<0.0001
E	12.72	54.33 ± 3.24	16.757	<0.0001
E^2	0.54	2.29 ± 3.24	0.707	0.4798
E^3	-1.54	-6.58 ± 3.24	-2.029	0.0432
T	0.08	0.13 ± 0.06	2.196	0.0287

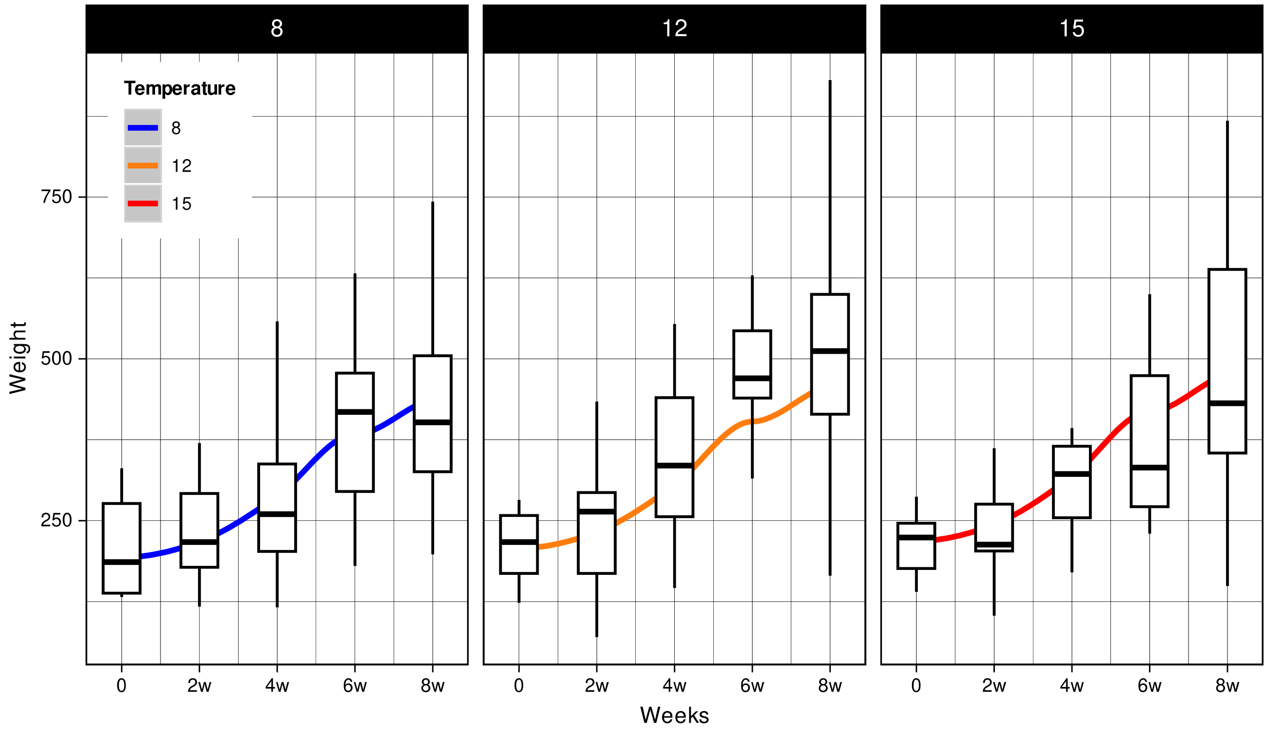

Figure S6. *Body weight W over the weeks of the trial*

Body length

Table S11. Analysis of body length (*L*) was based on selecting the best inference model among the following family of models. No serial correlation was observed (p<0.05)

No	Model	AIC_c	w
2 0	$\sqrt{L}=E$	177.9	**0.291**
3 2	$\sqrt{L}=E\times T$	178.5	0.216
4 4	$\sqrt{L}=E+T$	178.8	0.190
6 7	$\sqrt{L}=(E^3)+T$	178.9	0.177
7 9	$\sqrt{L}=(E^2)+T$	180.7	0.071
8 10	$\sqrt{L}=(E^2)\times T$	182.5	0.029
5 5	$\sqrt{L}=(E^3)\times T$	182.8	0.026
1 00	$\sqrt{L}=c$ (constant)	383.0	0.000

Table S12. Beta coefficients for the best model

Effect	Effect size	Beta±SE	t	p
Intercept		5.00 ± 0.034	149.09	<0.0001
E	0.64	0.09 ± 0.01	16.49	<0.0001

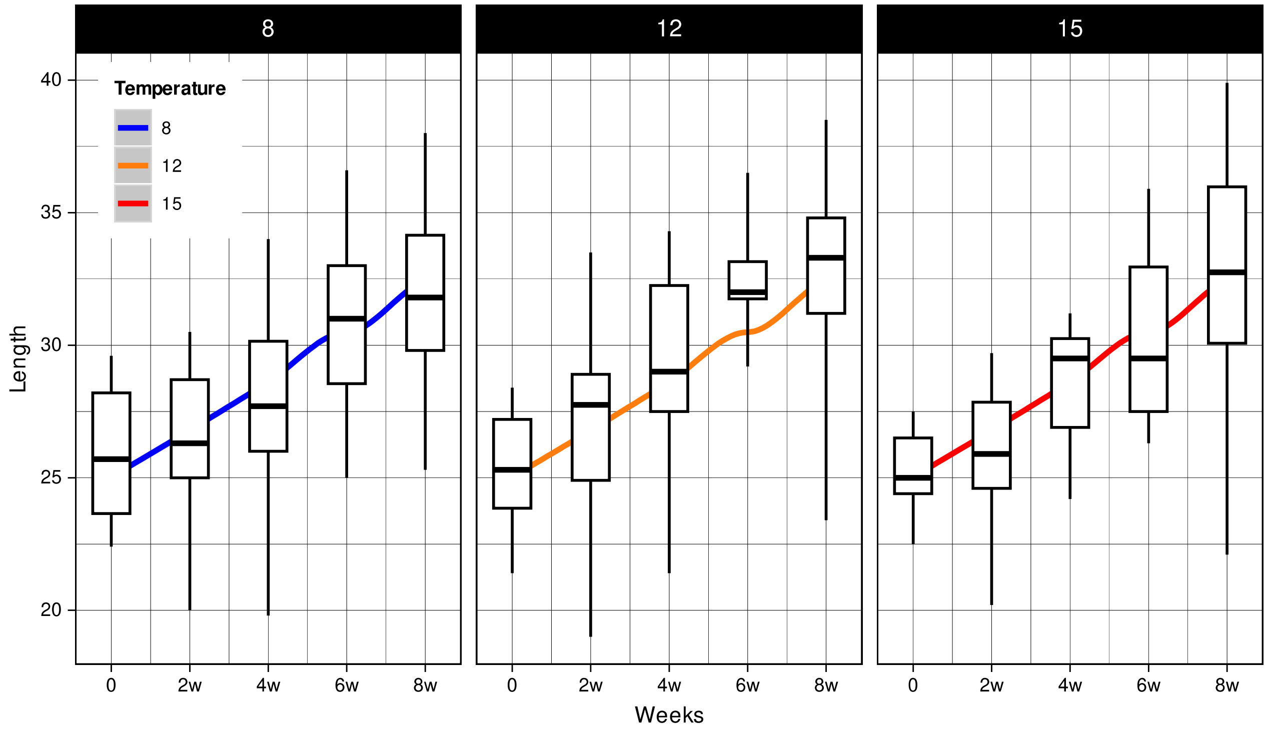

Figure S7. *Body length L over the weeks of the trial*

K factor

Table S13. Analysis of K factor was based on selecting the best inference model among the following family of models. No serial correlation was observed (p<0.05)

No	Model	AIC_c	w
1 7a	$\boldsymbol{K = E + T + E{:}(T^2)}$	-613.4	**0.626**
1 7	$\boldsymbol{K = E \times (T^2)}$	-611.4	0.223
2 9	$\boldsymbol{K = E + (T^2)}$	-609.3	0.081
3 11	$\boldsymbol{K = (E^2) \times (T^2)}$	-608.8	0.063
4 8	$\boldsymbol{K = E \times (T^2) + T/t}$	-602.2	0.002
5 10	$\boldsymbol{K = E + (T^2) + T/t}$	-600.8	0.001
10 3	$\boldsymbol{K = E + T + T/t}$	-600.8	0.001
8 1	$\boldsymbol{K = E \times T + T/t}$	-600.8	0.001
9 2	$\boldsymbol{K = E \times T}$	-598.8	0.000
11 4	$\boldsymbol{K = E + T}$	-598.5	0.000
12 5	$\boldsymbol{K = (E^2) \times T + T/t}$	-598.3	0.000
13 6	$\boldsymbol{K = (E^2) \times T}$	-596.3	0.000
7 0a	$\boldsymbol{K = E}$	-584.1	0.000
6 0	$\boldsymbol{K = T}$	-569.8	0.000

Table S14. Beta coefficients for the best model

Effect	Effect size	Beta±SE	t	p
Intercept		1.08 ± 0.05	21.239	< 0.0001
E	0.27	0.01 ± 0.001	5.761	< 0.0001
T	0.20	0.01 ± 0.004	3.275	0.00115
$\boldsymbol{E : T}$	-1.43	-0.06 ± 0.04	-1.541	0.12417
$\boldsymbol{E : T^2}$	-1.79	-0.07 ± 0.02	-4.104	< 0.0001

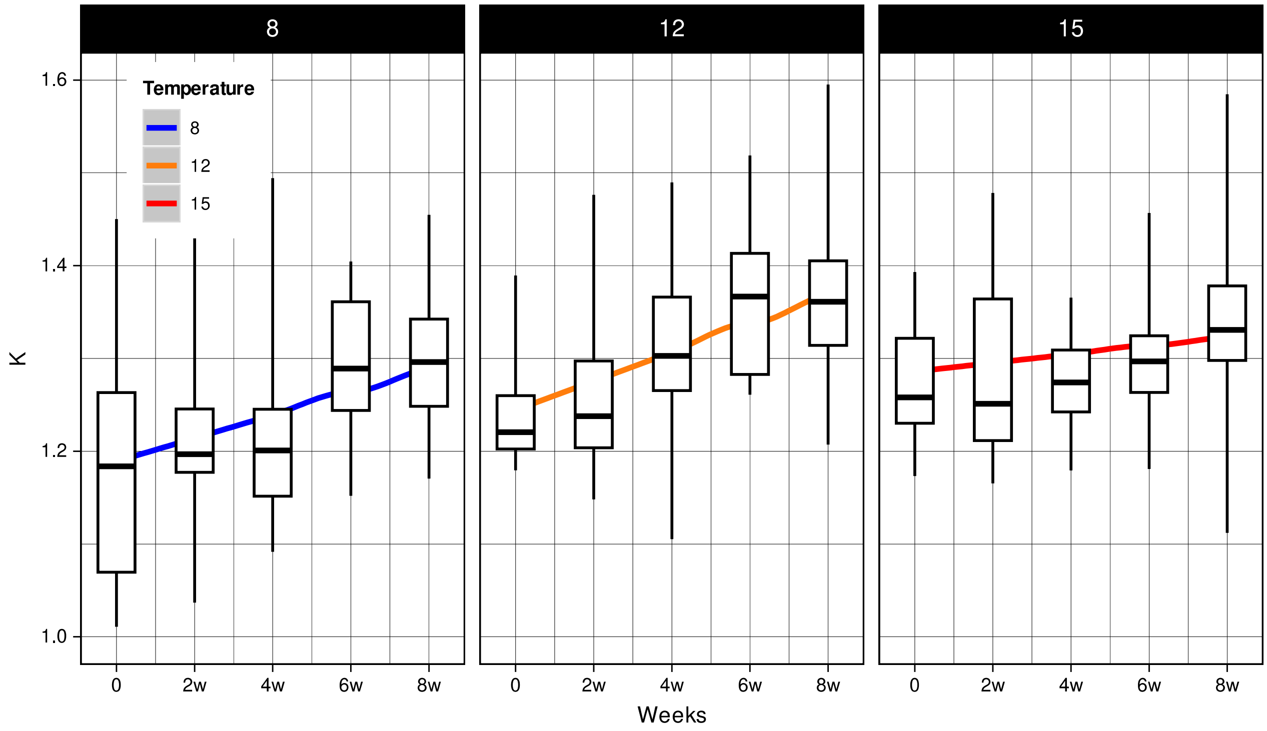

Figure S8. *K factor over the weeks of the trial*

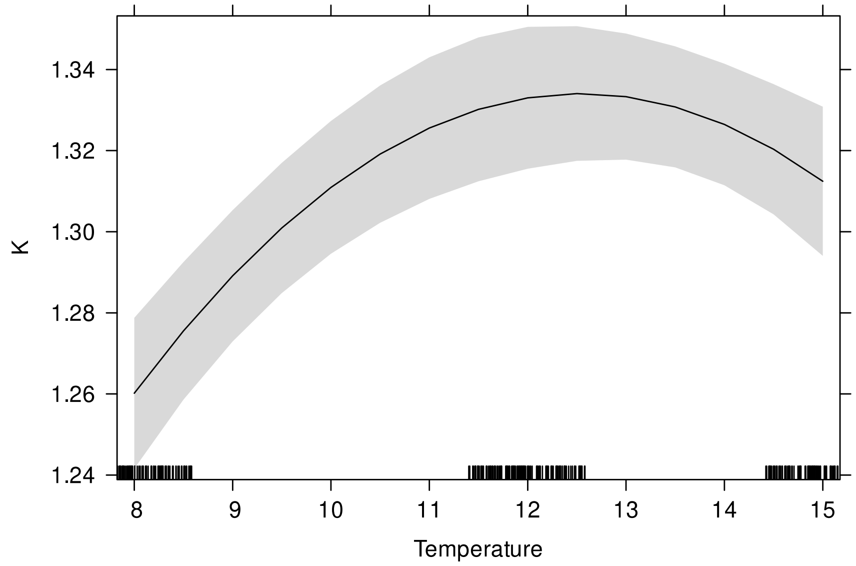

Figure S9. *Predicted temperature effect plot for K factor*

SGR

Table S15. Analysis of SGR was based on selecting the best inference model among the following family of models. No effect of time can be meaningfully determined because SGR was determined between the start and the end of the trial. The effect of two groups (**G**) were also determined. No serial correlation was observed (p<0.05)

No	Model	AIC_c	w
15	$SGR = (T^2) + G + T/t_0 + T/t_1$	-133.5	**0.359**
2	$SGR = T + G + T/t_0 + T/t_1$	-133.5	**0.359**
1	$SGR = T + T/t_0 + T/t_1$	-130.4	0.076
17	$SGR = (T^2) + T/t_0 + T/t_1$	-130.4	0.076
16	$SGR = (T^2) \times G + T/t_0 + T/t_1$	-130.3	0.074
7	$SGR = T + G + T/t_0$	-127.4	0.017
6	$SGR = T + T/t_0$	-127.3	0.017
18	$SGR = (T^2) + T/t_0$	-127.3	0.017
5	$SGR = T + G + T/t_1$	-121.9	0.001
10	$SGR = (T^2) + G$	-121.9	0.001
3	$SGR = T + G + T/t_0 + T/t_1$	-121.1	0.001
4	$SGR = T + T/t_1$	-119.9	0.000
13	$SGR = (T^2) + (T^2)/t_1$	-119.9	0.000
9	$SGR = (T^2) + T/t_1$	-119.9	0.000
11	$SGR = (T^2) \times G + T/t_1$	-118.6	0.000
12	$SGR = (T^2)$	-116.5	0.000
8	$SGR = T$	-0.6	0.000
14	$SGR = T + G$	0.6	0.000

Table S16. Beta coefficients for the best model

Effect	Effect size	Beta±SE	t	p
Intercept		1.498±0.070	21.437	< 0.0001
T	24.48	-1.220±1.678	-0.727	0.468
T^2	-21.12	-2.150±0.858	-2.504	0.013
G	0.17	0.048±0.021	2.258	0.025
(beta parameters and effects for specific tanks not shown to save space because their exact values are of minor importance)				

ANOVA effects for the best model. Overall ANOVA is presented here to show the overall effects of tanks because the beta parameters for individual tanks are excluded.

Effect	df	F	p
(T^2)	2	198.2791	< 0.0001
G	1	1.3357	0.249
$T : t_0$	7	3.9298	0.000
$T : t_1$	6	3.1220	0.005
Error	360		

Note to tables S15 and S16 : t_1 and t_2 refer to tank effects at the start and end

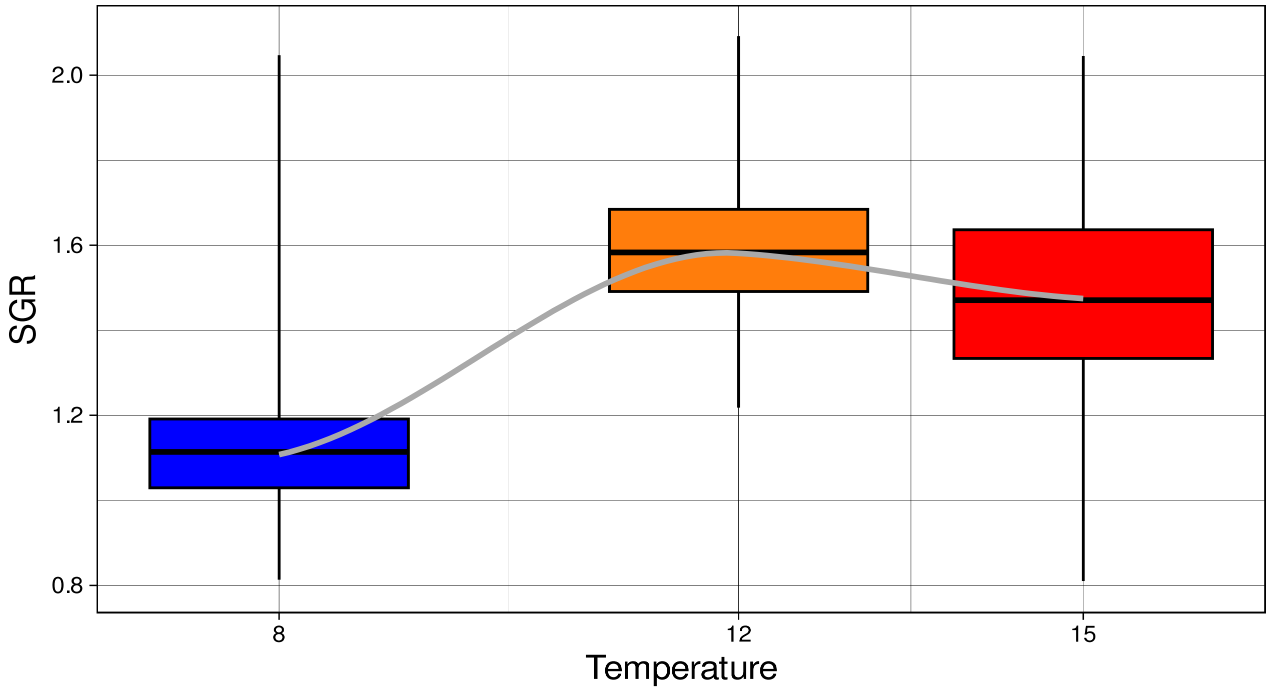

Figure S10. *Link between temperature and SGR*

RGR

Table S17. Analysis of RGR was based on selecting the best inference model among the following family of models. No effect of time can be meaningfully determined because RGR was determined between the start and the end of the trial. No serial correlation was observed (p<0.05)

No	Model	AIC_c	w
17	$RGR = (T^2) + T/t_0 + T/t_1$	3526.8	**0.354**
1	$RGR = T + T/t_0 + T/t_1$	3526.8	**0.354**
2	$RGR = T + G + T/t_0 + T/t_1$	3529.0	0.119
15	$RGR = (T^2) + G + T/t_0 + T/t_1$	3529.0	0.119
6	$RGR = T + T/t_0$	3533.0	0.016
18	$RGR = (T^2) + T/t_0$	3533.0	0.016
16	$RGR = (T^2) \times G + T/t_0 + T/t_1$	3533.2	0.014
7	$RGR = T + G + T/t_0$	3534.8	0.007
4	$RGR = T + T/t_1$	3539.2	0.001
9	$RGR = (T^2) + T/t_1$	3539.2	0.001
13	$RGR = (T^2) + (T^2)/t_1$	3539.2	0.001
5	$RGR = T + G + T/t_1$	3541.3	0.000
10	$RGR = (T^2) + G$	3541.3	0.000
3	$RGR = T + G + T/t_0 + T/t_1$	3542.9	0.000
11	$RGR = (T^2) \times G + T/t_1$	3545.4	0.000
12	$RGR = (T^2)$	3545.9	0.000
8	$RGR = T$	3664.9	0.000
14	$RGR = T + G$	3666.2	0.000

Table S18. Beta coefficients for the best model

**Effect**	**Effect size**	**Beta±SE**	**t**	**p**
Intercept		135.126±8.907	15.170	< 0.0001
T	31.16	-170.552±215.115	-0.793	0.4284
$\boldsymbol{T^2}$	-25.89	-316.296±110.339	-2.867	0.0044
(beta parameters and effects for specific tanks not shown to save space because their exact values are of minor importance)				

ANOVA effects for the best model. Overall ANOVA is presented here to show the overall effects of tanks because the beta parameters for individual tanks are excluded.

**Effect**	**df**	**F**	**p**
$(\boldsymbol{T^2})$	2	173.8787	< 0.0001
$\boldsymbol{T : t_0}$	7	4.1025	0.0002
$\boldsymbol{T : t_1}$	6	3.1470	0.0051
Error	361		

Note to tables S17 and S18 : $\boldsymbol{t_1}$ and $\boldsymbol{t_2}$ refer to tank effects at the start and end

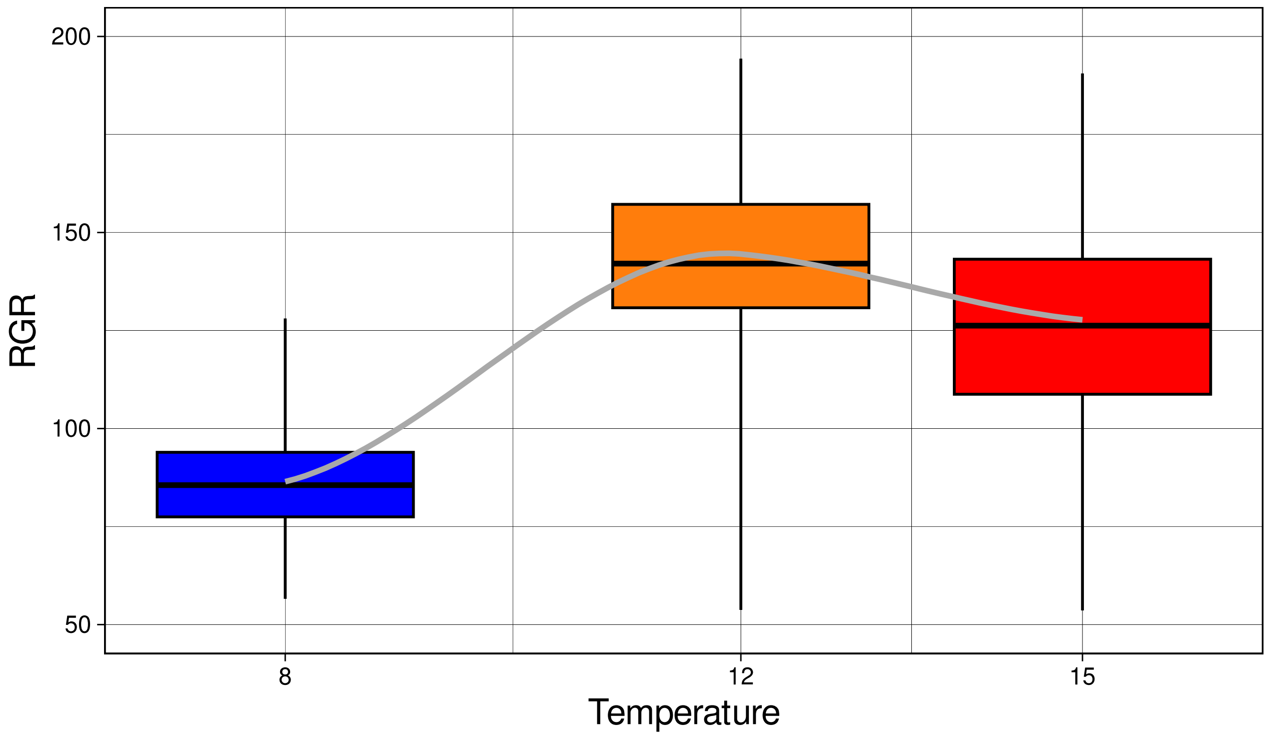

Figure S11. *Link between temperature and RGR*

FCR

Table S19. Average FCR for the three temperature groups

Temperature	Tank	Final gained Biomass	Feed eaten	FCR	Average FCR
8°C	4	7529	7058.54	0.93751	0.96
8°C	6	7913	7800.53	0.98579	
8°C	7	7705	7430.69	0.96440	
12°C	8	12754	10287.50	0.80661	0.77
12°C	9	13167	9879.98	0.75036	
12°C	10	14114	10546.36	0.74723	
15°C	1	10098	9204.86	0.91155	0.79
15°C	2	11605	8846.45	0.76230	
15°C	3	12638	8872.01	0.70201	

Somatic indices

HSI

Table S20. Analysis of HSI was based on selecting the best inference model among the following family of models. No serial correlation was observed (p<0.05)

No	Model	AIC_c	w
10	$HSI = (E^2) + T + T{:}E$	-428.1	**0.525**
7	$HSI = (E^2) \times T$	-427.0	0.293
9	$HSI = E + T + T{:}(E^2)$	-425.6	0.149
8	$HSI = (E^2) + T$	-422.5	0.032
2	$HSI = E \times T$	-415.4	0.001
3	$HSI = E + T$	-410.0	0.000
6	$HSI = (E^2)$	-407.9	000
1	$HSI = E \times T / t$	-406.8	000
4	$HSI = E + T + T / t$	-402.6	000
0a	$HSI = E$	-396.0	000
0	$HSI = T$	-380.0	000
5	$HSI = T + T / t$	-371.0	000

Table S21. Beta coefficients for the best model

Effect	Effect size	Beta±SE	t	p
Intercept		1.203 ± 0.029	40.94	< 0.0001
E	5.44	-0.739 ± 0.580	-1.27	0.203591
E^2	3.55	0.535 ± 0.138	3.86	0.000132
T	-0.19	-0.023 ± 0.001	-4.35	< 0.0001
$E : T^2$	0.13	0.002 ± 0.001	2.76	0.005983

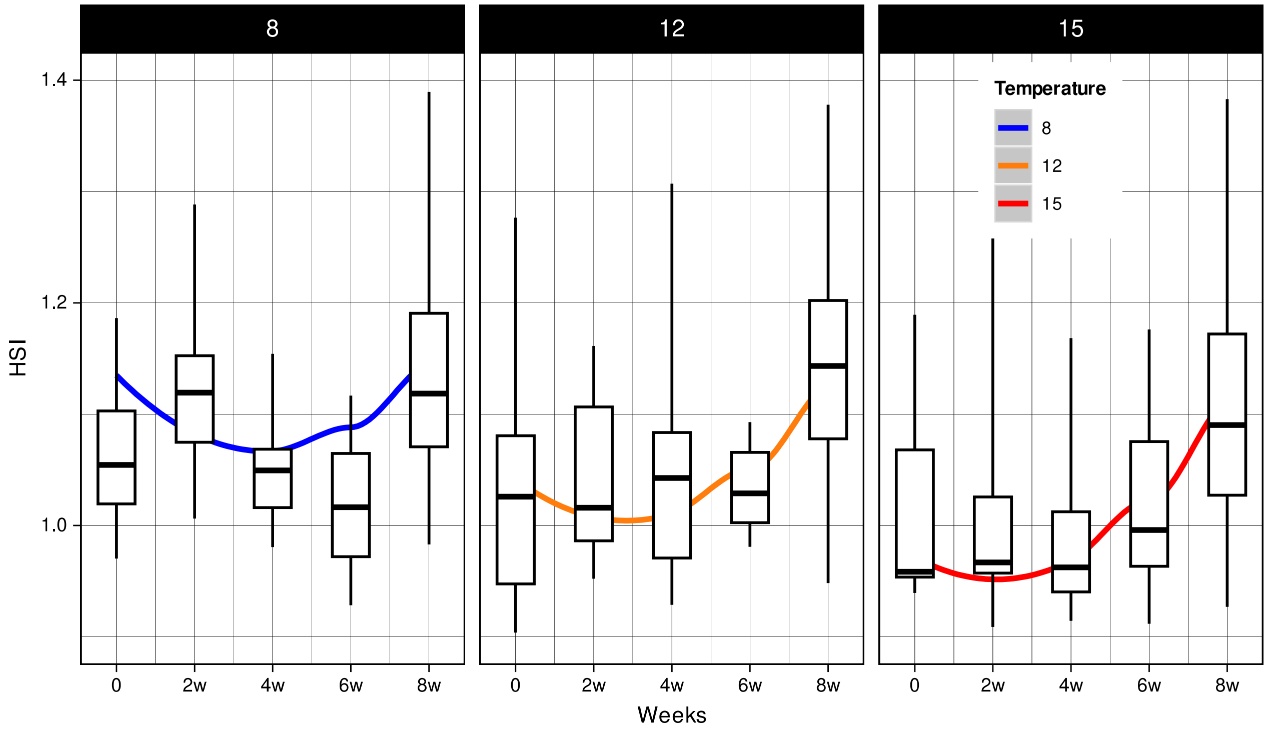

Figure S12. *HSI over the weeks of the trial*

GSI

Table S22. Analysis of *GSI* was based on selecting the best inference model among the following family of models. No serial correlation was observed (p<0.05)

No	Model	AIC$_c$	w
11	$\frac{GSI^{\lambda}-1}{\lambda}=\boldsymbol{S}+\boldsymbol{S}:\boldsymbol{E}:\boldsymbol{T}$	1920.6	**0.465**
18	$\frac{GSI^{\lambda}-1}{\lambda}=\boldsymbol{S}\times(\boldsymbol{E}^2)\times\boldsymbol{T}$	1920.9	0.392
15	$\frac{GSI^{\lambda}-1}{\lambda}=\boldsymbol{S}\times\boldsymbol{E}\times\boldsymbol{T}$	1924.3	0.072
19	$\frac{GSI^{\lambda}-1}{\lambda}=\boldsymbol{S}+\boldsymbol{S}:\boldsymbol{E}:\boldsymbol{T}+\boldsymbol{T}/\boldsymbol{t}$	1924.5	0.067
13	$\frac{GSI^{\lambda}-1}{\lambda}=\boldsymbol{S}+\boldsymbol{S}:\boldsymbol{E}$	1932.4	0.001
8	$\frac{GSI^{\lambda}-1}{\lambda}=\boldsymbol{S}+\boldsymbol{E}+\boldsymbol{T}$	1933.2	0.001
16	$\frac{GSI^{\lambda}-1}{\lambda}=\boldsymbol{S}+\boldsymbol{E}\times\boldsymbol{T}$	1933.4	0.001
17	$\frac{GSI^{\lambda}-1}{\lambda}=\boldsymbol{S}+\boldsymbol{E}+\boldsymbol{T}$	1939.3	0.000
9	$\frac{GSI^{\lambda}-1}{\lambda}=\boldsymbol{S}+\boldsymbol{E}$	1939.8	0.000

No	Model	AIC$_c$	w
14	$\frac{GSI^{\lambda}-1}{\lambda}=\boldsymbol{S}+\boldsymbol{S}:\boldsymbol{T}$	1944.8	0.000
10	$\frac{GSI^{\lambda}-1}{\lambda}=\boldsymbol{S}+\boldsymbol{T}$	1947.9	0.000
12	$\frac{GSI^{\lambda}-1}{\lambda}=\boldsymbol{S}$	1948.2	0.000
6	$\frac{GSI^{\lambda}-1}{\lambda}=\boldsymbol{E}:\boldsymbol{T}$	2140.6	0.000
3	$\frac{GSI^{\lambda}-1}{\lambda}=\boldsymbol{E}\times\boldsymbol{T}$	2142.8	0.000
4	$\frac{GSI^{\lambda}-1}{\lambda}=\boldsymbol{E}$	2142.9	0.000
2	$\frac{GSI^{\lambda}-1}{\lambda}=\boldsymbol{E}+\boldsymbol{T}$	2144.2	0.000
7	$\frac{GSI^{\lambda}-1}{\lambda}=\boldsymbol{E}:\boldsymbol{T}+\boldsymbol{T}/\boldsymbol{t}$	2144.5	0.000
1	$\frac{GSI^{\lambda}-1}{\lambda}=\boldsymbol{E}\times\boldsymbol{T}+\boldsymbol{T}/\boldsymbol{t}$	2144.6	0.000
5	$\frac{GSI^{\lambda}-1}{\lambda}=\boldsymbol{T}$	2145.2	0.000

Note: Box-Cox transformation parameter λ=-0.505 maximizes log-likelihood for the linear model.

Table S23. Beta coefficients for the best model

Effect	Effect size	Beta±SE	t	p
Intercept		-4.920 ± 0.386	-12.75	< 0.0001
S	-1.27	-6.839 ± 0.581	-11.77	< 0.0001
$\boldsymbol{S_f}$: ***E*** : ***T***	0.08	0.002 ± 0.005	0.48	0.631
$\boldsymbol{S_m}$: ***E*** : ***T***	0.15	0.031 ± 0.006	5.70	< 0.0001

ANOVA effects for the best model

Effect	df	F	p
S	1	276.926	<0.0001
S : E : T	2	16.342	<0.0001
Error	384		

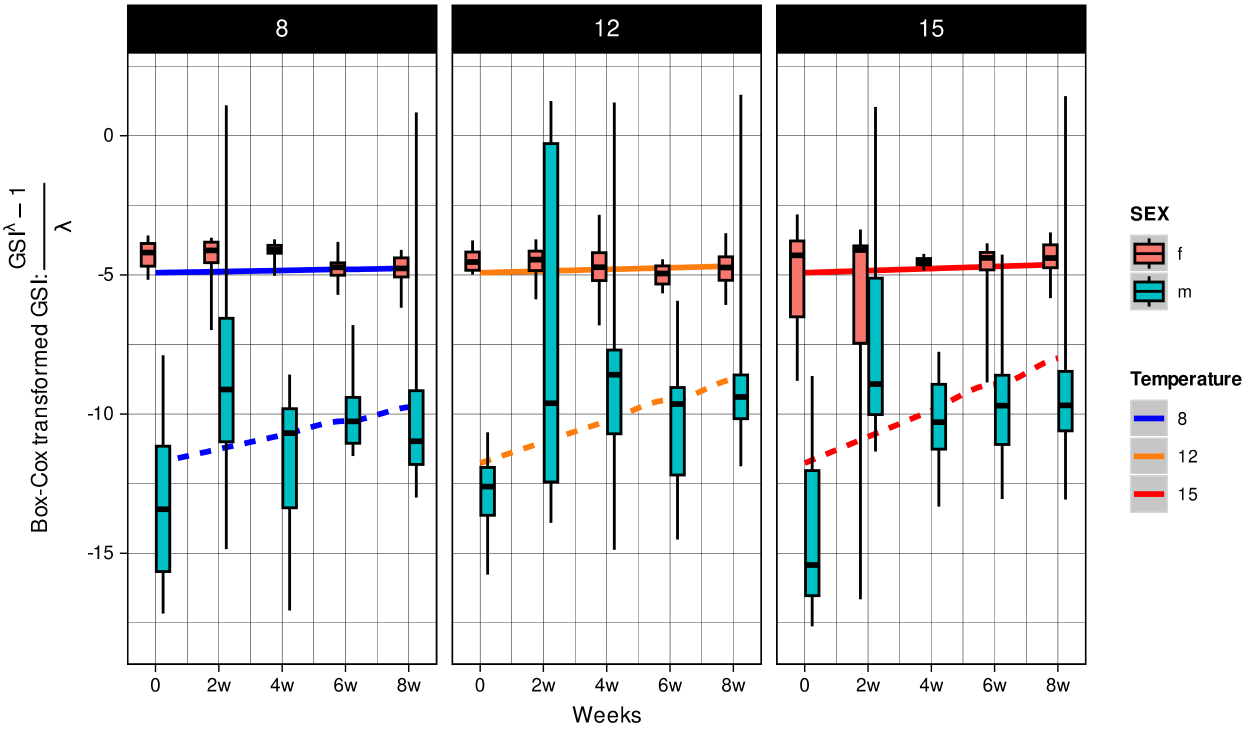

Figure S13. *GSI over the weeks of the trial*

24-HOURS MONITOR STUDY

Gastrointestinal transit and gall bladder index

Stomach

Table S24. Analysis of the relative stomach fullness (*STOM*) was based on selecting the best inference model among the following family of models

No	Model	AIC$_c$	w
5	$STOM=(a+b\times T+c\times H+d\times T\times H)^2$	-119.6	**0.632**
5s	$STOM=(a+b\times T+c\times H+d\times T\times H+s\times S)^2$	-117.5	0.224
4	$STOM=(a+b\times T+c\times H)^2$	-115.7	0.089
2	$STOM=y_f+(y_0-y_f)\mathrm{e}^{(-\alpha\times H+\beta\times T+\gamma\times H\times T)}$	-114.2	**0.042**
1	$STOM=y_f+(y_0-y_f)\mathrm{e}^{(-\alpha\times H+\beta\times T)}$	-111.3	0.010
0	$STOM=y_f+(y_0-y_f)\mathrm{e}^{(-\alpha\times H)}$	-109.2	0.003
3a	$STOM=y_0\mathrm{e}^{(-\alpha\times H+\beta\times T)}$	-101.1	0.000
3	$STOM=y_0\mathrm{e}^{(-\alpha\times H+\beta\times T+\gamma\times H\times T)}$	-100.4	0.000
3b	$STOM=(y_0+y_1\times T)\mathrm{e}^{(-\alpha\times H+\beta\times T)}$	-100.0	0.000

Table S25. Beta coefficients for the best model (quadratic)

Effect	Effect size	Beta±SE	t	p
a	-0.47	0.769 ± 0.057	13.54	<0.00001
b	-0.03	0.051 ± 0.005	3.16	0.00182
c	0.45	-0.018 ± 0.009	-2.15	0.03301
d	0.04	-0.002 ± 0.001	-2.45	0.01508

Table S26. Beta coefficients for the best fitting exponential decay model

Effect	Effect size	Beta±SE	t	p
y_0	-0.21	0.613 ± 0.085	7.23	<0.00001
y_f	-1.83	-0.301 ± 0.131	-2.30	0.02223
α	0.51	0.038 ± 0.015	2.48	0.01392
β	-0.01	0.022 ± 0.008	2.76	0.00634
γ	-0.05	-0.002 ± 0.001	-2.11	0.03617

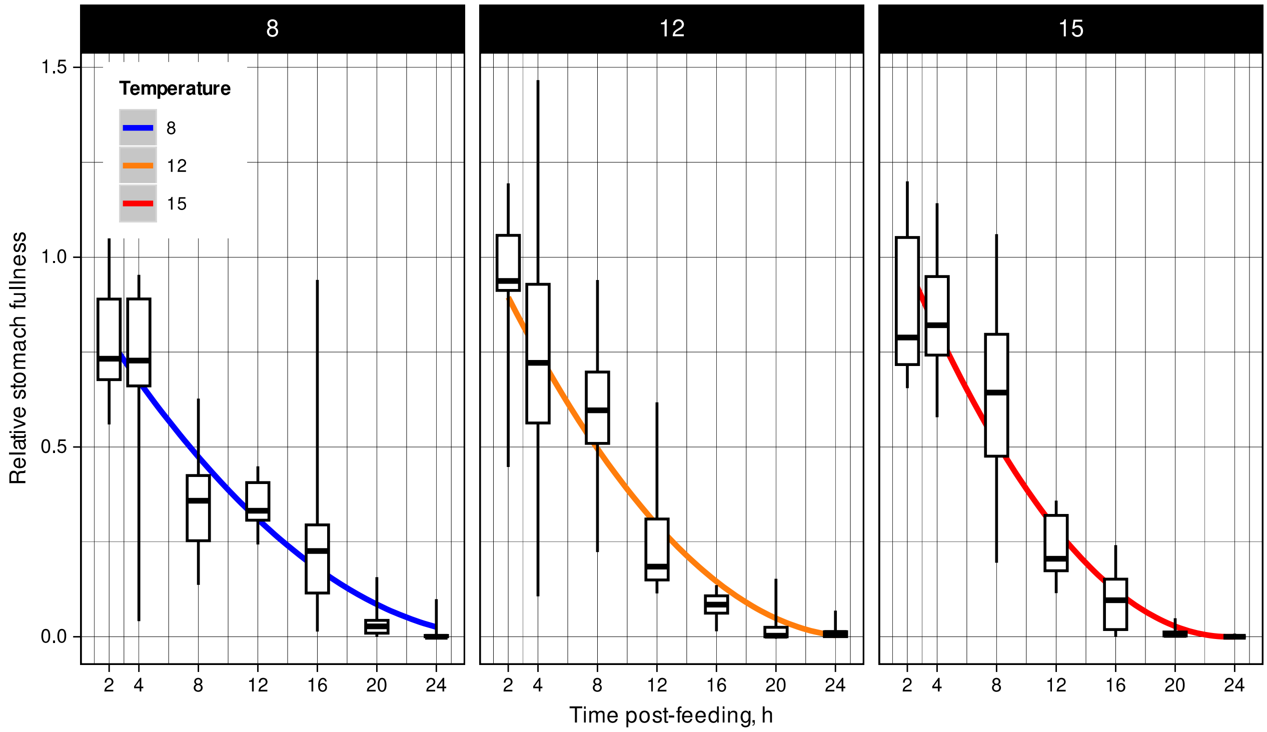

Figure S14. *Dynamics of relative stomach transit with fitted line from the best quadratic model*

Midgut

Table S27. Analysis of the relative midgut fullness (*MIDG*) was based on selecting the best inference model among the following family of models

No	Model	AIC_c	w
7	$MIDG = (H^2) + T + H{:}T$	-816.1	**0.453**
7a	$MIDG = (H^2) + H{:}T$	-814.9	0.247
1	$MIDG = (H^2) \times T$	-813.9	0.157
2	$MIDG = (H^3) \times T$	-812.6	0.079
3	$MIDG = (H^4) \times T$	-811.4	0.044
4	$MIDG = (H^2) + T$	-808.9	0.012
5	$MIDG = (H^3) + T$	-806.9	0.005
6	$MIDG = (H^4) + T$	-805.9	0.003
0	$MIDG = H$	-698.3	0.000

Table S28. Beta coefficients for the best model

Effect	Effect size	Beta±SE	t	p
Intercept		0.182 ± 0.009	18.78	<0.00001
H	-3.38	0.248 ± 0.140	1.77	0.07819
H^2	-8.56	-0.423 ± 0.033	-12.58	<0.00001
T	-0.34	-0.002 ± 0.001	-1.81	0.07156
$T{:}H$	-0.15	-0.0003 ± 0.0001	-3.05	<0.00001

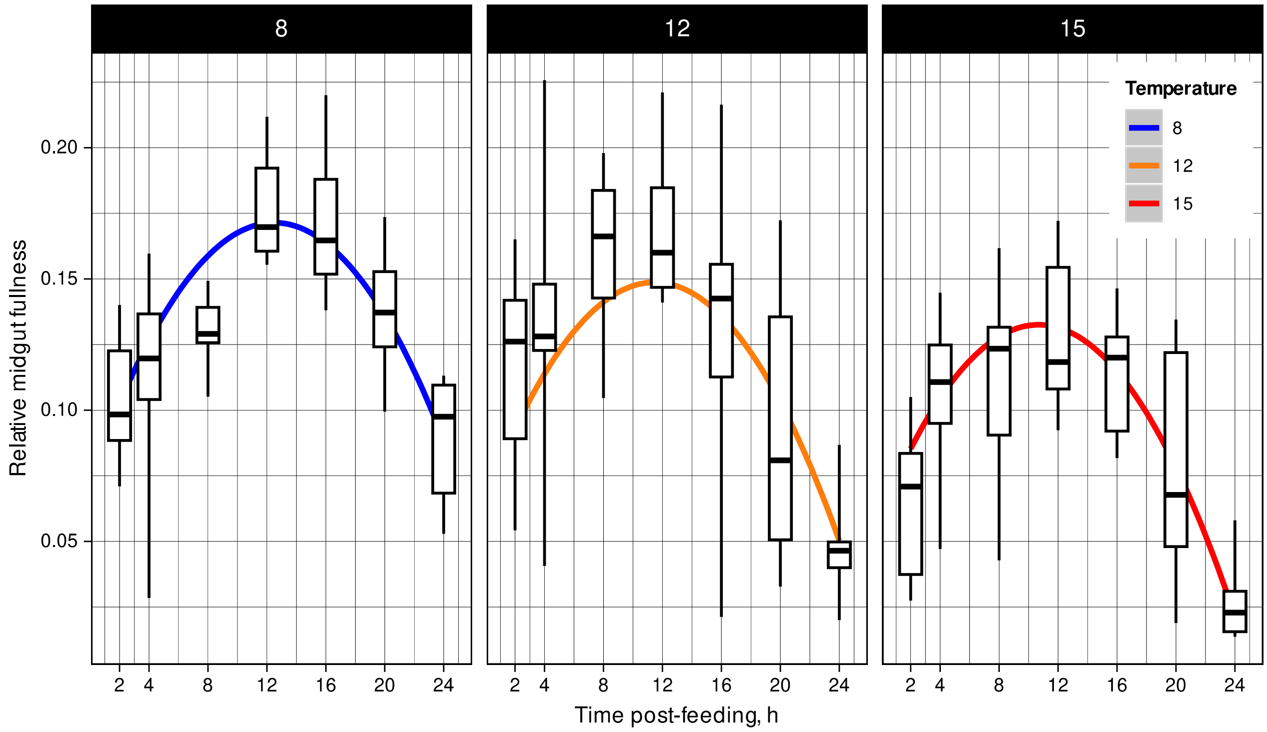

Figure S15. *Dynamics of relative midgut fullness with fitted line from the best model*

Hindgut

Table S29. Analysis of the relative hindgut fullness (*HDG*) was based on selecting the best inference model among the following family of models

No	Model	AIC$_c$	w
1	***HDG = (H²) × T***	-964.0	**0.528**
2	***HDG = (H³) × T***	-963.4	0.395
3	***HDG = (H⁴) × T***	-960.2	0.077
5	***HDG = (H³) + T***	-947.0	0.000
4	***HDG = (H²) + T***	-945.8	0.000
0	***HDG = H***	-934.9	0.000
7	***HDG = (H²) + T + H:T***	-816.1	0.000
7a	***HDG = (H²) + H:T***	-814.9	0.000
6	***HDG = (H⁴) + T***	-944.9	0.000

Table S30. Beta coefficients for the best model

Effect	Effect size	Beta±SE	t	p
Intercept		0.060 ± 0.007	8.757	<0.00001
H	-2.09	-0.139 ± 0.100	-1.387	0.166873
H²	-3.31	0.373 ± 0.100	3.727	0.000251
T	-0.01	-0.0001 ± 0.001	-0.228	0.820207
T:H	0.81	0.007 ± 0.008	0.873	0.383938
T:H²	-4.37	-0.039 ± 0.008	-4.720	<0.00001

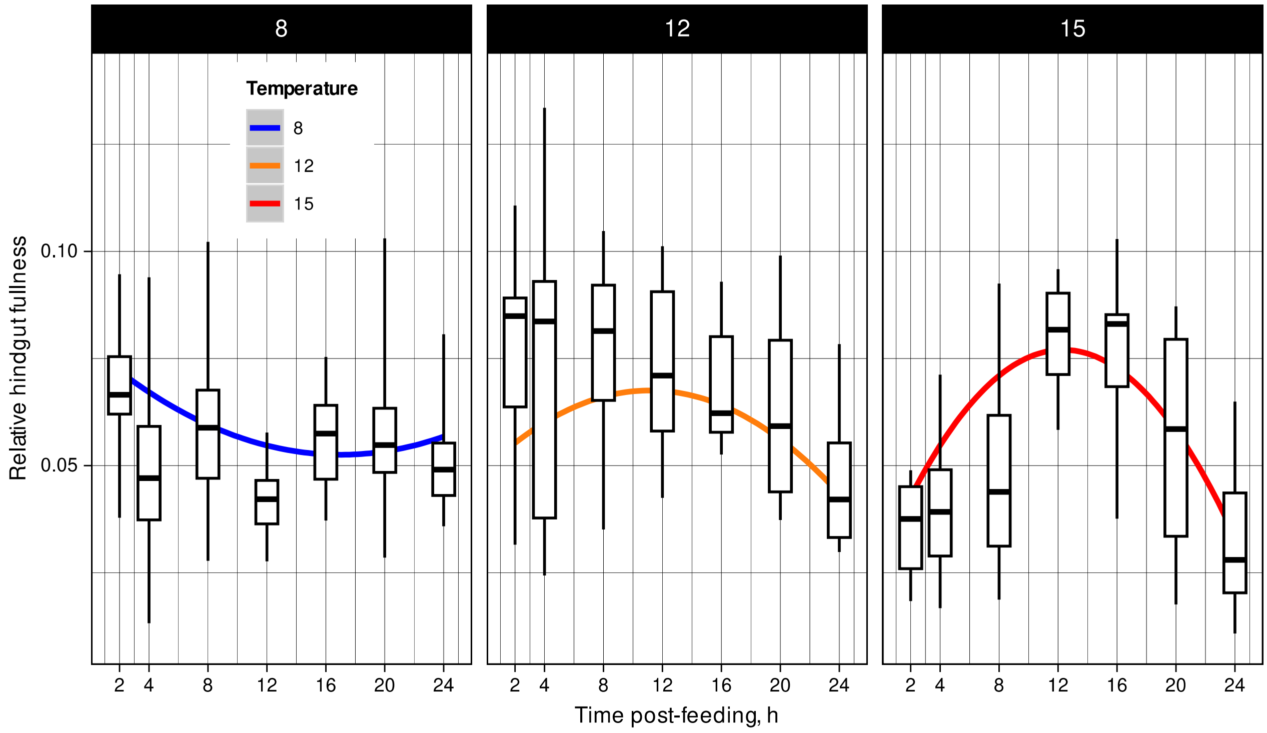

Figure S16. *Dynamics of relative hindgut fullness with fitted line from the best model*

Gall bladder index

Table S31. Analysis of the relative hindgut fullness (*GBI*) was based on selecting the best inference model among the following family of models

No	Model	AIC_c	w
2	***GBI = (H³) × T***	-701.9	**0.647**
1	***GBI = (H²) × T***	-700.0	0.256
3	***GBI = (H⁴) × T***	-698.0	0.092
7a	***GBI = (H²) + H:T***	-690.2	0.002
7	***GBI = (H²) + T + H:T***	-690.1	0.002
7c	***GBI = (H³) + H:T***	-688.3	0.001
7b	***GBI = (H³) + T + H:T***	-688.2	0.001
4	***GBI = (H²) + T***	-671.5	0.000
5	***GBI = (H³) + T***	-669.5	0.000
6	***GBI = (H⁴) + T***	-667.6	0.000
0	***GBI = H × T***	-629.5	0.000
0b	***GBI = H + T***	-616.0	0.000
0c	***GBI = H***	-607.8	0.000
0a	***GBI = T***	-559.5	0.000

Table S32. Beta coefficients for the best model

Effect	Effect size	Beta±SE	t	p
Intercept		0.020 ± 0.013	1.57	0.117301
H	7.00	-0.413 ± 0.186	-2.22	0.027368
H²	6.05	-0.244 ± 0.186	-1.31	0.190581
H³	-0.31	-0.456 ± 0.186	-2.46	0.014893
T	0.19	0.004 ± 0.001	4.01	<0.00001
T* : *H	3.31	0.074 ± 0.015	4.78	<0.00001
T* : *H²	2.42	0.054 ± 0.015	3.51	0.000561
T* : *H³	1.67	0.037 ± 0.015	2.42	0.016388

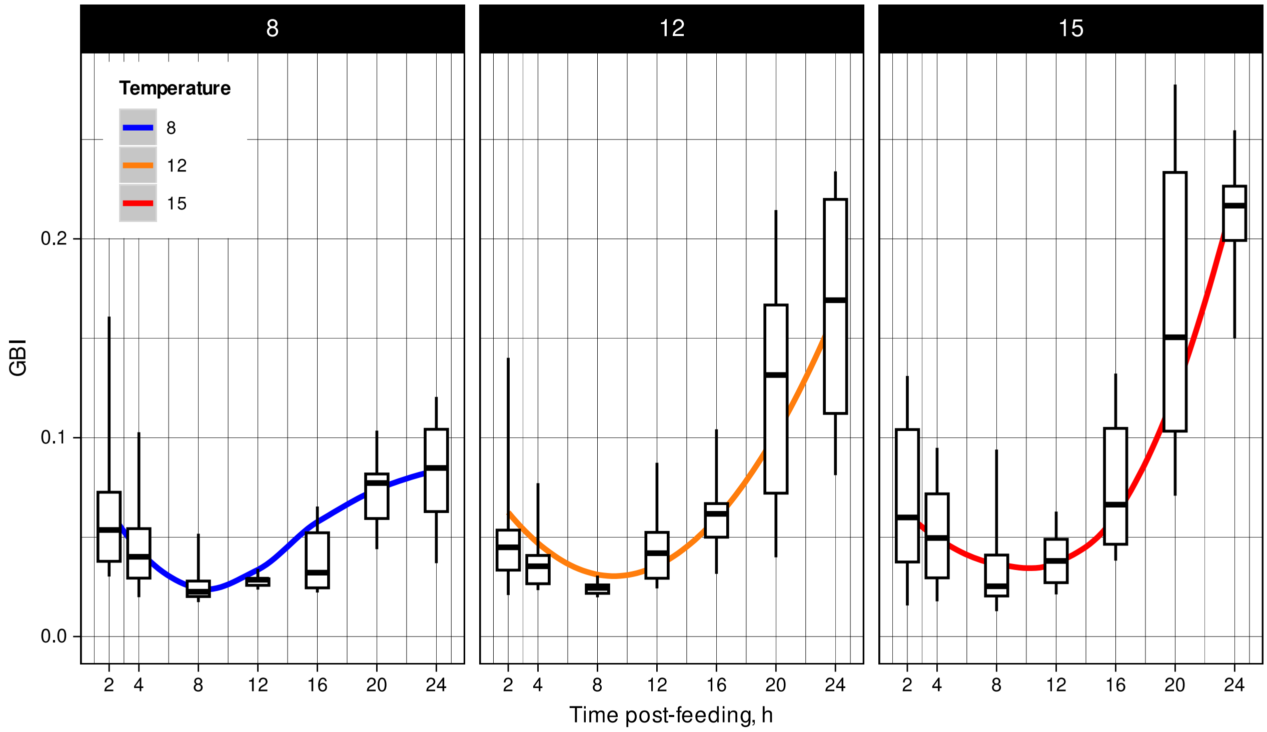

Figure S17. *Dynamics of gall bladder index (GBI) with fitted line from the best model*

Metabolites and ghrelin plasma levels

Glucose

Table S33. Analysis of the plasma glucose was based on selecting the best inference model among the following family of models

No	Model	AIC_c	w
3	***Glucose* = H^4 × *T***	367.1	**0.439**
2	***Glucose* = H^3 × *T***	367.9	0.305
4	***Glucose* = H^5 × *T***	368.3	0.249
6	***Glucose* = H^4 + *T***	376.2	0.005
5	***Glucose* = H^3 + *T***	377.8	0.002
1	***Glucose* = H^2 × *T***	396.6	0.000
0	***Glucose* = *H* × *T***	414.4	0.000
0a	***Glucose* = *H***	427.0	0.000
0b	***Glucose* = *T***	441.5	0.000

Table S34. Beta coefficients for the best model

Effect	Effect size	Beta±SE	t	p
Intercept		4.735 ± 0.169	28.00	<0.00001
H	4.86	-1.745 ± 2.457	0.71	0.478387
H^2	3.40	9.357 ± 2.461	3.80	0.000192
H^3	-4.46	1.047 ± 2.447	0.43	0.669303
H^4	1.57	-1.295 ± 2.449	-0.53	0.597528
T	0.23	0.059 ± 0.014	4.16	<0.00001
T* : *H	1.78	0.457 ± 0.206	2.22	0.027563
T : H^2	-2.27	-0.586 ± 0.206	-2.84	0.005049
T : H^3	-1.47	-0.376 ± 0.204	-1.84	0.067472
T : H^4	0.82	0.213 ± 0.205	1.04	0.299454

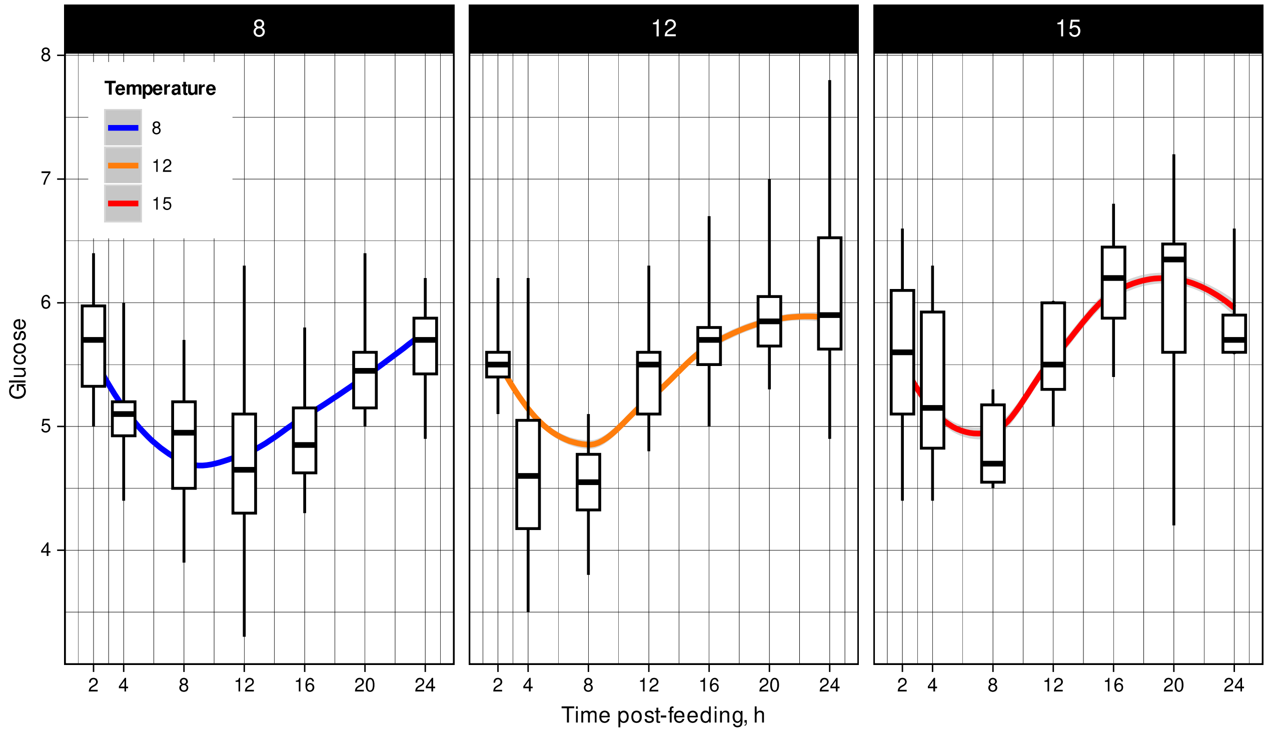

Figure S18. *Dynamics of plasma glucose with fitted line from the best model*

Cholesterol

Table S35. Analysis of the plasma cholesterol was based on selecting the best inference model among the following family of models

**No**	**Model**	**AIC_c**	**w**
3	***Cholesterol* = *H^2* + *T***	684.5	**0.697**
1	***Cholesterol* = *H^2* × *T***	686.7	0.230
2	***Cholesterol* = *H^3* × *T***	689.0	0.073
00	***Cholesterol* = *T***	706.3	0.000
0b	***Cholesterol* = *H* + *T***	706.4	0.000
0	***Cholesterol* = *H* × *T***	706.8	0.000
0a	***Cholesterol* = *H***	717.5	0.000

Table S36. Beta coefficients for the best model

**Effect**	**Effect size**	**Beta±SE**	**t**	**p**
Intercept		7.173 ± 0.378	19.02	<0.00001
H	1.36	1.911 ± 1.324	1.44	0.150334
H^2	4.58	6.639 ± 1.327	5.00	<0.00001
T	0.25	0.124 ± 0.032	3.92	0.000123

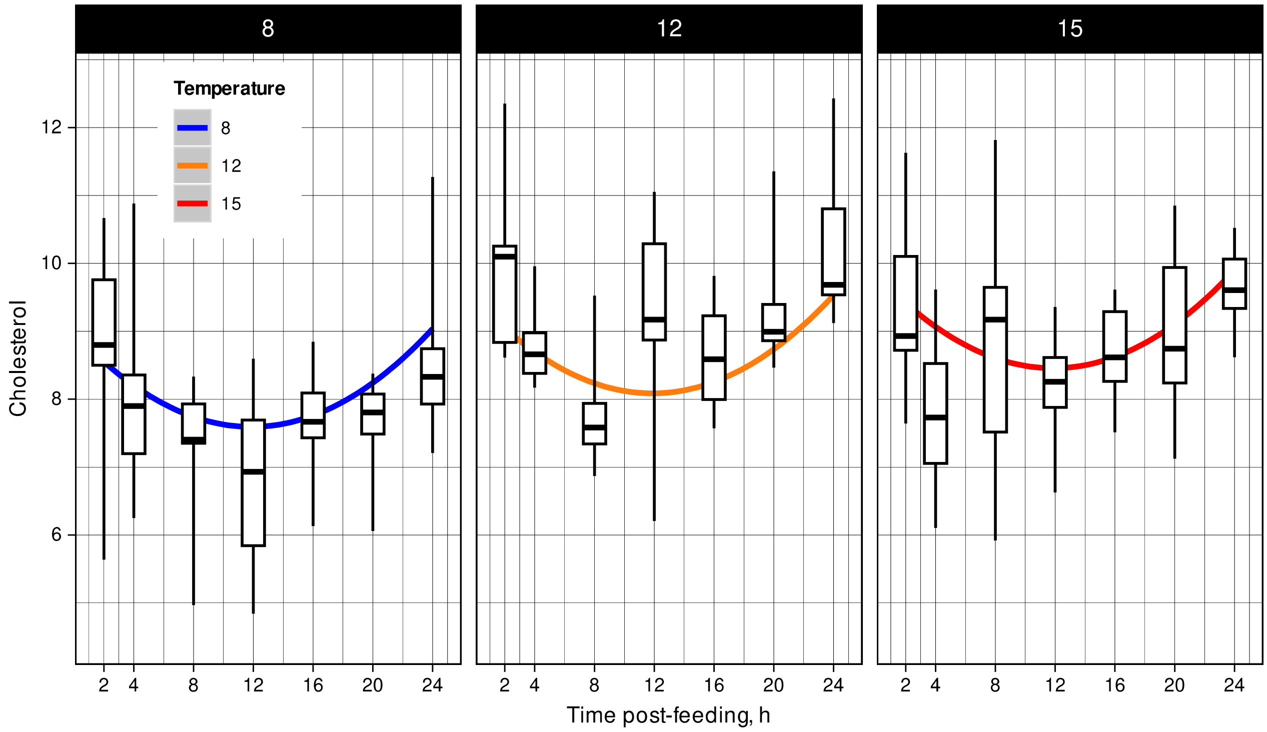

Figure S19. *Dynamics of plasma cholesterol with fitted line from the best model*

Lactate

Table S37. Analysis of the plasma lactate was based on selecting the best inference model among the following family of models

No	Model	AIC_c	w
2a	$\sqrt{Lactate} = (\boldsymbol{H}^3) + \boldsymbol{T} + \boldsymbol{H}^2 : \boldsymbol{T}$	-52.8	**0.360**
2	$\sqrt{Lactate} = (\boldsymbol{H}^3) \times \boldsymbol{T}$	-52.6	0.316
5	$\sqrt{Lactate} = (\boldsymbol{H}^3) + \boldsymbol{T}$	-51.0	0.143
00	$\sqrt{Lactate} = \boldsymbol{T}$	-49.4	0.065
3	$\sqrt{Lactate} = (\boldsymbol{H}^4) \times \boldsymbol{T}$	-49.2	0.059
1	$\sqrt{Lactate} = (\boldsymbol{H}^2) \times \boldsymbol{T}$	-47.9	0.031
0	$\sqrt{Lactate} = \boldsymbol{H} \times \boldsymbol{T}$	-46.2	0.013
4	$\sqrt{Lactate} = (\boldsymbol{H}^2) + \boldsymbol{T}$	-46.2	0.013
0a	$\sqrt{Lactate} = \boldsymbol{H}$	-17.4	0.000

Table S38. Beta coefficients for the best model

Effect	Effect size	Beta±SE	t	p
Intercept		1.527 ± 0.060	25.33	<0.00001
$\boldsymbol{H}$	-0.80	0.162 ± 0.876	0.19	0.85369
$\boldsymbol{H}^2$	-0.47	1.959 ± 0.878	2.23	0.02674
$\boldsymbol{H}^3$	2.39	0.558 ± 0.211	2.64	0.00893
$\boldsymbol{T}$	0.39	0.030 ± 0.004	6.03	<0.00001
$\boldsymbol{T} : \boldsymbol{H}$	-0.39	-0.030 ± 0.073	-0.41	0.68628
$\boldsymbol{T} : \boldsymbol{H}^2$	-2.21	-0.179 ± 0.074	-2.43	0.01620

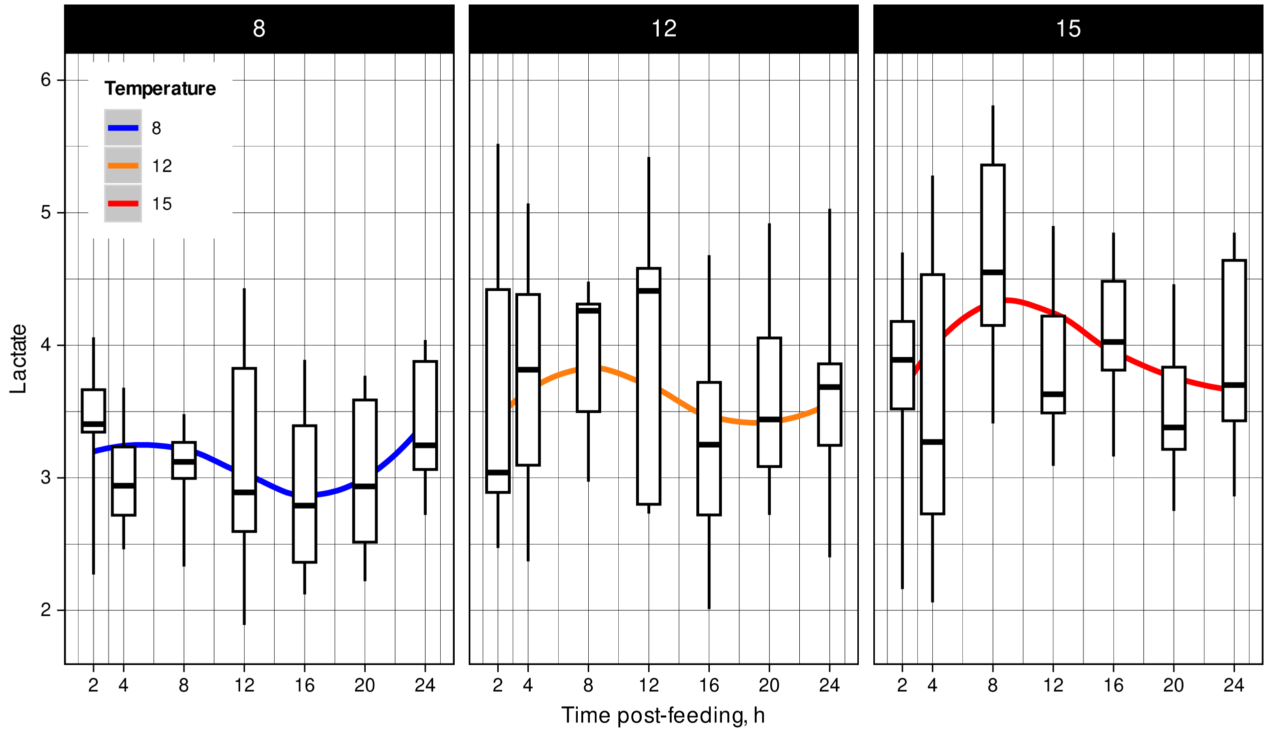

Figure S20. *Dynamics of plasma lactate with fitted line from the best model*

Triglyceride

Table S39: Analysis of the plasma triglyceride was based on selecting the best inference model among the following family of models

No	Model	AIC_c	w
7	***Trig = H + T:(H²)***	585.6	**0.467**
6	***Trig = (H²) + T:(H²)***	586.9	0.245
1	***Trig = (H²) × T***	587.2	0.211
2	***Trig = (H³) × T***	589.7	0.062
4	***Trig = (H²) + T + T:H***	592.7	0.014
3	***Trig = (H²) + T***	600.4	0.000
5	***Trig = (H³) + T***	600.9	0.000
0	***Trig = H* × T**	620.2	0.000
0b	***Trig* = T**	627.0	0.000
0a	***Trig = H***	627.1	0.000
00	***Trig = H* + T**	628.0	0.000

Table S40. Beta coefficients for the best model

Effect	Effect size	Beta±SE	t	p
Intercept		1.698 ± 0.414	4.10	<0.00001
H	0.07	0.145 ± 0.040	3.64	0.000348
T : H	-3.11	-1.260 ± 0.365	-3.45	0.000678
T : H²	-2.08	-0.550 ± 0.089	-6.21	<0.00001

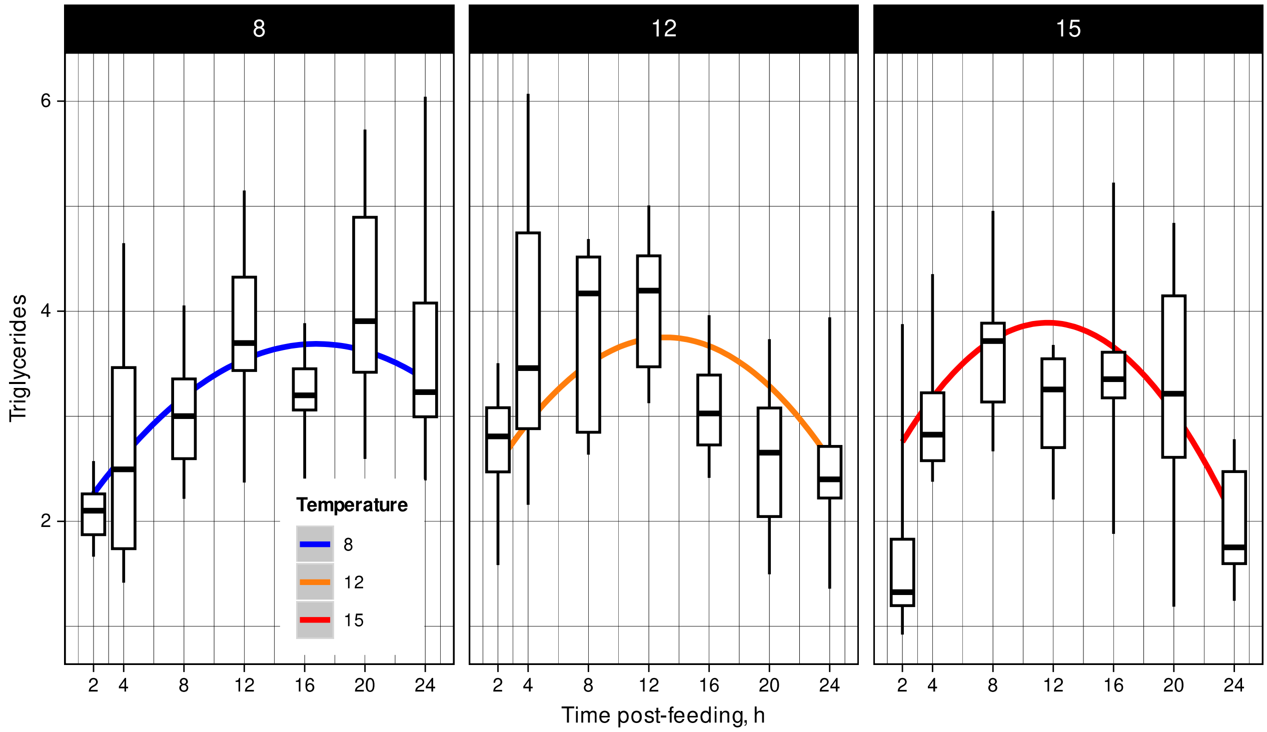

Figure S21. *Dynamics of plasma triglyceride with fitted line from the best model*

Ghrelin

Table S41: Analysis of the plasma ghrelin was based on selecting the best inference model among the following family of models

No	Model	AIC_c	w
6	***GhRL = H² + T:H²***	1544.7	**0.308**
1	***GhRL = H² × T***	1545.2	0.242
7	***GhRL = H + T:H²***	1545.5	0.212
2	***GhRL = H³ × T***	1545.7	0.189
4	***GhRL = H² + T + T:H***	1548.7	0.043
0	***GhRL = H × T***	1552.9	0.005
3	***GhRL = H² + T***	1556.4	0.001
5	***GhRL = H² +* T:*H***	1557.4	0.001
0a	***GhRL = H***	1559.7	0.000
0b	***GhRL = T***	1563.3	0.000

Table S42. Beta coefficients for the best model

Effect	Effect size	Beta±SE	t	p
Intercept		52.513 ± 0.704	74.60	<0.00001
H	-2.34	106.867 ± 42.522	2.51	0.01275
H²	-2.42	71.402 ± 42.503	1.68	0.09452
T : H	-2.97	-11.408 ± 3.552	-3.21	0.00153
T : H²	-2.23	-8.376 ± 3.549	-2.36	0.01922

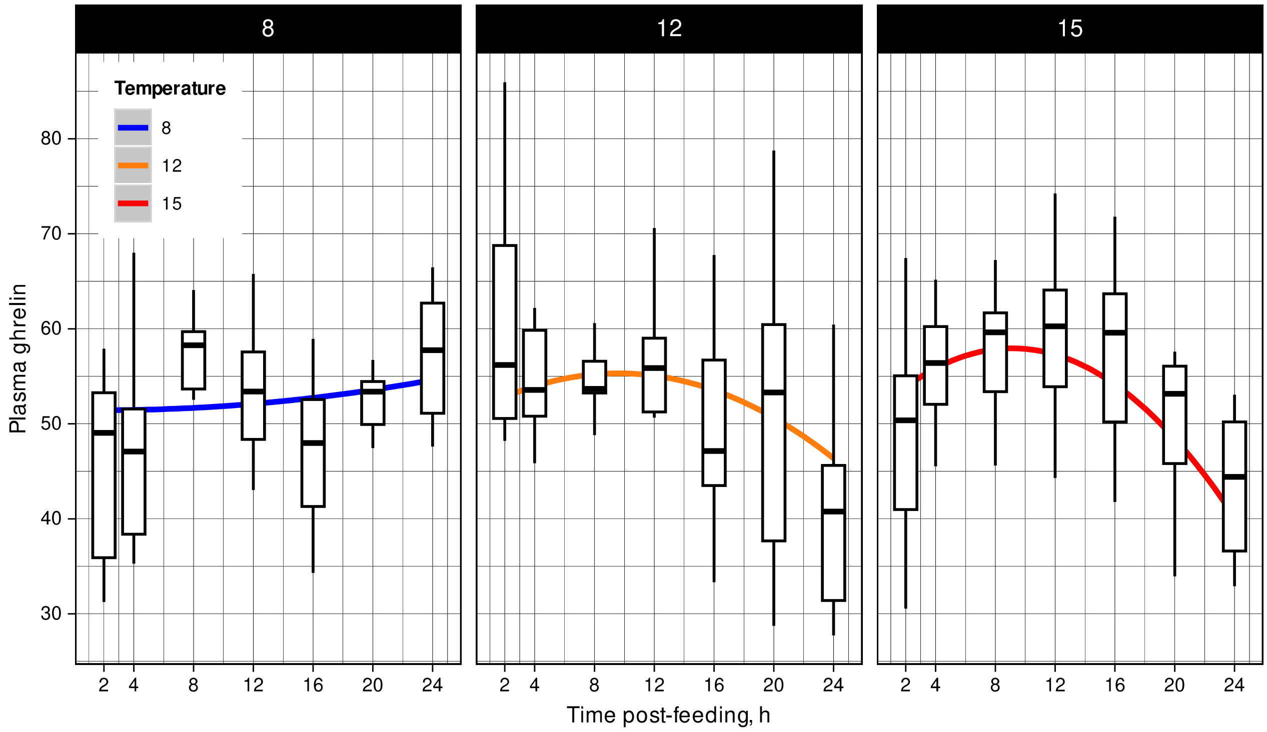

Figure S22. *Dynamics of plasma ghrelin with fitted line from the best model*

Stomach mRNA expression levels

ghrl1

Table S43: Analysis of the ghrl1 expression was based on selecting the best inference model among the following family of models

No	Model	AIC$_c$	w
5	***ghrl1*** $= H^2 + T{:}H$	2990.0	**0.514**
4	***ghrl1*** $= H^2 + T + T{:}H$	2991.9	0.194
1	***ghrl1*** $= H^2 \times T$	2993.0	0.114
3	***ghrl1*** $= H^2 + T$	2993.7	0.078
2	***ghrl1*** $= H^3 \times T$	2993.8	0.076
0	***ghrl1*** $= H \times T$	2998.0	0.009
0b	***ghrl1*** $= T$	2998.0	0.009
0c	***ghrl1*** $= H + T$	2999.7	0.004
0a	***ghrl1*** $= H$	3001.4	0.002

Table S44: Beta coefficients for the best model

Effect	Effect size	Beta±SE	t	p
Intercept		864.953 ± 69.551	12.44	<0.00001
H	-0.63	1786.566 ± 769.971	2.32	0.02130
H^2	2.77	842.150 ± 294.454	2.86	0.00467
$T : H$	-0.13	-1.540 ± 0.554	-2.780	0.00597

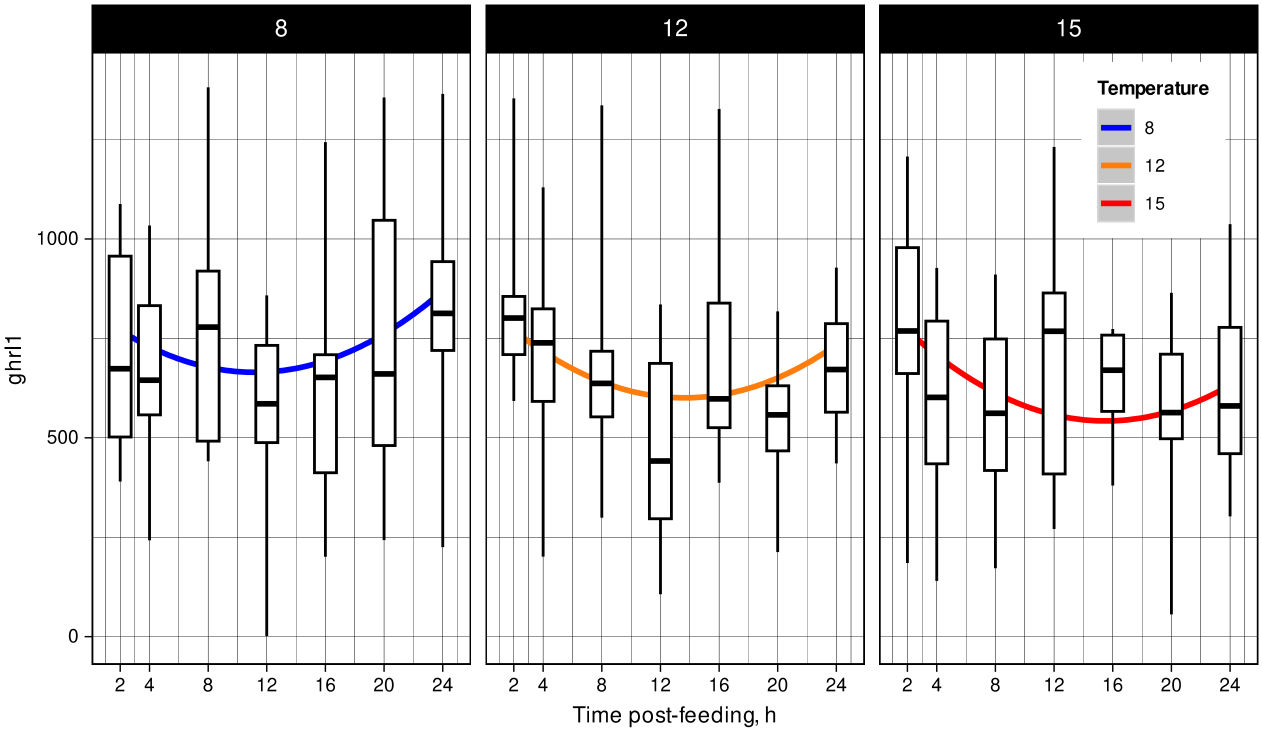

Figure S23. *Dynamics of ghrl1 expression in stomach with fitted line from the best model*

ghrl2

Table S45: Analysis of the *ghrl2* expression was based on selecting the best inference model among the following family of models

No	Model	AIC_c	w
5	*ghrl2* = $H^2 + T{:}H$	3919.8	**0.217**
6	*ghrl2* = $H^2 + T{:}H^2$	3920.0	0.204
7	*ghrl2* = H^2	3920.0	0.196
4	*ghrl2* = $H^2 + T + T{:}H$	3920.7	0.138
1	*ghrl2* = $H^2 \times T$	3921.8	0.082
3	*ghrl2* = $H^2 + T$	3921.8	0.080
2	*ghrl2* = $H^3 \times T$	3922.7	0.052
0b	*ghrl2* = T	3925.9	0.010
0a	*ghrl2* = H	3926.0	0.010
0	*ghrl2* = $H \times T$	3926.8	0.007
0c	*ghrl2* = $H + T$	3927.8	0.004

Table S46: Beta coefficients for the best model

Effect	Effect size	Beta±SE	t	p
Intercept		7613.252 ± 636.536		<0.00001
H	-0.49	8460.568 ± 7046.821		0.23128
H^2	2.80	7678.986 ± 2694.864		0.00482
$T : H$	-0.12	-7.641 ± 5.073		0.13355

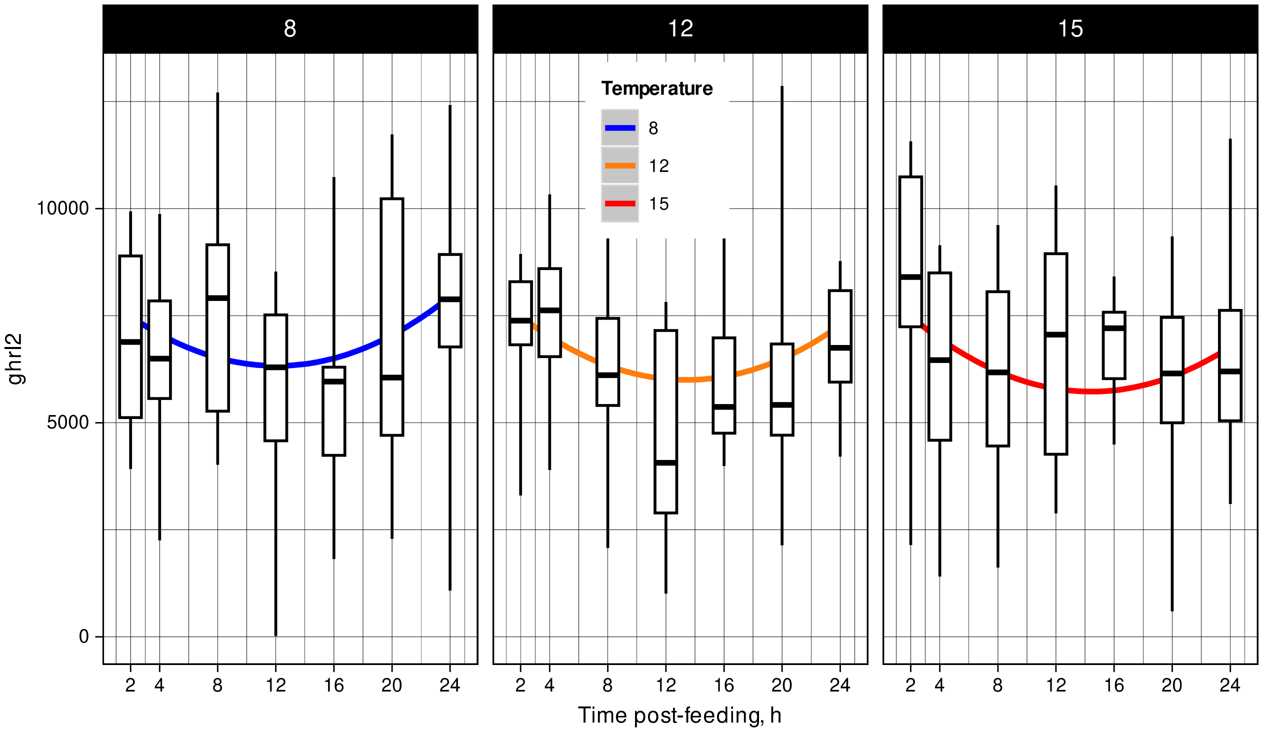

Figure S24. *Dynamics of ghrl2 expression in stomach with fitted line from the best model*

mboat

Table S47: Analysis of the *mboat* expression was based on selecting the best inference model among the following family of models

No	Model	AIC$_c$	w
4	$mboat = H^2$	898.0	**0.242**
3	$mboat = H^2 + T$	898.5	0.188
6	$mboat = H^2 + T{:}H^2$	898.5	0.181
1	$mboat = H^2 \times T$	899.0	0.142
5	$mboat = H^2 + T{:}H$	899.9	0.091
2	$mboat = H^3 \times T$	901.4	0.044
0b	$mboat = H$	901.4	0.043
0a	$mboat = H + T$	902.0	0.032
0	$mboat = H \times T$	903.0	0.020
0c	$mboat = T$	903.2	0.018

Table S48: Beta coefficients for the best model

Effect	Effect size	Beta±SE	t	p
Intercept		5.560 ± 0.141	39.24	<0.00001
H	-0.77	3.706 ± 2.048	-1.81	0.0719
H^2	1.00	4.836 ± 2.056	2.35	0.0196

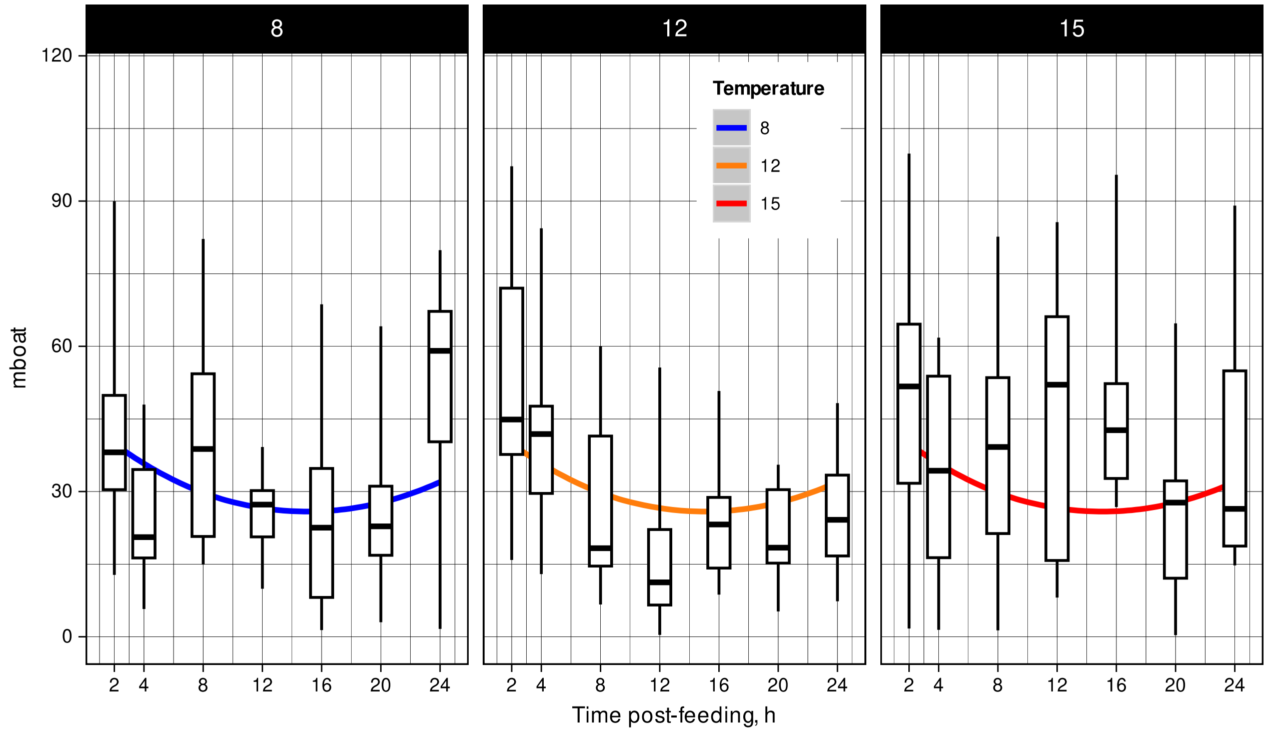

Figure S25. *Dynamics of mboat expression in stomach with fitted line from the best model*

Hypothalamic mRNA expression levels

npya1

Table S49: Analysis of the *npya1* expression was based on selecting the best inference model among the following family of models

No	Model	AIC$_c$	w
1	***npya1 = H² × T***	2282.0	**0.513**
2	***npya1 = H³ × T***	2282.8	0.356
0	***npya1 = H × T***	2286.2	0.065
0a	***npya1 = H + T***	2287.0	0.042
3	***npya1 = H² + T***	2288.9	0.016
4	***npya1 = H³ + T***	2290.5	0.008
0b	***npya1 = H***	2310.5	0.000
0c	***npya1 = T***	2328.9	0.000

Table S50: Beta coefficients for the best model

Effect	Effect size	Beta±SE	t	p
Intercept		309.224 ± 19.337	15.991	<0.00001
H	6.03	6.713 ± 280.184	0.024	0.98091
H²	0.41	-740.317 ± 280.924	-2.635	0.00908
T	-0.31	-8.452 ± 1.610	-5.251	<0.00001
T:H	1.47	40.462 ± 23.395	1.730	0.08528
T:H²	2.38	66.232 ± 23.341	2.838	0.00502

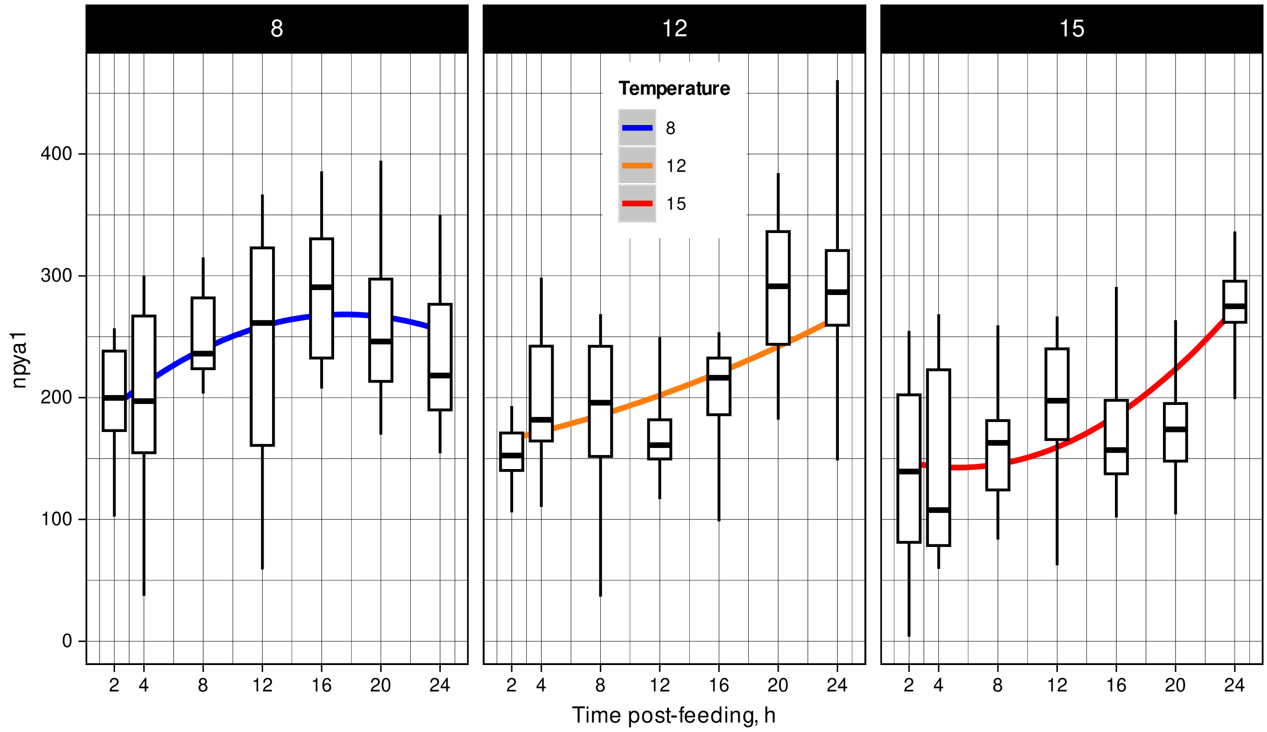

Figure S26. *Dynamics of npya1 expression in the brain with fitted line from the best model*

npya2

Table S51: Analysis of the *npya2* expression was based on selecting the best inference model among the following family of models

**No**	**Model**	**AIC$_c$**	**w**
0c	***npya2 = H + T***	3308.0	**0.414**
3	***npya2 = H² + T***	3308.7	0.292
0	***npya2 = H × T***	3310.0	0.147
4	***npya2 = H³ + T***	3310.8	0.102
1	***npya2 = H² × T***	3312.7	0.039
2	***npya2 = H³ × T***	3317.0	0.005
0a	***npya2 = T***	3331.4	0.000
0b	***npya2 = H***	3366.0	0.000

Table S52: Beta coefficients for the best model

**Effect**	**Effect size**	**Beta±SE**	**t**	**p**
Intercept		5097.475 ± 277.234	18.39	<0.00001
H		42.832 ± 8.273	5.18	<0.00001
T		-182.768 ± 22.005	-8.31	<0.00001

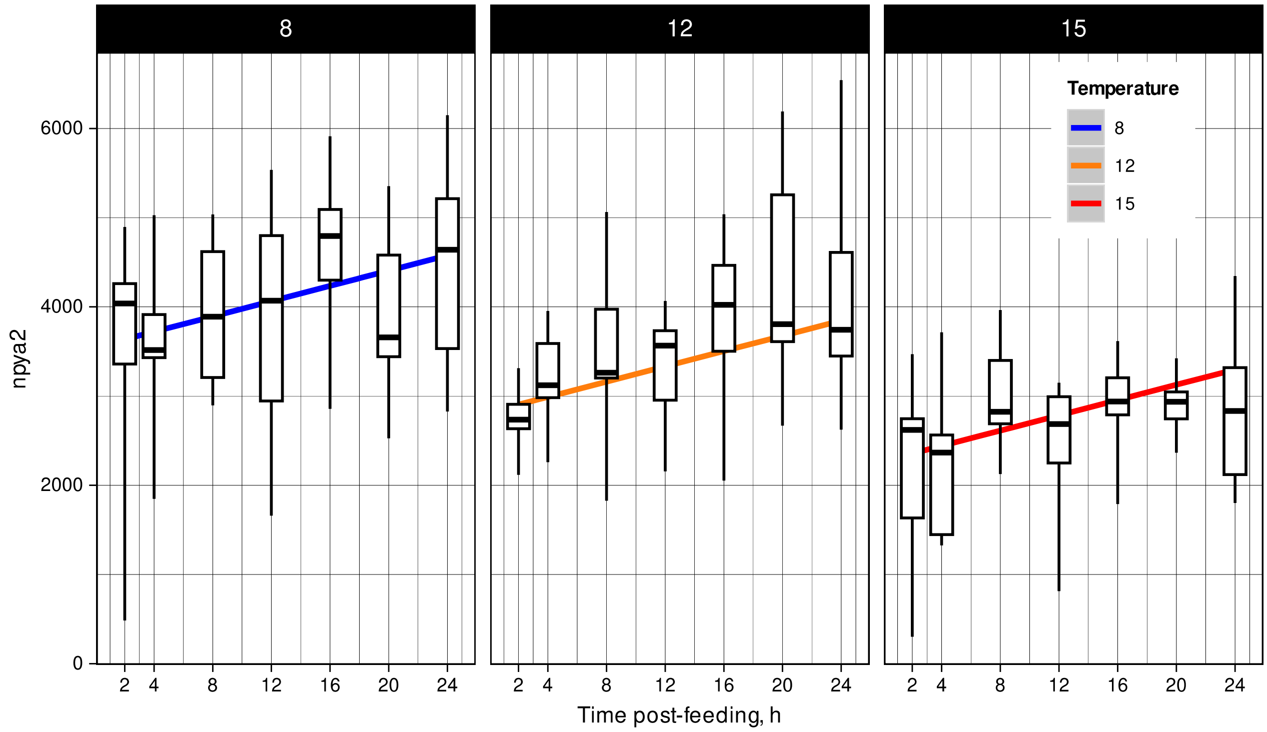

Figure S27. *Dynamics of npya2 expression in the brain with fitted line from the best model*

pomca1

Table S53: Analysis of the *pomca1* expression was based on selecting the best inference model among the following family of models

No	Model	AIC_c	w
3	*ln(pomca1)* = ***H² + T***	471.2	**0.563**
4	*ln(pomca1)* = ***H³ + T***	472.9	0.236
1	*ln(pomca1)* = ***H² × T***	474.3	0.118
0a	*ln(pomca1)* = ***H + T***	476.6	0.038
0	*ln(pomca1)* = ***H × T***	477.5	0.024
2	*ln(pomca1)* = ***H³ × T***	478.3	0.016
0b	*ln(pomca1)* = ***H***	481.3	0.004
0c	*ln(pomca1)* = ***T***	482.5	0.002

Table S54: Beta coefficients for the best model

Effect	Effect size	Beta±SE	t	p
Intercept		6.752 ± 0.225	30.03	<0.00001
H	2.75	2.229 ± 0.778	2.87	0.00457
H²	-2.61	-2.129 ± 0.778	-2.74	0.00674
T	-0.18	-0.051 ± 0.019	-2.71	0.00722

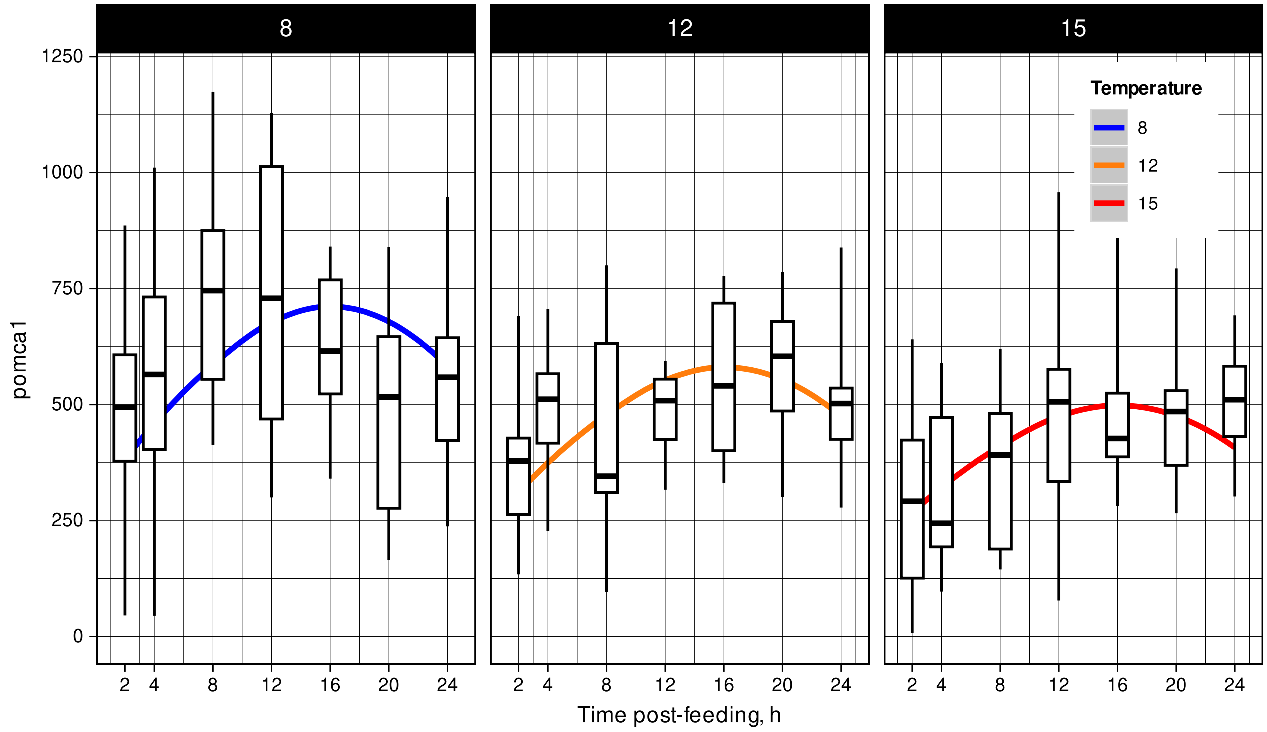

Figure S28. *Dynamics of pomca1 expression in the brain with fitted line from the best model*

pomca2

Table S55: Analysis of the *pomca2* expression was based on selecting the best inference model among the following family of models

No	Model	AIC_c	w
3	*ln(pomca2) = H² + T*	441.1	**0.490**
0a	*ln(pomca2) = H + T*	443.1	0.181
4	*ln(pomca2) = H³ + T*	443.1	0.180
0	*ln(pomca2) = H × T*	445.2	0.063
1	*ln(pomca2) = H² × T*	445.2	0.063
0c	*ln(pomca2) = T*	448.2	0.014
2	*ln(pomca2) = H³ × T*	449.0	0.010
0b	*ln(pomca2) = H*	466.9	0.000

Table S56: Beta coefficients for the best model

Effect	Effect size	Beta±SE	t	p
Intercept		6.597 ± 0.213	31.03	<0.00001
H	2.49	1.978 ± 0.743	2.66	0.00842
H²	-1.85	-1.497 ± 0.743	-2.01	0.04547
T	-0.35	-0.094 ± 0.018	-5.30	<0.00001

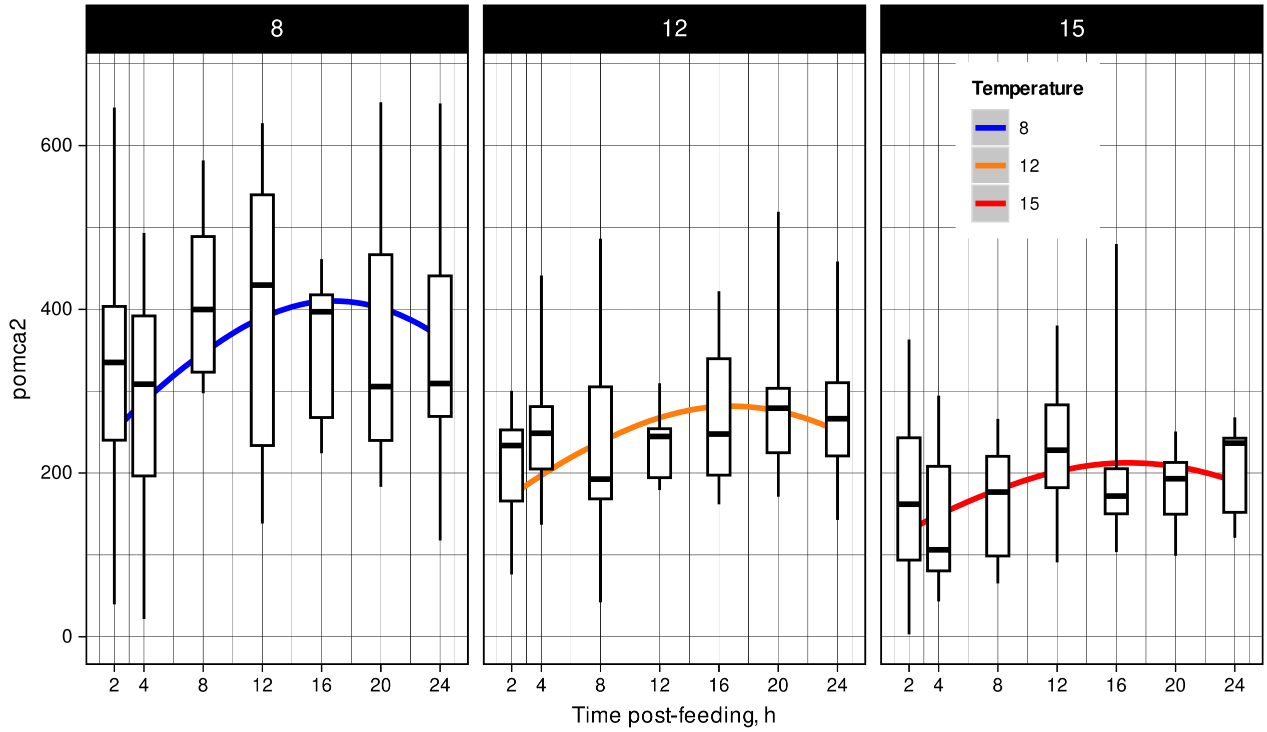

Figure S29. *Dynamics of pomca2 expression in the brain with fitted line from the best model*

agrp1

Table S57: Analysis of the *agrp1* expression was based on selecting the best inference model among the following family of models

No	Model	AIC_c	w
0a	$\sqrt{agrp1} = \boldsymbol{H}$	1432.4	**0.321**
0	$\sqrt{agrp1} = \boldsymbol{H} \times \boldsymbol{T}$	1433.3	0.204
0c	$\sqrt{agrp1} = \boldsymbol{H} + \boldsymbol{T}$	1434.1	0.140
3	$\sqrt{agrp1} = (\boldsymbol{D}^2) + T$	1434.3	0.124
00	$\sqrt{agrp1} = c$ (constant)	1435.5	0.068
1	$\sqrt{agrp1} = (\boldsymbol{D}^2) \times T$	1435.7	0.061
4	$\sqrt{agrp1} = (\boldsymbol{D}^3) + T$	1436.3	0.046
0b	$\sqrt{agrp1} = \boldsymbol{T}$	1437.2	0.029
2	$\sqrt{agrp1} = (\boldsymbol{D}^3) \times T$	1439.9	0.008

Table S58: Beta coefficients for the best model

Effect	Effect size	Beta±SE	t	p
Intercept		24.822 ± 0.998	24.89	<0.00001
H	0.16	0.178 ± 0.079	2.28	0.0238

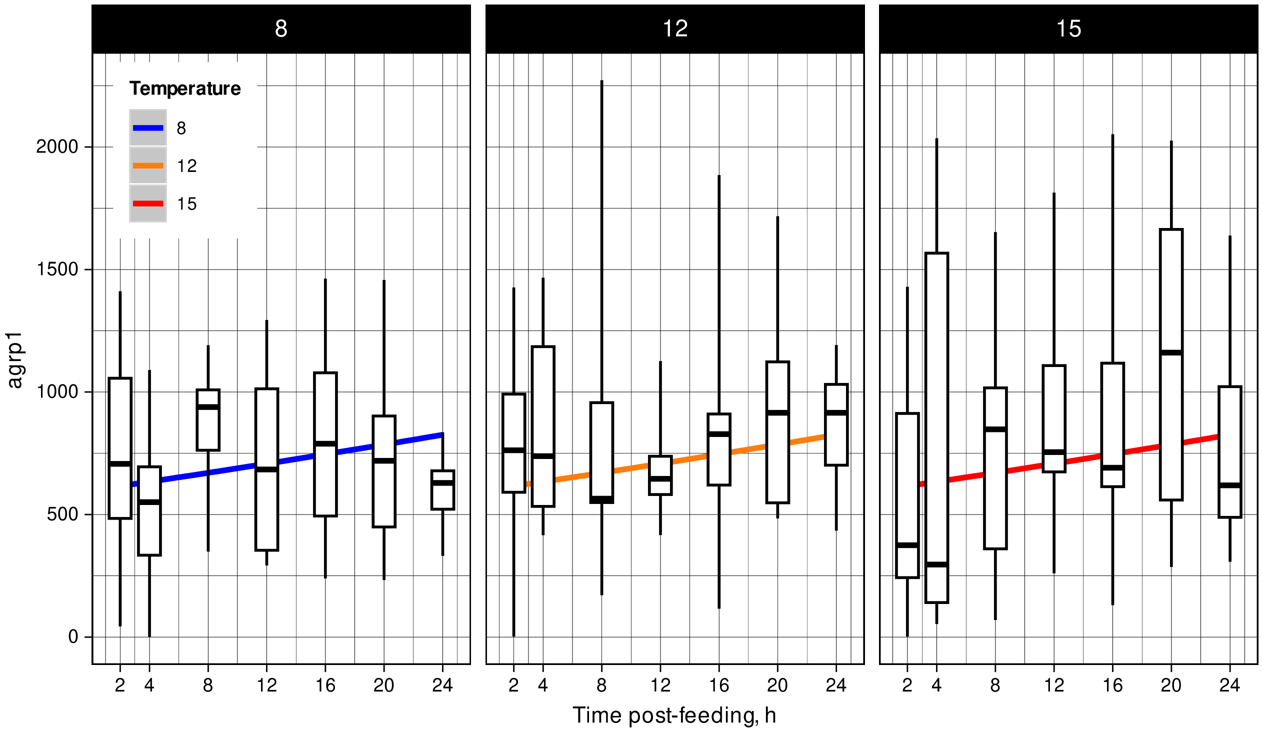

Figure S30. *Dynamics of agrp1 expression in the brain with fitted line from the best model*

cart2b

Table S59: Analysis of the *cart2b* expression was based on selecting the best inference model among the following family of models

No	Model	AIC_c	w
0c	***cart2b* = *H***	4090.5	**0.279**
0	***cart2b* = *H* × *T***	4091.2	0.200
0a	***cart2b* = *H* + *T***	4091.3	0.187
3	***cart2b* = *H*² + *T***	4091.9	0.142
2	***cart2b* = *H*³ × *T***	4093.4	0.067
4	***cart2b* = *H*³ + *T***	4093.5	0.064
1	***cart2b* = *H*² × *T***	4093.7	0.058
0b	***cart2b* = *T***	4100.6	0.002

Table S60: Beta coefficients for the best model

Effect	Effect size	Beta±SE	t	p
Intercept		19807.9 ± 742.0	26.695	<0.00001
H	0.23	197.3 ± 58.1	3.395	0.000827

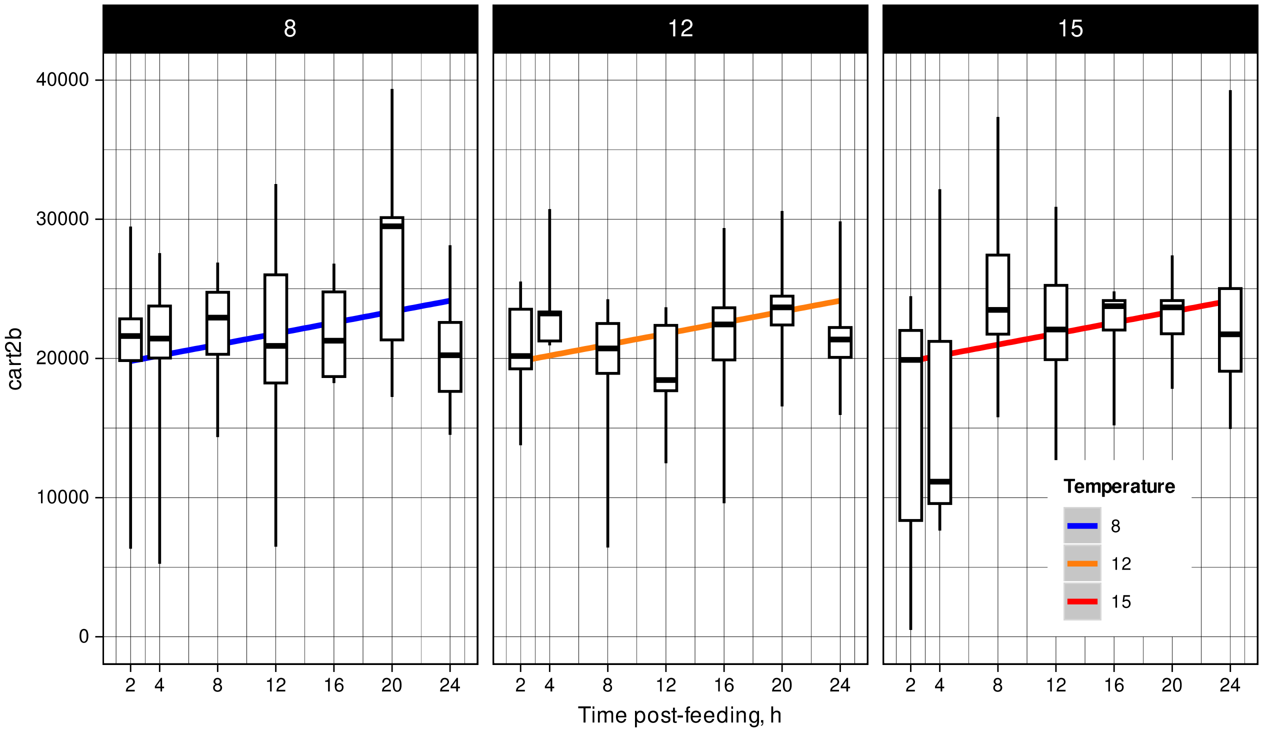

Figure S31. *Dynamics of cart2b expression in the brain with fitted line from the best model*

MATURATION

General information

Table S61: GSI of males and females over the eight-week study

Sex	Category	week 0	week 2	week 4	week 6	week 8
Males	8˚C					
	Immature (GSI<0.06%)	100% (7)	71% (5)	100% (8)	100% (6)	91% (30)
	Maturing (GSI>0.06%)	0% (0)	29% (2)	0% (0)	0% (0)	9% (3)
	Total:	100% (7)	100% (7)	100% (8)	100% (6)	100% (33)
	12˚C					
	Immature (GSI<0.06%)	100% (7)	60% (3)	88% (7)	86% (6)	97% (38)
	Maturing (GSI>0.06%)	0% (0)	40% (2)	13% (1)	14% (1)	3% (1)
	Total:	100% (7)	100% (5)	100% (8)	100% (7)	100% (39)
	15˚C					
	Immature (GSI<0.06%)	100% (5)	57% (4)	100% (10)	86% (6)	77% (27)
	Maturing (GSI>0.06%)	0% (0)	43% (3)	0% (0)	14% (1)	23% (8)
	Total:	100% (5)	100% (7)	100% (10)	100% (7)	100% (35)

Sex	Category	week 0	week 2	week 4	week 6	week 8
Females	8˚C					
	Immature (GSI<0.20%)	100% (8)	100% (8)	100% (7)	100% (9)	100% (37)
	Maturing (GSI>0.20%)	0% (0)	0% (0)	0% (0)	0% (0)	0% (0)
	Total:	100% (8)	100% (8)	100% (7)	100% (9)	100% (37)
	12˚C					
	Immature (GSI<0.20%)	100% (8)	100% (10)	100% (7)	100% (8)	100% (31)
	Maturing (GSI>0.20%)	0% (0)	0% (0)	0% (0)	0% (0)	0% (0)
	Total:	100% (8)	100% (10)	100% (7)	100% (8)	100% (31)
	15˚C					
	Immature (GSI<0.20%)	100% (10)	100% (8)	100% (5)	100% (8)	100% (35)
	Maturing (GSI>0.20%)	0% (0)	0% (0)	0% (0)	0% (0)	0% (0)
	Total:	100% (10)	100% (8)	100% (5)	100% (8)	100% (35)

Note: The percentage indicates the presence of immature or maturing fish relative to the sampled fish at that specific time point. The number of fish scored for each sampling point is indicated in parentheses.

Growth and somatic indices

Weight

Table S62: Comparison of baseline model and model with maturation indicator for body weight

Model		AIC_c	w	$F_{383,382}$	p
Baseline	$\sqrt{\boldsymbol{W}}=(\boldsymbol{E}^3)+\boldsymbol{T}$	2019.8	0.0	29.245	<0.0001
Maturation	$\sqrt{\boldsymbol{W}}=(\boldsymbol{E}^3)+\boldsymbol{T}+\boldsymbol{M}$	1993.3	1.0		

Note: Differences for basic model from Table S9 is caused by missing values in *GSI* and therefore *M*.

Table S63. Beta coefficients for the best model

Effect	Effect size	Beta±SE	t	p
Intercept		17.646 ± 0.666	26.51	<0.0001
$\boldsymbol{E}$	12.66	54.186 ± 3.140	17.26	<0.0001
$\boldsymbol{E}^2$	0.32	1.368 ± 3.14	0.44	0.66384
$\boldsymbol{E}^3$	-1.16	-4.978 ± 3.171	-1.57	0.11731
$\boldsymbol{T}$	0.11	0.156 ± 0.056	2.81	0.00522
$\boldsymbol{M}$	-0.88	-3.760 ± 0.695	-5.41	<0.0001

Length

Table S64: Comparison of baseline model and model with maturation indicator for body length

**Model**		**AIC$_c$**	**w**	$F_{386,385}$	p
Baseline	$\sqrt{L}=E$	176.6	0.0	42.581	<0.0001
Maturation	$\sqrt{L}=E+M$	138.0	1.0		

Note: Differences for basic model from Table S12 is caused by missing values in *GSI* and therefore *M*.

Table S65. Beta coefficients for the best model

**Effect**	**Effect size**	**Beta±SE**	**t**	**p**
Intercept		5.030 ± 0.032	155.90	<0.0001
E	0.64	0.086 ± 0.005	17.21	<0.0001
M	-1.05	-0.411 ± 0.063	-6.53	<0.0001

K factor

Table S66: Comparison of baseline model and model with maturation indicator for K factor

**Model**		**AIC$_c$**	**w**	$F_{383,382}$	p
Baseline	$K = E + T + E{:}(T^2)$	-608.5	0.20	4.82	0.02873
Maturation	$K = E + T + E{:}(T^2) + M$	-611.3	0.80		

Note: Differences for basic model from Table S14 is caused by missing values in *GSI* and therefore *M*.

Table S67. Beta coefficients for the best model

**Effect**	**Effect size**	**Beta±SE**	**t**	**p**
Intercept		1.078 ± 0.051	20.97	< 0.0001
E	0.27	0.011 ± 0.002	5.73	< 0.0001
T	0.19	0.014 ± 0.004	3.21	0.00144
M	0.40	0.053 ± 0.024	2.20	0.02873
$E : T$	-1.44	-0.060 ± 0.038	-1.58	0.11444
$E : T^2$	-1.89	-0.073 ± 0.017	-4.26	< 0.0001

HSI

Table S68: Comparison of baseline model and model with maturation indicator for HSI

**Model**		**AIC$_c$**	**w**	$F_{383,382}$	p
Baseline	$HSI = (E^2) + T + T{:}E$	-425.3	0.68	0.60	0.4405
Maturation	$HSI = (E^2) + T + T{:}E + M$	-423.8	0.32		

Note: Differences for basic model from Table S20 is caused by missing values in *GSI* and therefore *M*.

Gastrointestinal transit and gall bladder index

Stomach

Table S69: Comparison of baseline model and model with maturation indicator for stomach transit: nonlinear model

Model		AIC_c	w	$F_{206,205}$	*p*
Baseline	$STOM=(a+b\times T+c\times H+d\times T\times H)^2$	-119.6	0.0	22.09	<0.0001
Maturation	$STOM=(a+b\times T+c\times H+d\times T\times H+\mu\times M)^2$	-139.0	1.0		

Note: Nonlinear quadratic model was fitted for compatibility with the baseline analysis in Table S25

Table S70. Beta coefficients for the quadratic model

Effect		Beta±SE	t	p
a		0.739 ± 0.055	13.48	<0.0001
b		0.019 ± 0.005	4.12	<0.0001
c		-0.016 ± 0.008	-1.90	0.05886
d		-0.002 ± 0.001	-2.97	0.00334
μ		-0.187 ± 0.045	-4.21	<0.0001

Table S71: Comparison of baseline model and model with maturation indicator for stomach transit: best fitting exponential decay model (see Table S26)

Model		AIC_c	w	$F_{205,204}$	*p*
Baseline	$STOM=y_f+(y_0-y_f)e^{(-\alpha\times H+\beta\times T+\gamma\times t\times T)}$	-114.2	0.0	20.65	<0.0001
Maturation	$STOM=y_f+(y_0-y_f)e^{(-\alpha\times H+\beta\times T+\gamma\times t\times T+\mu\times M)}$	-132.3	1.0		

Table S72. Beta coefficients for the exponential decay model

Effect		Beta±SE	t	p
y_0		0.578 ± 0.078	7.39	<0.0001
y_f		-0.261 ± 0.105	-2.50	0.013341
α		0.037 ± 0.015	2.44	0.015536
β		0.029 ± 0.008	3.52	0.000524
γ		-0.003 ± 0.001	-2.48	0.014050
μ		-0.312 ± 0.090	3.48	0.000612

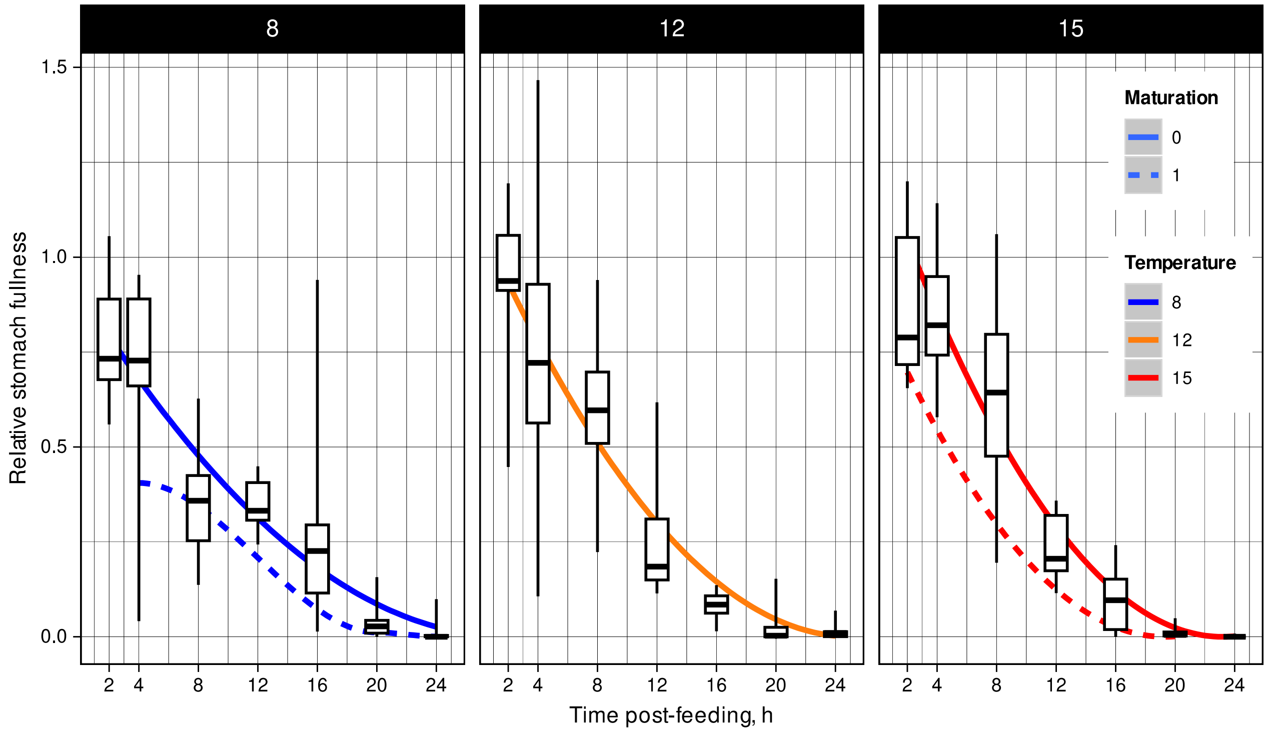

Figure S32. *Dynamics of relative stomach transit with fitted line from the best quadratic model (see Table S70), with maturation indicator*

Midgut

Table S73: Comparison of baseline model and model with maturation indicator for midgut transit

**Model**		**AIC$_c$**	**w**	$F_{203,202}$	***p***
Baseline	$MIDG = (H^2) + T + H{:}T$	-816.1	0.0	22.366	<0.0001
Maturation	$MIDG = (H^2) + T + M + H{:}T$	-835.8	1.0		

Table S74. Beta coefficients for the best model

**Effect**	**Effect size**	**Beta±SE**	**t**	**p**
Intercept		0.179 ± 0.009	19.47	<0.0001
H	-3.65	0.325 ± 0.134	2.42	0.016475
H^2	-8.31	-0.410 ± 0.032	-12.88	<0.0001
T	-0.32	-0.001 ± 0.001	-0.99	0.319445
M	-0.93	-0.046 ± 0.010	-4.73	<0.0001
$T{:}H$	-0.18	-0.0003 ± 0.0001	-3.86	0.000152

Hindgut

Table S75: Comparison of baseline model and model with maturation indicator for hindgut transit

**Model**		**AIC$_c$**	**w**	$F_{203,202}$	***p***
Baseline	$HDG = (H^2) \times T$	-964.0	0.23	4.43	0.03665
Maturation	$HDG = (H^2) \times T + M$	-966.4	0.77		

Table S76. Beta coefficients for the best model

Effect	Effect size	Beta±SE	t	p
Intercept		5.9e-02 ± 6.9e-03	8.71	<0.0001
H	-2.26	1.1e-01 ± 9.9e-02	-1.13	0.257929
H²	-3.15	3.8e-01 ± 9.9e-02	3.84	0.000166
T	0.001	9.9e-06 ± 5.7e-0	0.02	0.986298
M	-0.59	-1.5e-02 ± 7.2e-03	-2.10	0.036651
T:H	0.52	4.7e-03 ± 8.3e-03	0.57	0.572593
T:H²	-4.41	-4.0e-02 ± 8.3e-03	-4.80	<0.0001

Gall bladder index

Table S77: Comparison of baseline model and model with maturation indicator for gall bladder index

Model		AIC_c	w	$F_{202,201}$	*p*
Baseline	***GBI = (H³) × T***	-701.9	0.56	1.67	0.1973
Maturation	***GBI = (H³) × T + M***	-701.4	0.44		

Metabolites and ghrelin plasma levels

Glucose

Table S78: Comparison of baseline model and model with maturation indicator for *glucose*

Model		AIC_c	w	$F_{192,191}$	*p*
Baseline	***Glucose = H⁴ × T***	367.1	0.11	6.13	0.0142
Maturation	***Glucose = H⁴ × T + M***	363.0	0.89		

Table S79: Beta coefficients for the best model

Effect	Effect size	Beta±SE	t	p
Intercept		4.718 ± 0.167	28.25	<0.0001
H	4.67	-0.933 ± 2.446	0.38	0.703163
H²	3.64	9.428 ± 2.429	3.88	0.000143
H³	-4.67	1.541 ± 2.423	0.64	0.525583
H⁴	1.53	-1.757 ± 2.424	-0.73	0.469292
T	0.24	0.062 ± 0.014	4.45	<0.0001
M	-0.63	-0.460 ± 0.186	-2.48	0.014156
T:H	1.47	0.375 ± 0.206	1.82	0.069982
T:H²	-2.24	-0.576 ± 0.204	-2.83	0.005174
T:H³	-1.69	-0.432 ± 0.203	-2.13	0.034624
T:H⁴	0.97	0.250 ± 0.202	1.23	0.218037

Cholesterol

Table S80: Comparison of baseline model and model with maturation indicator for *cholesterol*

Model		AIC_c	w	$F_{198,197}$	*p*
Baseline	***Cholesterol = H² + T***	684.5	0.03	9.22	0.002724
Maturation	***Cholesterol = H² + T + M***	677.4	0.97		

Table S81: Beta coefficients for the best model

Effect	Effect size	Beta±SE	t	p
Intercept		7.218 ± 0.370	19.52	<0.0001
H	1.60	2.266 ± 1.302	1.74	0.083294
H²	4.25	6.171 ± 1.309	4.71	<0.0001
T	0.23	0.114 ± 0.031	3.67	0.000317
M	0.86	1.215 ± 0.400	3.04	0.002724

Lactate

Table S82: Comparison of baseline model and model with maturation indicator for *lactate*

Model		AIC_c	w	$F_{195,194}$	*p*
Baseline	$\sqrt{Lactate}=(\boldsymbol{H}^3)+\boldsymbol{T}+\boldsymbol{H}^2:\boldsymbol{T}$	-52.8	0.75	0.0	0.9951
Maturation	$\sqrt{Lactate}=(\boldsymbol{H}^3)+\boldsymbol{T}+\boldsymbol{H}^2:\boldsymbol{T}+\boldsymbol{M}$	-50.7	0.25		

Triglyceride

Table S83: Comparison of baseline model and model with maturation indicator for t*riglyceride*

Model		AIC_c	w	$F_{196,195}$	*p*
Baseline	***Trig = H + T:(H²)***	585.6	0.03	9.26	0.002661
Maturation	***Trig = H + T:(H²) + M***	578.5	0.97		

Table S84: Beta coefficients for the best model

Effect	Effect size	Beta±SE	t	p
Intercept		1.802 ± 0.407	4.431	<0.0001
H	0.08	0.129 ± 0.039	3.295	0.00117
M	0.64	0.975 ± 0.320	3.043	0.00266
T:H	-2.79	-1.090 ± 0.362	-3.013	0.00293
T:H²	-2.11	-0.581 ± 0.087	-6.653	<0.0001

Ghrelin

Table S85: Comparison of baseline model and model with maturation indicator for *ghrelin*

Model		AIC_c	w	$F_{201,200}$	*p*
Baseline	***GhRL = H² + T:H²***	1544.7	0.75	0.0	0.9857
Maturation	***GhRL = H² + T:H² + M***	1546.9	0.25		

Stomach mRNA expression levels

ghrl1

Table S86: Comparison of baseline model and model with maturation indicator for *ghrl1*

Model		AIC_c	w	$F_{206,205}$	*p*
Baseline	***ghrl1* = *H*² + *T*:*H***	2990.0	0.64	0.97	0.3249
Maturation	***ghrl1* = *H*² + *T*:*H* + *M***	2991.1	0.36		

ghrl2

Table S87: Comparison of baseline model and model with maturation indicator for *ghrl2*

Model		AIC_c	w	$F_{206,205}$	*p*
Baseline	***ghrl2* = *H*² + *T*:*H***	3919.8	0.74	0.00	0.9624
Maturation	***ghrl2* = *H*² + M + *T*:*H***	3922.0	0.26		

mboat

Table S88: Comparison of baseline model and model with maturation indicator for *ghrl2*

Model		AIC_c	w	$F_{206,205}$	*p*
Baseline	***mboat* = *H*²**	898.0	0.66	0.75	0.3879
Maturation	***mboat* = *H*² + *M***	899.3	0.34		

Hypothalamic mRNA expression levels

npya1

Table S89: Comparison of baseline model and model with maturation indicator for *npya1*

Model		AIC_c	w	$F_{197,196}$	*p*
Baseline	***npya1* = *H*² × *T***	2282.0	0.26	4.1998	0.04176
Maturation	***npya1* = *H*² × *T* + *M***	2279.9	0.74		

Table S90: Beta coefficients for the best model

Effect	Effect size	Beta±SE	t	p
Intercept		307.757 ± 19.195	16.03	<0.00001
H	5.84	90.808 ± 280.949	0.32	0.74688
***H*²**	0.56	719.419 ± 278.856	-2.58	0.01061
T	-0.30	-8.131 ± 1.604	-5.07	<0.00001
M	-0.54	-42.542 ± 20.759	-2.05	0.04176
T*:*H	1.16	31.905 ± 23.580	1.35	0.17759
***T*:*H*²**	2.36	65.484 ± 23.156	2.83	0.00517

npya2

Table S91: Comparison of baseline model and model with maturation indicator for *npya2*

Model		AIC_c	w	$F_{198,197}$	*p*
Baseline	***npya2 = H + T***	3308.0	0.46	2.41	0.1224
Maturation	***npya2 = H + T + M***	3307.6	0.54		

pomca1

Table S92: Comparison of baseline model and model with maturation indicator for *pomca1*

Model		AIC_c	w	$F_{198,197}$	*p*
Baseline	$ln(\mathbf{pomca1}) = \mathbf{H^2 + T}$	471.2	0.09	6.67	0.01055
Maturation	$ln(\mathbf{pomca1}) = \mathbf{H^2 + T + M}$	466.6	0.91		

Table S93: Beta coefficients for the best model

Effect	Effect size	Beta±SE	t	p
Intercept		6.729 ± 0.222	30.33	<0.00001
H	2.47	2.007 ± 0.771	2.60	0.00996
$\mathbf{H^2}$	-2.39	-1.949 ± 0.770	-2.53	0.01211
T	-0.17	-0.046 ± 0.018	-2.48	0.01400
M	-0.76	-0.613 ± 0.237	-2.58	0.01055

pomca2

Table S94: Comparison of baseline model and model with maturation indicator for *pomca2*

Model		AIC_c	w	$F_{195,194}$	*p*
Baseline	$ln(\mathbf{pomca2}) = \mathbf{H^2 + T}$	441.1	0.20	4.86	0.02872
Maturation	$ln(\mathbf{pomca2}) = \mathbf{H^2 + T + M}$	438.3	0.80		

Table S95: Beta coefficients for the best model

Effect	Effect size	Beta±SE	t	p
Intercept		6.578 ± 0.211	31.22	<0.00001
H	2.27	1.804 ± 0.740	2.44	0.0157
$\mathbf{H^2}$	-1.67	-1.348 ± 0.739	-1.82	0.0699
T	-0.33	-0.090 ± 0.018	-5.10	<0.00001
M	-0.63	-0.496 ± 0.225	-2.20	0.0287

agrp1

Table S96: Comparison of baseline model and model with maturation indicator for *agrp1*

Model		AIC_c	w	$F_{199,198}$	*p*
Baseline	$\sqrt{agrp1} = \mathbf{H}$	1432.4	0.0	28.34	<0.0001
Maturation	$\sqrt{agrp1} = \mathbf{H + M}$	1407.6	1.0		

Table S97: Beta coefficients for the best model

Effect	Effect size	Beta±SE	t	p
Intercept		25.987 ± 0.960	27.06	<0.00001
H	0.12	0.134 ± 0.073	1.82	0.0695
M	-0.35	-13.160 ± 2.472	-5.32	<0.00001

cart2b

Table S98: Comparison of baseline model and model with maturation indicator for *agrp1*

Model		AIC_c	w	$F_{199,198}$	*p*
Baseline	***cart2b* = *H***	4090.5	0.51	1.98	0.1614
Maturation	***cart2b* = *H* + *M***	4090.6	0.49		

REFERENCES

Burnham, K.P., Anderson, D.R., 2002. Model selection and multimodel inference: a practical information-theoretic approach, 2. ed., [4. printing]. ed. Springer, New York, NY.

Chambers, J.M., Hastie, T.J. (Eds.), 1997. Statistical models in S. Chapman & Hall, London.

Claeskens, G., Hjort, N.L., 2008. Model selection and model averaging, Cambridge series in statistical and probabilistic mathematics. Cambridge University Press, Cambridge.

Faraway, J.J., 2009. Linear Models with R. Chapman and Hall/CRC, Boca Raton, FL.

Fox, J., 2016. Applied regression analysis and generalized linear models, 3rd ed. Sage, London.

Pino Martinez, E., Balseiro, P., Pedrosa, C., Haugen, T.S., Fleming, M.S., Handeland, S.O., 2021. The effect of photoperiod manipulation on Atlantic salmon growth, smoltification and sexual maturation: A case study of a commercial RAS. Aquaculture Research 52, 2593–2608. https://doi.org/10.1111/are.15107

Pino Martinez, E., Imsland, A.K.D., Hosfeld, A.-C.D., Handeland, S.O., 2023. Effect of photoperiod and transfer time on Atlantic salmon smolt quality and growth in freshwater and seawater aquaculture systems. Fishes 8, 212. https://doi.org/10.3390/fishes8040212

R Core Team, 2025. R: A Language and Environment for Statistical Computing.
